# Intra-Arterial Tenecteplase After Successful Reperfusion in Large Vessel Occlusion Stroke: A Randomized Clinical Trial

**DOI:** 10.1001/jamaneurol.2025.2036

**Published:** 2025-07-05

**Authors:** Xianhua Hou, Jiacheng Huang, Li Wang, Yuxuan He, Jiaxing Song, Changwei Guo, Shihai Yang, Xiaolei Shi, Lin Chen, Qu Liu, Junfeng Su, Lin Zeng, Maojun Jiang, Boyu Chen, Xiangping Cheng, Shengli Chen, Honghua Pan, Xiaoping Shen, Youlin Wu, Xionglin Tang, Jian Wang, Shibo Han, Tianqiang Pu, Changchuan Wu, Fengguang Li, Lunxue Qu, Zhong Fu, Hua Liu, Yu Li, Bin Mei, Yanbo Cheng, Zicheng Hu, Haochun Zhang, Tao Lv, Min Wu, Ruchuang Xu, Qinglin Ye, Liangbo Kong, Shuai Mi, Junhua Wu, Yu Wang, Zhenxuan Tian, Wenzhe Sun, Jinfu Ma, Xu Xu, Yazhou Wu, Duolao Wang, Raul G. Nogueira, Thanh N. Nguyen, Jeffrey L. Saver, Wenjie Zi, Zhenhua Zhou

**Affiliations:** 1 Department of Neurology, The First Affiliated Hospital (Southwest Hospital), Army Medical University, Chongqing, China; 2 Department of Neurology, The Second Affiliated Hospital (Xinqiao Hospital), Army Medical University, Chongqing, China; 3 Department of Neurology, The Second Affiliated Hospital of Chongqing Medical University, Chongqing, China; 4 Department of Neurology, Zigong Third People’s Hospital, Zigong, China; 5 Department of Neurology, The First Affiliated Hospital of University of South China, Hengyang, China; 6 Department of Neurology, Jingzhou Central Hospital, Jingzhou, China; 7 Department of Neurology, The People’s Hospital of QianNan, Duyun, China; 8 Department of Neurology, Qujing First People’s Hospital, Qujing, China; 9 Department of Neurology, Gulin People’s Hospital, Gulin, China; 10 Department of Neurology, Three Gorges Hospital affiliated to Chongqing University, Wanzhou, China; 11 Department of Neurology, Guilin People’s Hospital, Guilin, China; 12 Department of Neurology, JiuJiang No. 1 People’s Hospital, Jiujiang, China; 13 Department of Neurology, Chongzhou People’s Hospital, Chongzhou, China; 14 Department of Neurology, Affiliated Hospital of Youjiang Medical University for Nationalities, Youjiang, China; 15 Department of Neurology, Ya’an People’s Hospital, Ya’an, China; 16 Department of Neurology, People’s Hospital of Dali Prefecture, Dali, China; 17 Department of Neurology, Guangyuan Central Hospital, Guangyuan, China; 18 Department of Neurology, Chengdu Second People’s Hospital, Chengdu, China; 19 Department of Neurology, Wuhan Puren Hospital, Wuhan, China; 20 Department of Neurology, Chongqing Changshou District People’s Hospital, Changshou, China; 21 Department of Neurology, The People’s Hospital of Jianyang, Jianyang, China; 22 Department of Neurology, The Third People’s Hospital of Chengdu, Chengdu, China; 23 Department of Neurology, Qianjiang Central Hospital of Chongqing, Qianjiang, China; 24 Department of Neurology, Zhongnan Hospital of Wuhan University, Wuhan, China; 25 Department of Neurology, The Affiliated Hospital of Xuzhou Medical University, Xuzhou, China; 26 Department of Neurology, Chongqing Hechuan District People’s Hospital, Hechuan, Chongqing, China; 27 Department of Neurology, Dazhou Central Hospital, Dazhou, China; 28 Department of Health Statistics, College of Preventive Medicine, Army Medical University, Chongqing, China; 29 Global Health Trials Unit, Liverpool School of Tropical Medicine, Liverpool, United Kingdom; 30 UPMC Stroke Institute, Departments of Neurology and Neurosurgery, University of Pittsburgh School of Medicine, Pittsburgh, Pennsylvania; 31 Departments of Radiology & Neurology, Boston Medical Center, Boston, Massachusetts; 32 Department of Neurology and Comprehensive Stroke Center, David Geffen School of Medicine, University of California, Los Angeles, Los Angeles, California

## Abstract

**Importance:**

The optimal dose, safety, and efficacy of intra-arterial tenecteplase after successful reperfusion by endovascular thrombectomy for large vessel occlusion (LVO) is unknown.

**Objective:**

To evaluate the dose-dependent adverse events and signals of efficacy of intra-arterial tenecteplase in LVO after successful reperfusion with thrombectomy, defined as an Extended Treatment in Cerebral Infarction score of 2b-3.

**Design, Setting, and Participants:**

This open-label, blinded-outcome assessment trial, incorporating a 14 + 8 dose-escalation (phase 1b, nonrandomized) and dose-expansion (phase 2a, randomized) design, was conducted in China between 2023 and 2024, with follow-up continuing through November 2024. This was a multicenter clinical trial including patients with LVO and successful reperfusion within 24 hours of last known well.

**Interventions:**

In phase 1b, intra-arterial tenecteplase, 0.0313, 0.0625, 0.1250, 0.1875 mg/kg; in phase 2a, intra-arterial tenecteplase 0.0313 or 0.0625 mg/kg, or control (without intra-arterial thrombolysis).

**Main Outcomes and Measures:**

The primary outcome in phase 1b was symptomatic intracranial hemorrhage (sICH) within 24 hours. The primary outcome in phase 2a was 90-day no-disability outcome (modified Rankin Scale score 0-1).

**Results:**

A total of 205 patients (phase 1b: 48, phase 2a: 157) were enrolled and analyzed. The median (IQR) age was 71 (60-77) years, and 113 (55.1%) were male. In phase 1b, 1 of 14 and 2 of 22 patients with sICH were observed at dose tiers 0.0313 and 0.0625 mg/kg, respectively. Three of 12 patients had sICH at dose tier 0.1250 mg/kg, exceeding the prespecified safety threshold (*P* = .04). In phase 2a, eligible patients were randomly assigned to receive tenecteplase, 0.0313 mg/kg (n = 46) and 0.0625 mg/kg (n = 46), and 65 patients composed the control group. The primary outcome occurred in 22 of 65 patients (33.8%) in the control group, 17 of 46 patients (37.0%) in the tenecteplase, 0.0313 mg/kg, group (adjusted risk ratio [RR] vs control, 0.85; 95% CI, 0.54-1.35; *P* = .50), and 20 of 46 patients (43.5%) in the tenecteplase, 0.0625 mg/kg, group (adjusted RR, 1.15; 95% CI, 0.73-1.80; *P* = .55). No significant difference in the safety outcomes was observed among the 3 groups.

**Conclusions and Relevance:**

Results of this phase 1 and 2 randomized clinical trial reveal that adjunctive intra-arterial tenecteplase dosages of 0.0313 mg/kg or 0.0625 mg/kg after successful reperfusion in patients with anterior circulation LVO showed adequate safety to advance to larger trials to determine the potential therapeutic benefits.

**Trial Registration:**

ChiCTR.org.cn Identifier: ChiCTR2300073787 and ChiCTR2400080624

## Key Points

**Question:**

Which of 3 doses of the clot-dissolving drug tenecteplase given by catheter to patients with stroke after successful endovascular thrombectomy shows sufficient safety and signals of potential efficacy to advance to a pivotal trial?

**Findings:**

In the first phase of the study, enrolling 48 patients, rates of symptomatic brain hemorrhage were below the acceptable threshold for the low-dose and intermediate-dose tiers but not for the high-dose tier. In the second trial phase, enrolling 157 patients, the 90-day no-disability outcome was not significantly different among the control, low-dose, and medium-dose groups.

**Meaning:**

In this phase 1 and 2 randomized clinical trial including patients with acute ischemic stroke after successful endovascular thrombectomy, tenecteplase given by catheter at low dose and medium dose showed adequate safety to advance to larger trials to determine their potential therapeutic benefits.

## Teaser

This phase 1 and 2 randomized clinical trial investigated both the safety threshold and efficacy of 3 different doses of tenecteplase when given by catheter to patients with large vessel occlusion stroke after successful endovascular thrombectomy.

## Introduction

Stroke is one of the leading causes of morbidity and mortality worldwide.^1,2^ Endovascular thrombectomy (EVT) has been recommended as the optimal treatment for patients with acute ischemic stroke due to large vessel occlusion (LVO).^3,4,5,6,7^ A meta-analysis^8^ of individual patient data from 5 randomized clinical trials indicated that 75% of patients achieved successful reperfusion. However, only 27% were free of disability at 90 days among these patients.^7^ The discrepancy between successful reperfusion and clinical outcomes is at least in part attributable to irreversible damage that occurs in brain tissue before reperfusion. But additionally, even after successful reperfusion of the large vessels, patients with Extended Treatment in Cerebral Infarction (eTICI) score 2b and eTICI 2c have residual occlusions in medium vessels that continue to produce ischemia. In addition, even patients with eTICI 3 outcomes can have partial or complete failure of reperfusion in the microcirculation, known as the *no-reflow phenomenon* (NRP), which may also significantly contribute to this discordance.^9,10^ In situ thrombosis in the microcirculatory and distal embolization from the proximal cerebral arteries are important contributors for the NRP according to the experimental animal models of cerebral ischemia and reperfusion.^11^ As the efficacy of thrombolysis is related to the target thrombus size, theoretically, these smaller thrombi would be more suitable for pharmacologic dissolution than larger proximal thrombi.^12^

The Effect of Intra-Arterial Alteplase vs Placebo Following Successful Thrombectomy on Functional Outcomes in Patients With Large Vessel Occlusion Acute Ischemic Stroke (CHOICE) trial, which investigated the effects of intra-arterial alteplase vs placebo after successful thrombectomy in patients with LVO acute ischemic stroke, demonstrated the potential benefit of administering intra-arterial alteplase at a dose equivalent to one-quarter of the standard intravenous thrombolytic dose.^13^ To our knowledge, studies that include a dose-escalation exploratory component are limited, leaving the optimal dose that balances efficacy and safety undetermined.

Tenecteplase is a modified form of alteplase that has been shown to have equal safety and better functional outcomes compared with alteplase.^14^ Given the differences of tenecteplase compared with alteplase, adjunctive intra-arterial administration of tenecteplase may provide greater benefit for patients with LVO stroke after successful EVT. However, the optimal dose, efficacy, and safety of intra-arterial tenecteplase after successful reperfusion is unknown. The Safety and Efficacy of Adjunctive Intra-Arterial Tenecteplase Following Successful Thrombectomy in Patients With Large Vessel Occlusion (DATE) trial was a phase 1b and 2a clinical trial designed to evaluate the safety and efficacy of adjunctive intra-arterial tenecteplase after successful reperfusion by EVT in patients with anterior circulation LVO presenting within 24 hours of symptom onset.

## Methods

### Study Design

The DATE study was a prospective, multicenter, open-label, blinded-outcome assessment, phase 1b and 2a randomized clinical trial. A condensed study protocol has been published; the full protocol is available in Supplement 1,^15^ and the statistical analysis plan is available in Supplement 2. The DATE study was conducted at 30 sites in China (eFigure 1 in Supplement 3). This trial was divided into 2 parts: the first part (phase 1b) was a pilot dose-escalation safety study using a 4-dose tier, 14 + 8 design, and the second part (phase 2a) was an exploratory study to assess the safety and potential efficacy of adjunctive intra-arterial tenecteplase after successful EVT in patients with LVO stroke at 2 different doses (eMethods 1 in Supplement 3). After the completion of phase 1b, a data safety monitoring board (DSMB) and investigators selected 2 doses (A or B) to be tested in phase 2a according to the initial safety results. The trial protocol was approved by a central medical ethics committee and the research board of each participating center. All enrolled patients or their legally authorized representative provided written informed consent.

The trial was designed and conducted by a steering committee that included independent academic investigators (eAppendix in Supplement 3). The trial was overseen by an independent DSMB. All outcome adjudications were performed by an independent clinical events committee. The trial was conducted according to the Declaration of Helsinki Harmonization Guidelines. This study adhered to the Consolidated Standards of Reporting Trials (CONSORT) reporting guidelines.

### Participants

Eligible patients were aged 18 years or older with acute LVO ischemic stroke in the anterior circulation presenting within 24 hours of time last known well, had a baseline National Institutes of Health Stroke Scale (NIHSS) score of 6 to 24, had a baseline Alberta Stroke Program Early CT Score (ASPECTS) of at least 6 on noncontrast computed tomography, had an occlusion of the intracranial internal carotid artery, the first segment of the middle cerebral artery (M1), or the second segment of the middle cerebral artery (M2), and had an eTICI score of 2b to 3 after EVT.^8^ Patients treated with intravenous thrombolysis before EVT were excluded. Detailed inclusion and exclusion criteria are provided in Supplement 1 and eMethods 2 in Supplement 3. Patient race and ethnicity data were included in this study to show generalizability of findings across different racial or ethnic groups. Patients self-reported the following ethnicities: Bouyei, Han, Yi, and Zhuang. Race, ethnicity, and sex were reported by the patient and verified by identification card.

### Randomization, Blinding, and Masking

The phase 1b trial was a nonrandomized dose-escalation trial. In phase 2a, eligible patients were randomized with stratified allocation according to 2 parameters: patient age (<70 vs ≥70 years) and admission NIHSS score (<15 vs ≥15), to receive 1 of the 2 selected doses of tenecteplase or to the control group in a 1:1:√2 ratio, which, in turn, yielded probabilities of assignment of 0.293, 0.293, and 0.414, respectively. Patients were randomly assigned by a real-time internet-based system. This process was automated, which allowed for concealment of the sequence of allocation. The study team members were blinded to the treatment randomization. Definition of the analyzed population is available in eMethods 3 in Supplement 3.

### Study Intervention

In phase 1b, eligible participants received intra-arterial administration of increasing doses of tenecteplase after successful EVT. Patients were treated with up to 4 escalating dose tiers: 0.0313 mg/kg (1/8 intravenous dose), 0.0625 mg/kg (1/4 intravenous dose), 0.1250 mg/kg (1/2 intravenous dose), and 0.1875 mg/kg (3/4 intravenous dose). In phase 2a, 2 doses (A and B) were chosen by the DSMB and investigators jointly based on the results of the phase 1b study.

Eligible patients assigned to the tenecteplase group underwent an infusion of intra-arterial tenecteplase with the assigned dose within 10 minutes after randomization. This infusion was administered through a distal access catheter or microcatheter positioned proximal to the initially occluded artery. For terminal internal carotid artery (ICA) occlusions, the infusion was proximal to the ICA terminus. For M1 or M2 occlusions, the infusion was proximal to the M1 or M2 segment, respectively. Catheter positioning was guided by real-time angiographic visualization to confirm proximity to the initial occlusion site. In patients allocated to the control group, the procedure was terminated without further intra-arterial thrombolysis.

All patients were monitored in the acute stroke unit and could be admitted to the intensive care unit if necessary. All enrolled patients underwent standardized medical treatment and subsequent secondary preventive treatment according to the Chinese Guidelines for Endovascular Treatment of Acute ischemic Stroke 2018.^16^

### Outcomes

In phase 1b, the primary outcome was the proportion of patients with symptomatic intracranial hemorrhage (sICH) within 24 hours according to the European Cooperative Acute Stroke Study III (ECASS III) bleeding classification^17^ evaluated by the central imaging core laboratory in a blinded manner. Other secondary safety outcomes included any ICH within 24 hours, mortality within 90 days, and systemic bleeding. In phase 2a, the primary outcome was the proportion of patients with a no-disability outcome (modified Rankin Scale [mRS] score of 0 or 1) at 90 (±14) days. Primary outcome assessments were performed at 90 (±14) days by 2 independent, certified, central rater physicians who were blinded to the treatment details. To maintain the reliability, accessibility, and traceability of the mRS score, we retained a video or audio records of the 90-day follow-up for all patients. If video or audio recordings were unavailable, outcomes were determined in person by certified local investigators, who were also unaware of the treatment assignment. The secondary outcomes were the proportion of patients with functional independence (mRS score of 0 to 2) at 90 (±14) days, level of disability (ordinal distribution of mRS scores) at 90 days, favorable shift in reperfusion on the eTICI score after intra-arterial tenecteplase thrombolysis therapy (final angiographic reperfusion status was assessed immediately after recanalization, and angiographic improvement was not applicable in the control group; in the tenecteplase group, postlysis angiographic control runs were performed after completion of thrombolytic administration to evaluate reperfusion improvement), change in NIHSS score from baseline to day 5 through 7, and health-related quality of life (European Quality of Life 5-Dimension 3-Level scale [EQ-5D-3L] score) at 90 days (characteristics of EQ-5D-3L value sets in China are available in eMethods 4 in Supplement 3). All prerandomization and final post–intra-arterial (intra-arterial tenecteplase group) angiograms were scored at a core laboratory by central and blinded reviewers using the eTICI and classified as eTICI 2b, eTICI 2c, and eTICI 3. The posttreatment angiograms were scored using eTICI and classified as improved, worsened, or unchanged compared with the pre–intra-arterial infusion eTICI.

### Sample Size Calculation

For dose escalation (phase 1b), according to the Chinese Acute Anterior Circulatory Ischemic Stroke Endovascular Treatment Registry Study, 13.8% of patients experienced sICH within 24 hours of receiving EVT.^18^ Using sample sizes typical for dose-escalation phase 1 clinical trials,^19,20^ we used a 14 + 8 design scheme with the occurrence of sICH within 24 hours after EVT as the dose-limiting toxic effect. At each tier, 14 patients were enrolled, beginning with the lowest prespecified dose. If fewer than 2 of 14 patients developed sICH, the trial advanced to the next tier dose. If 2 of the 14 patients developed sICH, 8 more patients were enrolled at that dose. If 0 to 1 of the additional 8 patients developed sICH, the trial advanced to the next tier dose. If 2 of the additional 8 patients developed sICH or 3 of the first 14 developed sICH, that dose was deemed not tolerated and the immediately preceding dose was the estimated maximum tolerated dose.

For dose expansion (phase 2a), patients enrolled were assigned to 1 of 3 groups: the dose A group, the dose B group, and the control group.

### Statistical Analysis

The primary analysis of the primary outcomes was based on the full analysis set of all patients as randomized. A per-protocol sensitivity analysis was planned that would exclude patients with major protocol violations. The treatment effect for the primary outcome and binary outcomes was measured using a risk ratio (RR) by fitting the modified Poisson regression.^21^ The treatment effect of the ordinal mRS score was estimated using the generalized odds ratio (GenOR).^22^ Nonnormal continuous secondary outcomes were analyzed using the win ratio (WR) approach.^23^ The primary analyses for all outcomes were based on adjusted analyses for 5 prespecified covariates: age, baseline NIHSS score, baseline ASPECTS, occlusion site, and time from last known well to enrollment, generating the point estimates of adjusted treatment effects with their 95% CIs. The adjusted RRs were estimated by adding those covariates into modified Poisson regression. The adjusted GenOR and WR were estimated using the inverse probability of treatment weighting method. In addition, unadjusted treatment effects were calculated and reported. A prespecified pooled analysis of phase 1a and 2b with each dose tier was combined to conduct a supportive analysis.

For all outcomes, a 2-sided *P* value <.05 was considered to indicate statistical significance. All analyses of safety outcomes and secondary outcomes were considered exploratory and performed without adjustment for multiplicity. Analyses were performed with SAS software, version 9.4 (SAS Institute), and R, version 4.1 (R Foundation for Statistical Computing).

## Results

### Study Population

**Figure 1.**
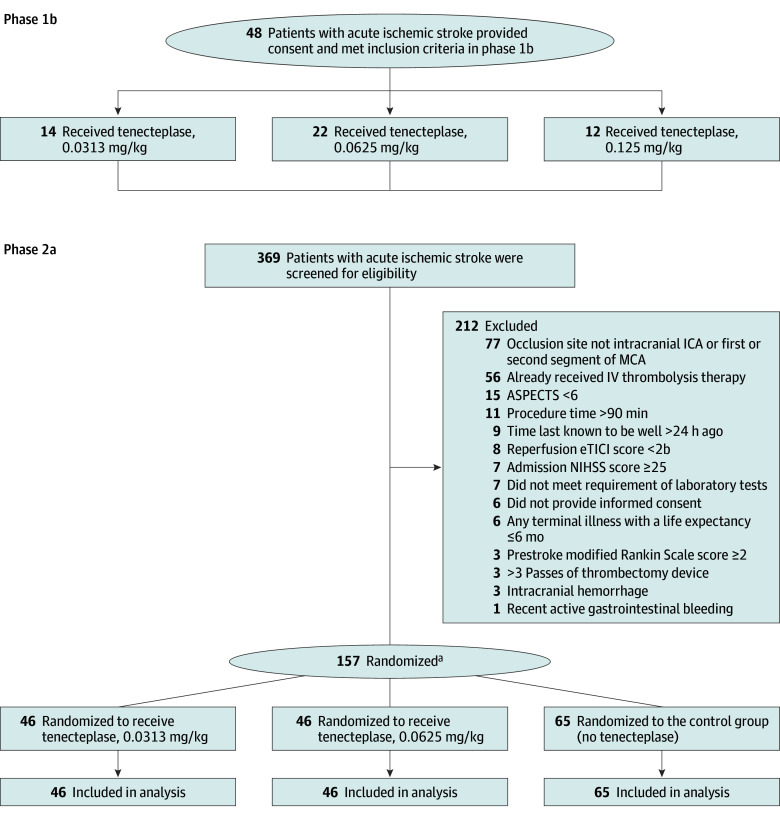
Flowchart of Patients Through the Successful Thrombectomy in Patients With Large Vessel Occlusion (DATE) Trial ASPECTS indicates Alberta Stroke Program Early CT Score; CT, computed tomography; eTICI, Extended Treatment in Cerebral Infarction; ICA, internal carotid artery; IV, intravenous; MCA, middle cerebral artery; NIHSS, National Institutes of Health Stroke Scale. ^a^Eligible patients were randomized with stratification allocation according to 2 strata: patient age (<70 vs ≥70 years), and admission NIHSS score (<15 vs ≥15).

A total of 205 patients (phase 1b: 48, phase 2a: 157; median [IQR] age, 71 [60-77] years; 92 female [44.9%]; 113 male [55.1%]) were enrolled and analyzed. Between July 2023 and December 2023, 48 patients were enrolled in phase 1b at 7 sites and assigned to receive the dose-escalation groups. Fourteen patients were enrolled for the first tier and received a tenecteplase dose of 0.0313 mg/kg; 1 patient experienced sICH within 24 hours. Therefore, the next dose level of 0.0625 mg/kg was implemented, enrolling 14 patients. However, 2 of 14 patients experienced dose-limiting toxicity. Subsequently, 8 additional patients were enrolled at the same dose, and no sICH occurred. When increasing the dose to 0.1250 mg/kg, 3 of 12 enrolled participants experienced sICH. Having exceeded the safety threshold, that dose was deemed not tolerated, and the immediately preceding dose was deemed the estimated maximum tolerated dose. Accordingly, the tenecteplase doses of 0.0313 mg/kg and 0.0625 mg/kg advanced to exploration in phase 2a. Patients flows for both study phases are shown in Figure 1, and the characteristics of the phase 1b population by group are listed in eTable 1 in Supplement 3. Baseline characteristics of the pooled analysis are in eTable 2 in Supplement 3.

From February 2024 to August 2024, 369 patients with acute ischemic stroke were screened at 23 study sites, including 157 patients (42.5%) who were randomized in phase 2a to receive either tenecteplase, 0.0313 mg/kg (n = 46), 0.0625 mg/kg (n = 46), or control (n = 65). The median age was 71 (60-77) years; 68 participants (43.3%) were female, and 89 (56.7%) were male. All 157 patients self-reported Asian race. Patients reported the following ethnicities: 7 Bouyei (4.5%), 144 Han (91.7%), 1 Yi (0.6%), and 5 Zhuang (3.2%). The baseline median (IQR) ASPECTS was 8 (6-9) in the tenecteplase, 0.0625 mg/kg, dose-level group, 9 (8-9) in the tenecteplase, 0.0313 mg/kg, dose-level group, and 8 (7-9) in the control group. The baseline characteristics were similar across the groups, except for a significantly higher proportion of intracranial internal carotid artery occlusions in the control group (Table 1).

**Table 1.** Baseline Characteristics of the Phase 2a Population by Group

Characteristic	No. (%)		
	Tenecteplase, 0.0625 mg/kg (n = 46)	Tenecteplase, 0.0313 mg/kg (n = 46)	Control (n = 65)
Age, median (IQR), y	71 (61-77)	71 (60-76)	71 (56-78)
Race^a^			
Asian	46 (100)	46 (100)	65 (100)
Ethnicity^a^			
Bouyei	3 (6.5)	3 (6.5)	1 (1.5)
Han	43 (93.5)	39 (84.8)	62 (95.4)
Yi	0	1 (2.2)	0
Zhuang	0	3 (6.5)	2 (3.1)
Sex^a^			
Female	17 (37.0)	16 (34.8)	35 (53.8)
Male	29 (63.0)	30 (65.2)	30 (46.2)
Medical history^b^			
Hypertension	26 (56.5)	31 (67.4)	40 (61.5)
Atrial fibrillation	18 (39.1)	20 (43.5)	36 (55.4)
Hyperlipidemia	16 (34.8)	14 (30.4)	19 (29.2)
Diabetes	15 (32.6)	11 (23.9)	15 (23.1)
Smoking^c^	15 (32.6)	16 (34.8)	14 (21.5)
Stroke	11 (23.9)	7 (15.2)	13 (20.0)
Prestroke modified Rankin Scale score^d^			
0	41 (89.1)	43 (93.5)	62 (95.4)
1	5 (10.9)	3 (6.5)	3 (4.6)
Baseline NIHSS score, median (IQR)^e^	17 (12-20)	17 (10-20)	17 (12-20)
Baseline ASPECTS, median (IQR)^f^	8 (6-9)	9 (8-9)	8 (7-9)
Systolic blood pressure at hospital arrival, median (IQR), mm Hg	141 (125-161)	145 (126-168)	138 (127-155)
Blood glucose level at hospital arrival, mmol/L^g^			
No.	39	40	53
Median (IQR)	7.0 (5.7-9.0)	7.2 (5.9-8.1)	7.1 (5.9-8.2)
Occlusion site			
Internal carotid artery	8 (17.4)	5 (10.9)	25 (38.5)
M1 segment	33 (71.7)	33 (71.7)	35 (53.8)
M2 segment	5 (10.9)	8 (17.4)	5 (7.7)
Angiographic eTICI scores before randomization^h^			
2b	11 (23.9)	12 (26.1)	17 (26.2)
2c	15 (32.6)	18 (39.1)	21 (32.3)
3	20 (43.5)	16 (34.8)	27 (41.5)
Time from last known well, median (IQR), min			
To puncture	342 (185-623)	330 (205-648)	322 (232-635)
To randomization	410 (259-748)	404 (262-725)	384 (288-677)
To study drug treatment^i^	417 (263-761)	414 (305-730)	NA

Abbreviations: ASPECTS, Alberta Stroke Program Early CT Score; eTICI, Extended Treatment in Cerebral Infarction; NA, not available; NIHSS, National Institutes of Health Stroke Scale; TOAST, Trial of Org 10172 in Acute Stroke Treatment.

SI conversion: To convert glucose to milligrams per deciliter, divide by 0.0555.

^a^ Sex reported by the patient and verified by identification card.

^b^ Comorbidities based on family or patient report.

^c^ Current or within the prior 5 years.

^d^ Scores on the modified Rankin Scale of functional disability range from 0 (no symptoms) to 6 (death). Three patients in the intra-arterial tenecteplase group and 2 patients in the control group had a prestroke score on the modified Rankin Scale of 2 or more.

^e^ Scores on the NIHSS range from 0 to 42, with higher scores indicating more severe neurological deficits.

^f^ The ASPECTS is an imaging measure of the extent of ischemic stroke. Scores range from 0 to 10, with higher scores indicating a smaller infarct core. Listed are values for the core laboratory assessment.

^g^ Data on glucose at baseline were missing for 7 patients in the intra-arterial tenecteplase, 0.0625 mg/kg group; 6 patients in the intra-arterial tenecteplase, 0.0313 mg/kg, group; and 12 patients in the control group.

^h^ The eTICI scale is a reperfusion measure based on digital subtraction angiography, which ranges from 0 (no reperfusion) to 3 (complete reperfusion).

^i^ Study treatment refers to the application of intra-arterial tenecteplase therapy.

### Outcomes

**Figure 2.**
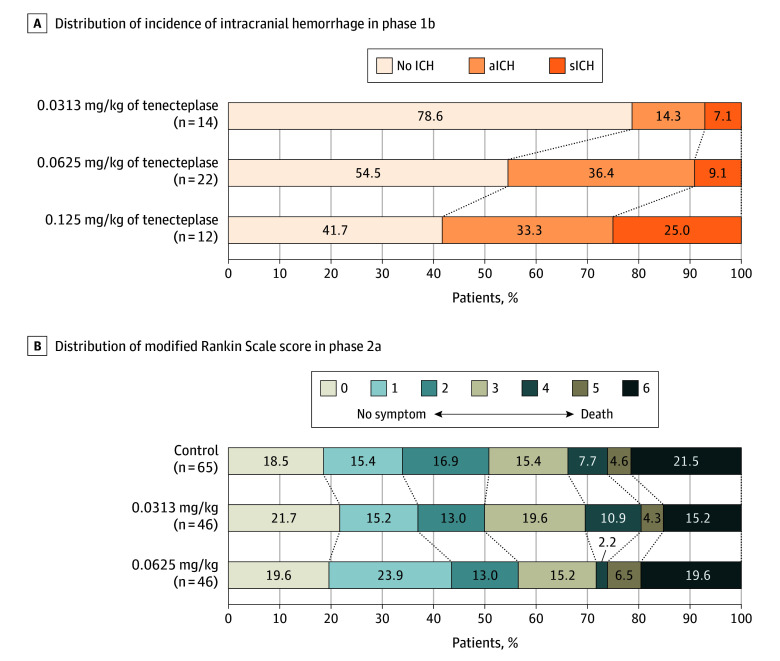
Intracranial Hemorrhage (ICH) Within 24 Hours in Phase 1b and Distribution of Score on the Modified Rankin Scale at 90 Days in Phase 2a A, Shown are the proportion of ICH within 24 hours of European Cooperative Acute Stroke Study III (ECASS III) hemorrhage classification. aICH denotes any intracranial hemorrhage; sICH, symptomatic intracranial hemorrhage. B, Shown are the distribution of the scores on the modified Rankin Scale among patients in the intra-arterial tenecteplase group and the control group. Scores range from 0 to 6, with 0 indicating no symptoms, 1, no clinically significant disability, 2, slight disability, 3, moderate disability, 4, moderately severe disability, 5, severe disability, and 6, death. Numbers indicate rounded proportions.

In phase 1b, the primary outcome of sICH within 24 hours occurred in 1 of 14 patients (7.1%) who received tenecteplase at a dose of 0.0313 mg/kg; 2 of 22 patients (9.1%) who received tenecteplase at a dose of 0.0625 mg/kg; and 3 of 12 patients (25.0%) who received tenecteplase at a dose of 0.1250 mg/kg (Figure 2A). The differences in sICH among the 3 groups are statistically significant (*P* = .04). There were no significant differences in the predefined secondary clinical efficacy outcomes among the groups. The details of the outcomes are presented in eTable 3 in Supplement 3.

In phase 2a, the primary efficacy outcome of the proportion of patients with an mRS score of 0 or 1 at 90 ± 14 days occurred in 22 of 65 patients (33.8%) in the control group, 17 of 46 patients (37.0%) with an intra-arterial tenecteplase dose of 0.0313 mg/kg (unadjusted RR vs control, 1.09; 95% CI, 0.66-1.81; *P* = .73; adjusted RR, 0.85; 95% CI, 0.54-1.35; *P* = .50), and 20 of 46 patients (43.5%) with an intra-arterial tenecteplase dose of 0.0625 mg/kg (unadjusted RR vs control, 1.28; 95% CI, 0.80-2.06; *P* = .30; adjusted RR, 1.15; 95% CI, 0.73-1.80; *P* = .55). The proportion of patients with mRS score of 0 through 2 at 90 days was 50.8% in patients allocated to the control group, 50.0% in patients treated with an intra-arterial tenecteplase dose of 0.0313 mg/kg (adjusted RR vs control, 0.91; 95% CI, 0.64-1.32), and 56.5% with an intra-arterial tenecteplase dose of 0.0625 mg/kg (adjusted RR vs control, 1.12; 95% CI, 0.80-1.56). The complete distribution of mRS scores at 90 days in the phase 1b and 1b+2a trials is displayed in eFigures 2 and 3 in Supplement 3, respectively.

Improved angiographic reperfusion was noted in 6.5% of patients receiving intra-arterial tenecteplase, 0.0625 mg/kg, and 8.7% of patients receiving intra-arterial tenecteplase, 0.0313 mg/kg. There were no significant differences in other prespecified secondary efficacy outcomes among groups. The results in the prespecified pooled analysis yielded similar results. The full distribution of outcomes across all 7 mRS levels is shown in Figure 2B. No patients had major protocol violations; therefore, the planned per-protocol sensitivity analysis was not needed.

### Adverse Events

In phase 1b, death occurred in 2 of 14 patients (14.3%) in the intra-arterial tenecteplase, 0.0313 mg/kg, group; in 5 of 22 patients (22.7%) in the intra-arterial tenecteplase, 0.0625 mg/kg, group; and in 4 of 12 patients (33.3%) in the intra-arterial tenecteplase, 0.1250 mg/kg, group.

In phase 2a, death occurred in 14 of 65 patients (21.5%) in the control group; in 7 of 46 patients (15.2%) in the intra-arterial tenecteplase, 0.0313 mg/kg, group (adjusted RR vs control, 0.77; 95% CI, 0.34-1.79); and in 9 of 46 patients (19.6%) in the intra-arterial tenecteplase, 0.0625 mg/kg, group (adjusted RR vs control, 0.78; 95% CI, 0.36-1.66). sICH occurred in 3.1% in the control group; 4.3% in the intra-arterial tenecteplase, 0.0313 mg/kg, group; and 6.5% in the intra-arterial tenecteplase, 0.0625 mg/kg, group. (Table 2). A complete list of adverse events is provided in eTables 4 to 7 in Supplement 3.

**Table 2.** Primary and Secondary Efficacy and Safety Outcomes

Outcome	Tenecteplase, 0.0625 mg/kg (n = 46)	Tenecteplase, 0.0313 mg/kg (n = 46)	Control (n = 65)	Treatment effect metric	Unadjusted value				Adjusted value^a^			
					Tenecteplase, 0.0625 mg/kg vs control (95% CI)	*P* value	Tenecteplase, 0.0313 mg/kg vs control (95% CI)	*P* value	Tenecteplase, 0.0625 mg/kg vs control (95% CI)	*P* value	Tenecteplase, 0.0313 mg/kg vs control (95% CI)	*P* value
Primary outcome^b^												
mRS score of 0 to 1 at 90 d	20 (43.5)	17 (37.0)	22 (33.8)	RR	1.28 (0.80 to 2.06)	.30	1.09 (0.66 to 1.81)	.73	1.15 (0.73 to 1.80)	.55	0.85 (0.54 to 1.35)	.50
Secondary outcomes												
mRS score of 0 to 2 at 90 d^b^	26 (56.5)	23 (50.0)	33 (50.8)	RR	1.11 (0.79 to 1.58)	.55	0.98 (0.68 to 1.43)	.94	1.12 (0.80 to 1.56)	.52	0.91 (0.64 to 1.32)	.63
mRS score at 90 d, median (IQR)^c^^,^^d^	2 (1 to 5)	3 (1 to 4)	2 (1 to 5)	GenOR	1.19 (0.71 to 1.98)	.51	1.13 (0.68 to 1.87)	.64	1.18 (0.70 to 1.99)	.53	0.95 (0.55 to 1.62)	.84
Improved angiographic reperfusion^e^	3 (6.5)	4 (8.7)	NA	NA	NA	NA	NA	NA	NA	NA	NA	NA
Excluding baseline eTICI scores of 3	3/26 (11.5)	4/30 (13.3)	NA	NA	NA	NA	NA	NA	NA	NA	NA	NA
Change of NIHSS score at 5-7 d or discharge if earlier, from baseline, median (IQR)^f^^,^^g^	−7 (−12 to 0)	−6 (−12 to −2)	−6 (−13 to 0)	WR	0.97 (0.60 to 1.66)	.90	0.99 (0.64 to 1.52)	.95	1.10 (0.69 to 1.75)	.70	0.94 (1.52 to 0.58)	.80
EQ-5D-3L score at 90 d, median (IQR)^g^^,^^h^	0.6 (−0.1 to 1.0)	0.6 (0.1 to 1.0)	0.6 (−0.1 to 1.0)	WR	1.10 (0.66 to 1.83)	.72	1.18 (0.72 to 1.94)	.52	1.06 (0.64 to 1.75)	.82	1.05 (0.60 to 1.84)	.86
Primary safety outcomes												
Death within 90 d	9 (19.6)	7 (15.2)	14 (21.5)	RR	0.91 (0.43 to 1.92)	.80	0.71 (0.31 to 1.61)	.41	0.78 (0.36 to 1.66)	.51	0.77 (0.34 to 1.79)	.55
Symptomatic intracranial hemorrhage within 24 h^i^	3 (6.5)	2 (4.3)	2 (3.1)	RR	2.12 (0.37 to 12.18)	.40	1.41 (0.21 to 9.67)	.72	NA	NA	NA	NA
Secondary safety outcomes												
Any radiologic intracranial hemorrhage within 24 h^i^	13 (28.2)	13 (28.2)	18 (27.7)	RR	1.02 (0.56 to 1.87)	.95	1.02 (0.56 to 1.87)	.95	1.03 (0.55 to 1.90)	.94	1.19 (0.64 to 2.21)	.58
Systemic bleeding^j^^,^^k^												
Mild	7 (15.2)	9 (19.6)	12 (18.5)	NA	NA	.89	NA	.87	NA	NA	NA	NA
Moderate	1 (2.2)	0 (0.0)	1 (1.5)	NA	NA		NA		NA	NA	NA	NA
Severe	17 (37.0)	14 (30.4)	20 (30.8)	NA	NA		NA		NA	NA	NA	NA

Abbreviations: EQ-5D-3L, European Quality of Life 5-Dimension 3-Level scale; eTICI, Extended Treatment in Cerebral Infarction; GenOR, generalized odds ratio; mRS, modified Rankin scale; NA, not available; NIHSS, National Institutes of Health Stroke Scale; RR, risk ratio; WR, win ratio.

^a^ Adjusted values were adjusted for age, baseline NIHSS score, baseline Alberta Stroke Program Early CT Score, occlusion site, and time from last known well to randomization. The GenOR and WR were adjusted using the inverse probability treatment weighting method.

^b^ RR was calculated using the modified Poisson regression model.

^c^ The mRS of functional disability ranges from 0 (no symptoms) to 6 (death).

^d^ GenOR was calculated by the number of wins in the dose group over the control group divided by the number of wins in the control group over the dose group among all possible pairs of mRS scores, taking 1 patient from the dose group and 1 patient from the control group. For the analysis of mRS at 90 days, the GenOR value >1 indicated a favorable shift in mRS score in the dose group relative to the control group.

^e^ The improvement angiographic reperfusion was defined as change of pre–intra-arterial tenecteplase eTICI 2b to eTICI 2c or eTICI 3, or pre–eTICI 2c to eTICI 3 after intra-arterial tenecteplase therapy. Statistical analysis was not applicable due to an insufficient number of patients with improved angiographic reperfusion.

^f^ Scores on the NIHSS range from 0 to 42, with higher values reflecting more severe neurologic impairment.

^g^ WR was calculated by the number of wins in the dose group over the control group in outcome divided by the number of wins in the control group over the dose group among all possible pairs, taking 1 patient from the dose group and 1 patient from the control group.

^h^ EQ-5D-3L is a continuous scale measure of self-reported quality of life. Scores range from −0.149 to 1, with lower scores indicating a worse quality of life.

^i^ Symptomatic intracranial hemorrhage was defined according to the European Cooperative Acute Stroke Study III standard.

^j^ Bleeding events were defined according to the Global Utilization of Streptokinase and Tissue Plasminogen Activator for Occluded Coronary Arteries criteria as follows: severe bleeding was defined as fatal or intracranial hemorrhage or other hemorrhage causing hemodynamic compromise that required blood or fluid replacement, inotropic support, or surgical intervention; moderate bleeding as bleeding that required transfusion of blood but did not lead to hemodynamic compromise requiring intervention; and mild bleeding as bleeding not requiring transfusion and not causing hemodynamic compromise (eg, subcutaneous bleeding, mild hematomas, and oozing from puncture sites).

^k^ χ^2^ Test.

## Discussion

The DATE study demonstrated that intra-arterial infusion of tenecteplase at doses of 0.0313 mg/kg or 0.0625 mg/kg, when used as an adjunct to successful reperfusion in patients with anterior circulation LVO, has an adequate safety profile to advance to further testing, whereas the next higher dose of 0.1250 mg/kg exhibited safety concerns due to a high rate of sICH. The 2 doses with an adequate safety profile did not show a statistically significant improvement in disability at 90 days, but the sample size was small, and the trial was not designed to demonstrate efficacy. The study offers valuable insights into optimizing the dosing of adjunctive intra-arterial tenecteplase for future, larger trials.

The phase 1b findings provided a nuanced understanding of the dose-dependent safety profile of intra-arterial tenecteplase. Specifically, the tenecteplase dose, 0.1250 mg/kg, was associated with an increased risk of sICH, highlighting the delicate balance between achieving effective thrombolysis and avoiding adverse bleeding events. In contrast, the lower doses of 0.0313 mg/kg and 0.0625 mg/kg did not significantly increase bleeding risk, suggesting their potential as safer alternatives. Both of these doses continued to show acceptable sICH rates in the larger phase 2a trial stage. In addition, although the results did not reach statistical significance compared with the control group, the observed numerical trend toward efficacy suggests a potential therapeutic benefit that warrants further investigation. Continued research is crucial to fully elucidate the clinical benefits of intra-arterial tenecteplase in optimizing outcomes after EVT. Importantly, the results do not exclude the possibility of a clinically meaningful benefit from intra-arterial tenecteplase. As the dose tiers 0.0313 mg/kg and 0.0625 mg/kg were statistically comparable in phase 2a, both are candidates to advance to a larger phase 2b or phase 3 trial.

Current trials, such as Adjunctive Intra-Arterial Tenecteplase Following Near-Complete to Complete Reperfusion for Large Vessel Occlusion Stroke (POST-TNK),^24^ Intra-Arterial Tenecteplase After Successful Endovascular Therapy (ANGEL-TNK),^25^ and Adjunctive Intra-Arterial Tenecteplase Following Mechanical Thrombectomy (ALLY),^26^ explored various dosing regimens of intra-arterial tenecteplase, specifically 0.0625 mg/kg, 0.1250 mg/kg, and 1.5 mg to 4.5 mg, respectively. These doses were selected based on established fractions of the intravenous alteplase dose used in previous studies like the CHOICE trial, or through recommendations from trial steering committees, rather than through systematic dose-escalation studies. The insights gained from the DATE study accordingly provide important data for empirically defining the optimal dose to enhance patient safety and patient outcomes and refine stroke treatment protocols. This systematic approach may ultimately contribute to a more effective management of acute ischemic stroke in the context of EVT.

The advent of studies of dosing strategies for intra-arterial lytic agents after successful EVT highlights a gap in our pharmacologic understanding of optimal dosing for intra-arterial administration. Unlike intravenous thrombolytic administration of drug, where dosing has been extensively studied and standardized, intra-arterial administration requires careful consideration of distinct pharmacokinetics, local drug concentration at the site of occlusion, and patient-specific factors. The intricacies of intra-arterial drug delivery may necessitate a more tailored approach to determine the most effective and safe dose.

### Limitations

This trial has several limitations. First, the phase 1b design was a nonrandomized study, and the phase 2a study was conducted in an open-label manner. However, the outcomes were assessed by clinicians who were blinded to the treatment assignments. Second, the trial was not powered to achieve definitive conclusions regarding the efficacy of adjunctive intra-arterial tenecteplase after successful EVT in improving outcomes for patients with anterior circulation LVO ischemic stroke but to identify adequately safe doses for further testing. Future larger sample size trials to further evaluate the safety and efficacy of adjunctive intra-arterial tenecteplase after successful EVT are necessary. Third, although patients in this study were randomized with stratification based on age and admission NIHSS score, there was a slight imbalance in occlusion sites between groups. To mitigate potential bias, adjusted analyses were conducted. Fourth, perfusion studies were not required to be performed to better characterize tissue-level penumbra and core status. This approach increases the pragmatic applicability of trial results but reduces explanatory insight into response mechanisms. Fifth, the trial was conducted in Asian patients with anterior LVO and small ischemic core; the findings may not be generalizable to other populations.

## Conclusions

These phase 1 and 2 randomized clinical trials found that administering tenecteplase at doses of 0.0313 mg/kg or 0.0625 mg/kg after successful EVT within 24 hours of onset in patients with anterior circulation LVO acute ischemic stroke is adequately safe to advance to larger trials. However, due to the study’s limitations, particularly in size and scope, the efficacy of these doses could not be conclusively established. Therefore, further validation through a larger phase 2b or phase 3 studies is necessary to assess the potential therapeutic benefits and to refine the optimal dosing strategy for improving clinical outcomes in this patient population.

## Notes

Supplement 1.
Trial Protocol.

Supplement 2.
Statistical Analysis Plan.

Supplement 3. **eAppendix.** List of Investigators **eMethods 1.** Study Design of Phase 1b Dose Escalation Trial **eMethods 2.** Detailed Inclusion and Exclusion Criteria **eMethods 3.** Definition of Analyzed Population **eMethods 4.** Characteristics of EQ-5D-3L Value Sets in China **eTable 1.** Characteristics of the Phase 1b Population by Group **eTable 2.** Characteristics of the Pooled Analysis **eTable 3.** Pooled Analysis of Study Outcomes **eTable 4.** Summary of Serious Adverse Events Reported by Local Investigators of the Phase 1b (Safety Population) **eTable 5.** Summary of Adverse Events Reported by Local Investigators of the Phase 1b (Safety Population) **eTable 6.** Summary of Serious Adverse Events Reported by Local Investigators of the Phase 2a (Safety Population) **eTable 7.** Summary of Adverse Events Reported by Local Investigators of the Phase 2a (Safety Population) **eFigure 1.** Distribution of 30 Enrollment Center in China **eFigure 2.** Distribution of the Modified Rankin Scale Score at 90 Days (Phase 1b) **eFigure 3.** Distribution of the Modified Rankin Scale Score at 90 Days (Phase 1b+2a)

Supplement 4.
Data Sharing Statement.
