# Supplementary material for: Intra-Arterial Tenecteplase After Successful Reperfusion in Large Vessel Occlusion Stroke: A Randomized Clinical Trial
Source: JAMA Neurol. 2025 Jul 5;82(9):895–904. doi: 10.1001/jamaneurol.2025.2036 (PMC12228979; doi:10.1001/jamaneurol.2025.2036)
Supplement: Supplement 1. — Trial Protocol. [file jamaneurol-e252036-s001.pdf]

Safety and Efficacy of Adjunctive Intra-arterial Tenecteplase following Successful Thrombectomy in Patients with Large Vessel Occlusion: A Phase 1/2 Randomized Clinical Trial

(DATE)

A multicenter, prospective, randomized, open-labeled, blinded outcome Phase Ib/IIa clinical trial

Principal Investigator: Prof Zhenhua Zhou, MD; Prof Wenjie Zi, MD

Sponsor: The First Affiliated Hospital, Army Medical University; The Second Affiliated Hospital, Army Medical University

Funded by: The Key Project of Chongqing Science Health Joint Medical Research Project, National Natural Science Foundation of China, the Major Project of Clinical Research Incubation at the First Affiliated Hospital of Army Medical University, Key Special Projects for Technological Innovation and Application Development in Chongqing and Chongqing Postdoctoral Program for Innovative Talent

Version Number:1.0

1 June 2023

Table of contents

List of Abbreviations......1
1. Protocol Summary......3
1.1 Organization Structure......3
1.2 Study Synopsis......4
DATE I Synopsis......4
DATE II Synopsis......7
1.3 Flow Chart......10
1.4 Schedule of Assessments......11
2. Introduction and Background Information......12
2.1 Stroke......12
2.2 Current reperfusion therapy for acute ischemic stroke......13
3. Study Design......15
3.1 Objectives and Endpoints......15
3.1.1 Objectives......15
3.1.2 The primary endpoint......15
3.1.3 The secondary end-point......16
3.2 Overall design......17
3.3 Enrollment Inclusion and Exclusion Criteria......19
3.3.1 Inclusion Criteria......19
3.3.2 Exclusion Criteria......19
3.4 Enrollment and Randomization......20
3.4.1 Treatment blinding......20
3.4.2 Enrollment phase Ib......20
3.4.3 Enrollment and randomization phase IIa......20
3.5 Study Interventions......20
3.6 Assessments and Procedures conducted during the study......21
3.7 Study Discontinuation criteria......23
3.7.1 Site Discontinuation......23
3.7.2 Participant Discontinuation......24
3.7.3 Lost to Follow-up......24
3.8 Adverse Events and Serious Adverse Events......25
3.8.1 Adverse Events Reporting......25

3.8.2 Recording of adverse events ... 26

4. Statistical Methods ... 26

4.1 General remarks ... 27

4.2 Sample size ... 27

4.2.1 Phase Ib ... 27

4.2.2 Phase IIa ... 27

4.3 Population sets ... 28

4.4 Statistical analyses ... 28

4.4.1 Primary Endpoint Analysis ... 28

4.4.2 Secondary Endpoints Analysis ... 29

4.4.3 Handling of missing data ... 29

4.5 Handling of dropouts and missing or invalid data ... 30

5. Data handling ... 30

5.1 Completion of the electronic CRFs (eCRF) ... 30

5.2 Data management ... 31

5.3 SAE reconciliation ... 31

5.4 Database lock ... 31

5.5 Independent Committees ... 31

6. Quality control / monitoring ... 32

7. Ethics and regulatory aspects ... 33

7.1 Ethical considerations ... 33

7.2 Ethics Committee ... 33

7.3 Informed Consent ... 33

8. Finance and Insurance ... 33

8.1 Finance ... 33

8.2 Insurance ... 33

9. Study report and publications ... 34

10. References ... 35

Appendix table 1 Modified Rankin Scale ... 3 7

Appendix table 2 Extended Treatment In Cerebral Ischemia (eTICI) Scale ... 38

Appendix table 3 NIH Stroke Scale ... 39

Appendix table 4 EUROQOL 5D-3L ... 43

List of Abbreviations

AE	Adverse Event
AHA/ASA	American Heart Association/American Stroke Association AMI Acute Myocardial Infarction
ASPECTS	Alberta Stroke Program Early CT score
CRF	Case Report Form
CT	Computerized Axial Tomography
CTA	Computerized Axial Tomography Angiography
CTP	Computerized Tomography Perfusion
DLT	Dose Limit Toxicity
EVT	Endovascular Thrombectomy
EQ5D-3L	EuroQol-5 dimensions-3 level
eTICI	expanded Treatment In Cerebral Infarction
GCP	Good Clinical Practice
IA	Intra-arterial
ICA	Internal Carotid Artery
ICH	Intra-cerebral Hemorrhage
IV	Intravenous
IVT	Intravenous Thrombolysis
LVO	Large Vessel Occlusion
MAR	Missing at random
MCA	Middle Cerebral Artery
mFAS	Modified Full Analysis Set
MRA	Magnetic Resonance Angiography
MRI	Magnetic Resonance Imaging
mRS	Modified Rankin Scale
MT	Mechanical thrombectomy
MTD	Maximum Tolerated Dose
mTICI	Modified Treatment In Cerebral Infarction scale
NIHSS	National Institute of Health Stroke Scale
NCCT	Non Contrast Computer Tomography
OR	Odds Ratio
PP	Per Protocol
PROBE	Prospective randomized open blinded end-point
RACE	Rapid Arterial Occlusion Evaluation
RCT	Randomized controlled trial
RD	Royal Decree
rt-PA	Recombinant tissue Plasminogen Activator

SAE	Serious Adverse Event
SICH	Symptomatic Intracranial Hemorrhage

1. Protocol Summary

1.1 Organization Structure

Steering Committee

Chairman: Prof Zhenhua Zhou, The First Affiliated Hospital to the Army Medical University

Members:

Data Safety Monitoring Board

Chairman:

Members:

Imaging Assessment Committee

Chairman:

Members:

Adverse Event Adjudication Committee

Chairman:

Members:

Outcome Committee

Chairman:

Members:

1.2 Study Synopsis

DATE I Synopsis

Public title	A pilot dose-escalation safety study of adjunctive intra-arterial tenecteplase after successful endovascular thrombectomy in patients with large vessel occlusion stroke
Study design	A single arm, prospective, multicenter, exploratory, dose-escalation, open label, non-randomized Phase Ib trial
Outcomes	
Primary outcome	1. Symptomatic Intracranial Hemorrhage (sICH) within 24 hours
Secondary outcomes	1. Proportion of patients with excellent outcome (mRS score 0 to 1) at 90 days;<br>2. Proportion of patients with functional independence (mRS score 0 to 2) at 90 days;<br>3. Level of disability (ordinal distribution of mRS scores) at 90 days;<br>4. Favorable shift in reperfusion on the eTICI score after intra-arterial tenecteplase thrombolysis therapy. All post-EVT, pre-IA and final post-IA final, angiograms will be scored at a core lab by central and blinded reviewers using the eTICI and classified as eTICI2b, eTICI2c, and eTICI3. The post treatment angiographies will be scored using the eTICI and classified as "improved"， "worsened" or "unchanged" with regard to the pre-IA infusion eTICI score;<br>5. The change from baseline of the National Institutes of Health Stroke Scale (NIHSS) score to 5–7 days or discharge if earlier;<br>6. Quality of life measured with the EuroQol Group 5-Dimension Self-Report Questionnaire (EQ-5D-3L) at 90 days;<br>7. Mortality within 90 days.
Inclusion criteria	1. Age ≥ 18 years;<br>2. Time from last known well within 24 hours;<br>3. Large vessel occlusive stroke in the anterior circulation confirmed by computed tomography angiography/magnetic resonance angiography, including intracranial segment of the internal carotid artery, first or second segment of the middle

	cerebral artery;<br>4. Alberta Stroke Program Early CT Score (ASPECTS) ≥ 6 based on non-contrast computed tomography;<br>5. NIHSS score ≥ 6;<br>6. Successful endovascular thrombectomy (the expanded Thrombolysis in Cerebral Infarction [eTICI] grade of 2b or higher);<br>7. Total pass numbers of thrombectomy procedure ≤3;<br>8. Written informed consent signed by patients or their legal presents.
Exclusion criteria	1. NIHSS score ≥ 25;<br>2. Intracranial hemorrhage confirmed by cranial computed tomography (CT) or magnetic resonance imaging (MRI);<br>3. Treated by intravenous thrombolysis;<br>4. Pre-stroke mRS score≥ 2;<br>5. Intraoperative digital subtraction angiography suggesting vessel penetration, dissection, or extravasation of contrast medium;<br>6. Pregnant or lactating patients;<br>7. Allergic to contrast agents or tenecteplase;<br>8. Systolic pressure greater than 185 mmHg or diastolic pressure greater than 110 mmHg after aggressive treatment;<br>9. Genetic or acquired bleeding disposition with anticoagulation factor deficiency or already taking oral anticoagulants within 48 hours and INR > 1.7;<br>10. Blood glucose < 2.8 mmol/L (50 mg/dl) or > 22.2 mmol/L (400 mg/dl), platelets < 90*10^9/L;<br>11. History of bleeding in the last one month (gastrointestinal and urinary tract bleeding);<br>12. Patients on chronic hemodialysis and severe renal insufficiency (glomerular filtration rate < 30 ml/min or blood creatinine > 220μmol/L [2.5mg/dl]);<br>13. Any terminal illness with a life expectancy of less than 6 months;<br>14. Intracranial aneurysm, arteriovenous malformation;<br>15. Brain tumors with occupying effect on imaging;

	16. Puncture to recanalization time > 90 min;<br>17. Current participation in another clinical trial;<br>18. Unlikely to be available for 90-day follow-up.
Interventions	Intra-arterial tenecteplase thrombolysis through a distal access catheter or microcatheter positioned proximal to the initially in occluded arteries after successful endovascular treatment. Patients will be treated in 4 escalating dosage level: 0.03125 mg/kg (1/8 i.v dose), 0.0625 mg/kg (1/4 i.v dose),0.125 mg/kg (1/2 i.v dose), and 0.1875 mg/kg (3/4 i.v dose).
Sample size	The Endovascular Treatment for Acute Anterior Circulation Ischemic Stroke registry in China showed that the incidence of sICH within 24 hours after EVT was 13.8%.[1] Based on this data, we have designed a 14+8 enrollment plan as follows:<br>(1) 14 subjects were included in the trial for the first time for each dose;<br>(2) If<2 dose limit toxicity (DLT, defined as the sICH occurrence within 24 hours after EVT), the trial advances to the next dose level;<br>(3) If 2/14 DLT, then include 8 more people at the same dose;<br>(4) If 2/14+0-1/8DLT (9.1% to 13.6%), proceed to the next dose level;<br>(5) If 2/14+2/8 DLT (18.2%) or 3/14 DLT, then this dose is deemed unsafe and the prior lower dose is the MTD.

DATE II Synopsis

Public title	Safety and efficacy pilot study of adjunctive intra-arterial tenecteplase after successful endovascular thrombectomy in patients with large vessel occlusion stroke
Study design	A prospective, multicenter, randomized controlled, open-label, endpoint blind Phase II trial
Outcomes	
Primary outcomes	1. The proportion of patients with a excellent outcome (mRS score of 0 or 1) at 90 (±14) days
Secondary outcomes	1. Proportion of patients with functional independence (mRS score 0 to 2) at 90 days;<br>2. Level of disability (ordinal distribution of mRS scores) at 90 days;<br>3. Favorable shift in eTICI score after intra-arterial tenecteplase thrombolysis therapy;<br>4. The change of the NIHSS score from baseline to 5–7 days or discharge if earlier;<br>5. EQ-5D-3L at 90 days;<br>6. sICH rate within 24 hours;<br>7. Mortality within 90 days.
Inclusion criteria	1. Age ≥18 years;<br>2. Time from onset to randomization within 24 hours;<br>3. Large vessel occlusive stroke in the anterior circulation confirmed by computed tomography angiography/magnetic resonance angiography, including intracranial segment of the internal carotid artery, first or second segment of the middle cerebral artery;<br>4. ASPECTS score ≥ 6 based on NCCT;<br>5. NIHSS score ≥ 6;<br>6. Successful endovascular thrombectomy (eTICI 2b-3);<br>7. Total pass numbers of thrombectomy procedure ≤ 3;<br>8. Written informed consent signed by patients or their legal presents.
Exclusion criteria	1. NIHSS score ≥ 25;<br>2. Intracranial hemorrhage confirmed by cranial computed tomography (CT) or magnetic resonance imaging (MRI);

	3. Treated by intravenous thrombolysis;<br>4. Pre-stroke mRS score ≥ 2;<br>5. Intraoperative DSA angiography suggests vessel penetration, dissection, or extravasation of contrast medium;<br>6. Pregnant or lactating patients;<br>7. Allergic to contrast agents or tenecteplase;<br>8. Systolic pressure greater than 185 mmHg or diastolic pressure greater than 110 mmHg after aggressive treatment;<br>9. Genetic or acquired bleeding disposition with anticoagulation factor deficiency or already taking oral anticoagulants within 48 hours and INR > 1.7;<br>10. Blood glucose < 2.8 mmol/L (50 mg/dl) or > 22.2 mmol/L (400 mg/dl), platelets < 90*10^9/L;<br>11. History of bleeding in the last 1 month (gastrointestinal and urinary tract bleeding);<br>12. Patients on chronic hemodialysis and severe renal insufficiency (glomerular filtration rate < 30 ml/min or blood creatinine > 220 μmol/L (2.5mg/dl));<br>13. Any terminal illness with a life expectancy of less than 6 months;<br>14. Intracranial aneurysm, arteriovenous malformation;<br>15. Brain tumors with occupying effect on imaging;<br>16. Puncture to recanalization time > 90 min;<br>17. Current participation in another clinical trial;<br>18. Unlikely to be available for 90-day follow-up.
Interventions	Three arms were designed and patients were randomized to receive 1 of the 2 selected doses (dose A or dose B) of tenecteplase or control in a 1:1:√2 ratio according to the Phase Ib DATE trial, which in turn yields probabilities of assignment of 0.293, 0.293, and 0.414, respectively. Patients were randomly assigned by using a real-time internet-based system. This process was automated from study startup, which allowed for complete concealment of the sequence of allocation.

Sample size	Phase IIa is an exploratory trial with plans to randomize 46 subjects each into dose group A and dose group B, and 65 subjects into the control group in a 1:1: √ 2 ratio. This approach is similar to the sample size calculation method used in the APRIL study.
Statistical Analysis	A statistical analysis plan (SAP) including the list of all tables, listings, and graphs will be issued before the study database lock. The analysis will assume a progressive (monotonicity) relationship (i.e., any dose above an unsafe dose will also be considered unsafe). Categorical variables will be summarized with counts and percentages. Continuous variables will be summarized with the median, the inter-quartile range, the minimum, and the maximum. Patient's compliance with eligibility criteria and treatment administration, major protocol non-compliances, patient's withdrawals and the reason for withdrawal (e.g., AE, protocol non-compliance, lost to follow up, failed to return, consent withdrawal, and other reasons) and assignment to each analysis population will be summarized by means of the appropriate statistics.<br>The main results will be provided with 95% confidence intervals.<br>The primary effect variable in both phase Ib and IIa will be analyzed using generalized linear models from which risk ratio (RR) with its 95% CI will be estimated. The secondary outcomes and safety outcomes will be analyzed using generalized linear models or win ratio. A prespecified supportive pool analysis that combining the phase Ib and phase IIa will be performed. The primary data analyses will adhere to the intention-to-treat principle. Additionally, per-protocol analyses will be conducted as supplementary analyses. For all statistical analyses, SAS version 9.4 (SAS Institute) and R version 4.4.0 or higher version (R Foundation for Statistical Computing) will be utilized. The reporting of trial results will conform to the Consolidated Standards of Reporting Trials guidelines for randomized trials.

1.3 Flow Chart

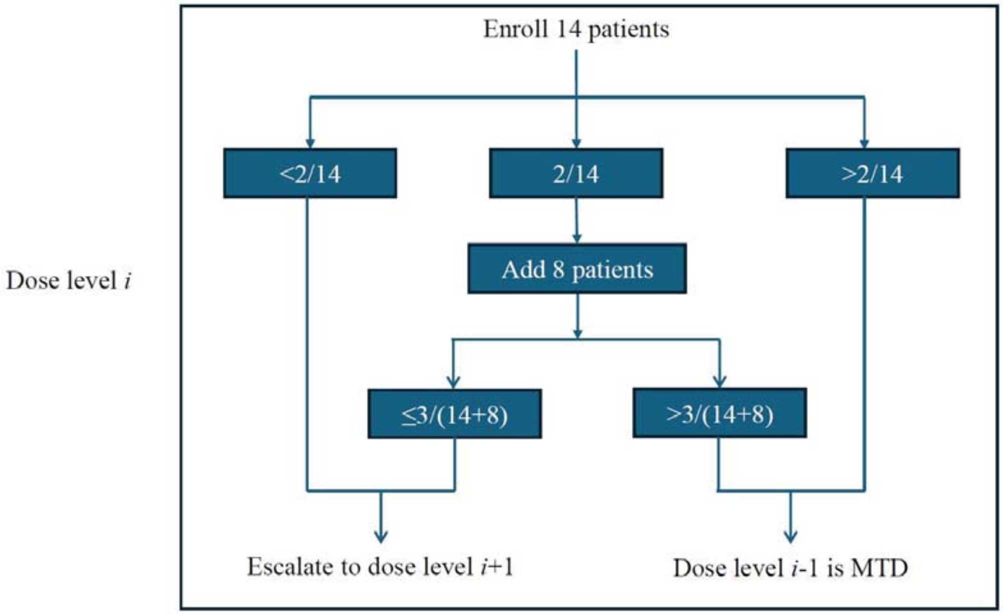

Figure 1. Flow chart of the DATE Phase Ib trial.

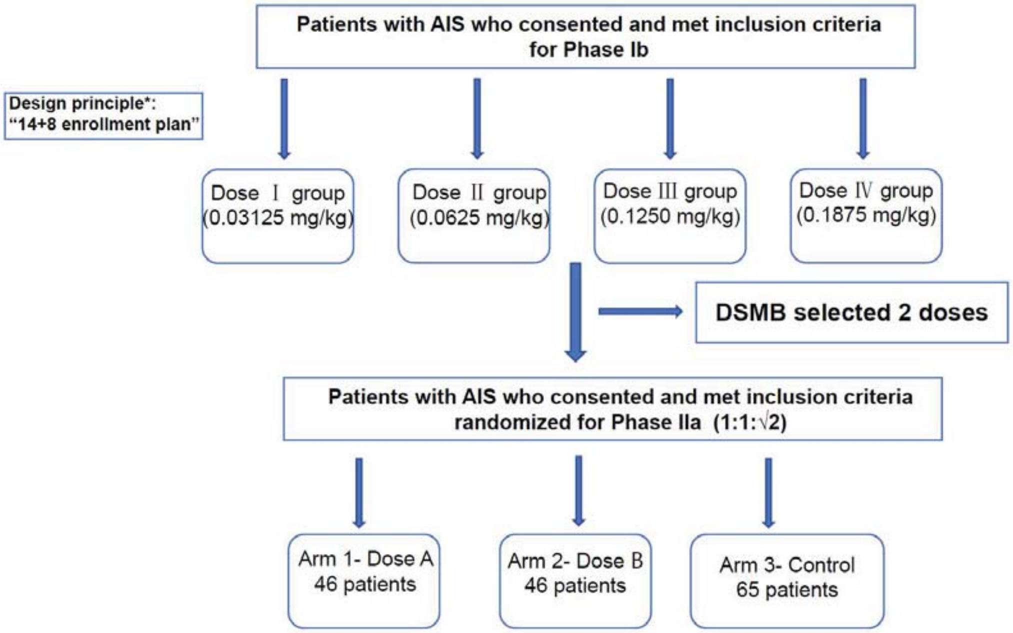

Figure 2. Flow chart of the DATE trial.

1.4 Schedule of Assessments

**Assessments**	**Baseline information**	**Procedure/ Allocation**	**Follow-up 24h (-/+12h)**	**Follow-Up 5 -7days**	**Follow-up 90 days (±14 d)**
Admission Details	X				
Demographics	X				
Medical History	X				
Eligibility Criteria		X			
Informed Consent	X				
Randomization (Phase IIa)		X			
Local laboratory result	X				
mRS	X				X
NIHSS assessment	X			X	
NCCT/MRI	X		X		
Angiogram		X			
medication administration		X			
Post-MT angiography		X			
Procedure Details		X			
EuroQolEQ-5D					X
(S) AEs*	⋯on an ongoing basis⋯				
Relevant Meds	X	X	X	X	X

Remarks:

1.The NIHSS, mRS, and eTICI scoring scales are detailed in the Supplementary Table.

2. *: Adverse events (AEs) are any adverse medical events, including the deterioration of a patient's condition who has already received medication, and do not necessarily have a clear causal relationship with the treatment. When the following situations occur, the adverse event will be defined as a serious adverse event (SAE): Leading to death; Immediately endangering life; Causing persistent and obvious disability or disability; Resulting in prolonged hospitalization of patients; Congenital malformations or birth defects; Based on appropriate medical judgment, this adverse event can put patients at risk and may require medical/surgical intervention to prevent more serious events from occurring.

2. Introduction and Background Information

2.1 Stroke

Globally, stroke ranks as the leading cause of both disability and mortality, presenting a formidable challenge to individuals and society as a whole.[2] Since 2015, stroke has become the primary cause of death and disability in China, posing a significant threat to public health as a major chronic non-communicable disease.[3] As the largest developing nation, China has a population that accounts for one-fifth of the global total, with its number of stroke patients being the highest in the world. The 2019 Global Burden of Disease (GBD) Study revealed alarming statistics: globally, there were 12.20 million new stroke cases, 101 million prevalent stroke cases, and 6.55 million stroke-related deaths.[4] In China specifically, there were 3.94 million new cases, 28.76 million prevalent cases, and 2.19 million deaths.[5]

The vast majority of stroke cases can be classified into two distinct types: hemorrhagic strokes and ischemic strokes. Hemorrhagic strokes occur when a blood vessel ruptures within the brain, while ischemic strokes are caused by a blockage in a brain artery. Both conditions lead to local hypoxia, resulting in brain tissue damage. Despite the severity and prevalence of both types, ischemic strokes are more common, accounting for 85% of all stroke cases.[6] Typically, the blockages in ischemic strokes are caused by blood clots that become lodged in one of the brain's arteries.

Despite the sluggish progress in treating hemorrhagic stroke, significant advancements have been achieved in managing acute ischemic stroke over the past decades. Intravenous thrombolysis and, more recently, endovascular thrombectomy (EVT) for large vessel occlusion (LVO) have revolutionized our capacity to mitigate long-term disability among eligible patients. The widespread establishment of specialist stroke units, coupled with the substantial evidence supporting the efficacy of intravenous thrombolysis and the groundbreaking randomized controlled trials validating the benefits of EVT, have marked a notable milestone.[7]

However, despite these advancements, acute ischemic stroke remains the leading cause of permanent disability among adults. This underscores the urgent necessity to

relentlessly explore and develop novel therapeutic approaches for this devastating condition. The pursuit of innovative treatments holds the key to further improving outcomes and reducing the burden of stroke on individuals and societies worldwide.

2.2 Current reperfusion therapy for acute ischemic stroke

Reperfusion therapy, the first-line treatment for acute ischemic stroke (AIS), currently includes two widely accessible treatments. The initial approach involves thrombolysis with tissue Plasminogen Activator (tPA), with alteplase being the most commonly used variant. TPA replaced the earlier thrombolytic agent streptokinase, and its effectiveness in acute ischemic stroke was first demonstrated in a 1995 study by the National Institute of Neurological Disorders and Stroke (NINDS).[8,9] Subsequent studies have confirmed the efficacy of tPA up to 4.5 hours after the onset of ischemic stroke symptoms.[10] However, tPA has its drawbacks. It must be administered within 4.5 hours of stroke onset, which is challenging in many situations. Moreover, it can cause severe side effects, the most common being hemorrhage, sometimes severe enough to be life-threatening.[8] The risk of hemorrhage increases with delayed administration of tPA. Fortunately, the rate of symptomatic hemorrhage is less than 2% if alteplase is administered within the 4.5-hour window.[11] These limitations have prompted the search for alternative thrombolytics, with tenecteplase emerging as a promising candidate. Tenecteplase offers a longer half-life, higher specificity, and a reduced risk of hemorrhagic transformation (HT) compared to tPA.[12] Additionally, previous studies have shown that intravenous tenecteplase is associated with better reperfusion and functional outcome than alteplase in patients with acute ischemic stroke (AIS).[13] Another widely available treatment is EVT, which gained acceptanc e in 2015 following the publication of five clinical trialsdemonstrating its efficacy wi thin 6-8 hours of ischemic stroke onset.[14] More recentstudies have shown that EVT remains effective up to 24 hours after onset.[15]

2.3 Adjunctive intra-arterial thrombolysis targeting the "no-reflow" phenomenon in LVO stroke

Traditionally, large vessel occlusions have been the focus of all EVT and many intravenous thrombolytic studies. Besides primary thrombus removal, macrovascular reperfusion strategies can also address smaller distal emboli, often created as a secondary complication of EVT. These thromboembolic events can affect new vascular territories and are not always visible or accessible for mechanical removal during angiography.[16,17] Emerging data suggest that clearing these smaller thrombi and achieving complete reperfusion, even hours after EVT, is associated with favorable clinical outcomes, similar to those achieved by patients who experience complete reperfusion following EVT.[18]

The intricate network of cerebral microvascular, typically invisible on angiograms, could be a crucial focus for reperfusion efforts. Studies in both cardiac and cerebral preclinical models have introduced the notion of "no-reflow" in microvasculature following the reopening of larger vessels.[19] The precise mechanisms behind this microvascular no-reflow are not fully comprehended, although they might involve obstructions by fibrin, platelets, or leukocytes within the vessels, thickening of capillary endothelium, or pericyte contractions.[20] In clinical practice, perfusion imaging has been employed to detect the no-reflow phenomenon among patients experiencing acute ischemic strokes. The Effect of Intra-arterial Alteplase vs Placebo Following Successful Thrombectomy on Functional Outcomes in Patients with Large Vessel Occlusion Acute Ischemic Stroke (CHOICE) trial, which compared intra-arterial alteplase with a placebo in patients following successful EVT, underscored the significance of the cerebral microcirculation as a potential therapeutic target.[21] This trial involved patients with large vessel occlusions in the anterior circulation who had successful macrovascular recanalization (graded eTICI 2b-3) and were then randomized to receive either intra-arterial alteplase or a placebo. The findings indicated that intra-arterial alteplase was associated with better outcomes at the 90 days. However, the study's conclusions are somewhat constrained by its small sample size due to prematurely termination. While the findings await confirmation in larger studies, the CHOICE trial nonetheless demonstrates the possible advantages of

interventions aimed at the microvascular system. Moreover, it did not conduct a dose escalation exploratory trial, leaving the ideal dose that balances efficacy and safety still unknown.

Tenecteplase, a third-generation thrombolytic drug, has demonstrated superiority over alteplase in improving excellent functional outcomes and reducing disability at 3 months.[22] There is an urgent need to determine which dose provides the best balance of efficacy and safety in patients with LVO following successful EVT. Current trials such as POST-TNK[23] and ANGEL-TNK[24] are exploring different doses of intra-arterial tenecteplase in patients with acute ischemic stroke due to LVO following successful EVT, using doses of 0.0625 mg/kg and 0.125 mg/kg without escalation exploratory trial, respectively. Therefore, this study will provide new insights to explore the optimal dose of adjunctive intra-arterial tenectepalse administration in these patients.

3. Study Design

3.1 Objectives and outcomes

3.1.1 Objectives

The primary objective of this study is to preliminarily assess the safety and efficacy of adjunctive intra-arterial tenecteplase at different doses following successful EVT in patients with acute ischemic stroke due to LVO to determine the promising dose to advance to a pivotal trial. This study will take the form of a Phase Ib/IIa trial. Initially, doses that meet safety criteria established during the Phase Ib will be administered during the Phase IIa.

3.1.2 The primary outcome

Dose Escalation (Phase Ib):

The primary outcome is the proportion of symptomatic intracranial hemorrhage (sICH) within 24 hours, according to the European Cooperative Acute Stroke Study (ECASS) III.

The European Cooperative Acute Stroke Study (ECASS) III definition of symptomatic intracranial hemorrhage (sICH) was any hemorrhage with neurologic deterioration, as indicated by an National Institutes of Stroke Scale (NIHSS) score that was higher by 4 or more points than the value at baseline or the lowest value in the first 7 days, or that led to death or was identified as the predominant cause of the neurological deterioration.

The ECASS classification for hemorrhage types includes:

(1) Hemorrhagic infarction (HI):

HI1: small petechiae along the margins of the infarct.

HI2: more confluent petechiae without mass effect.

(2) Parenchymal hematoma (PH):

PH1: blood clots in ≤ 30% of the infarcted area with some mass effect.

PH2: blood clots in >30% of the infarcted area with significant mass effect.

<u>Dose Expansion (Phase IIa):</u>

The primary outcome will be the proportion of patients with a excellent outcome (mRS score of 0 or 1) at 90 (±14) days. Primary outcome assessments will be performed at 90 (±14) days by two independent, certified physicians who are blinded to the treatment details. To maintain the reliability, accessibility, and traceability of the mRS score, we will retain a video or audio records of the 90-day follow-up for all patients. If video or audio recordings are unavailable, outcomes will be determined in-person by certified local investigators, who will be also unaware of the treatment assignment.

3.1.3 The secondary outcomes

<u>Dose Escalation (Phase Ib):</u>

1. Proportion of patients with excellent outcome (mRS score 0 to 1) at 90 days;
2. Proportion of patients with functional independence (mRS score 0 to 2) at 90 days;
3. Level of disability (ordinal distribution of mRS scores) at 90 days;

4. Favorable shift in reperfusion on the eTICI score after intra-arterial tenecteplase thrombolysis therapy. All post-EVT, pre-IA and final post-IA final, angiograms will be scored at a core lab by central and blinded reviewers using the eTICI and classified as eTICI2b, eTICI2c, and eTICI3. The post treatment angiographies will be scored using the eTICI and classified as "improved"，“worsened” or "unchanged" with regard to the pre-IA infusion eTICI score.

5. The change from baseline of the NIHSS score to 5–7 days or discharge if earlier;

6. EQ-5D-3L at 90 days;

7. Mortality within 90 days.

<u>Dose Expansion (Phase IIa):</u>

1. Proportion of patients with functional independence (mRS score 0 to 2) at 90 days;
2. Level of disability (ordinal distribution of mRS scores) at 90 days;
3. Favorable shift in eTICI score after intra-arterial tenecteplase thrombolysis therapy;
4. The change of the NIHSS score from baseline to 5–7 days or discharge if earlier;
5. EQ-5D-3L at 90 days;
6. sICH rate within 24 hours;
7. Mortality within 90 days.

3.2 Overall design

This is a Phase Ib/IIa clinical trial divided in two parts: the first part (Phase Ib) is a pilot dose-escalation safety study, and the second one (Phase IIa) is an exploratory study to assess safety and efficacy of adjunctive intra-arterial tenecteplase after successful EVT in patients with LVO stroke at two different selected doses.

<u>Dose Escalation (Phase Ib):</u>

Phase Ib is designed to (I) assess the dose-related safety and potential efficacy of adjunctive intra-arterial tenecteplase after successful EVT in patients with LVO stroke, and (II) identify the recommended phase 2 dose (RP2D).

During this phase, participants received intra-arterial administrations of different doses of tenecteplase following successful EVT. Patients will be treated in 4 escalating dosage level: 0.03125 mg/kg (1/8 i.v dose), 0.0625 mg/kg (1/4 i.v dose),0.125 mg/kg (1/2 i.v dose), and 0.1875 mg/kg (3/4 i.v dose).

According to the Chinese Acute Anterior Circulatory Ischemic Stroke Endovascular Treatment Registry Study, 13.8% of patients experienced sICH within 24 hours of receiving EVT.[1] Based on this data, we designed a 14+8 enrollment plan. Using sample sizes typical for dose escalation phase I clinical trials[25,26], we will use a 14+8 design scheme with sICH occurrence within 24 hours after EVT as the dose-limiting toxicity. At each tier, 14 patients will initially be enrolled. If fewer than 2 of 14 subjects develop sICH, the trial will advance to the next tier. If 2 of the 14 develop sICH, 8 more patients will be enrolled at that dose. If 0-1 of the additional 8 patients develops sICH, the trial will advance to the next tier. If 2 of the additional 8 patients develops sICH or 3 of the first 14 develop sICH, that dose will be deemed not tolerated and the immediately preceding dose will be the estimated MTD.

Dose Expansion (Phase IIa):

Two doses (A and B) will be chosen by the DSMB and investigators jointly according to the results in Phase Ib. In Phase II, a total of 157 new patients will be enrolled, with 46 patients assigned to the dose A group, 46 patients assigned to the dose B group, and an additional 65 patients allocated to the control group.

During this phase, 3 arms will be studied and patients will be randomized with stratification allocation according to two strata: patient age (<70 vs ≥70 years), and admission NIHSS score (< 15 vs ≥15), to receive 1 of the 2 selected doses often ecteplase, or to the control group in a 1:1: √ 2 ratio, which in turn yieldspr obabilities of assignment of 0.293, 0.293, and 0.414, respectively. Patients will bera ndomly assigned by using a real-time internet-based system. This process is

automated which allows for concealment of the sequence of allocation.

3.3 Enrollment Inclusion and Exclusion Criteria

3.3.1 Inclusion Criteria

1. Age ≥18 years;
2. Time from last known well within 24 hours;
3. Large vessel occlusive stroke in the anterior circulation confirmed by CTA/MRA (Including intracranial segment of internal carotid artery and M1 or M2 segment of middle cerebral artery) and the vessel responsible for the signs and symptoms of acute ischemic stroke;
4. ASPECTS score ≥ 6 based on NCCT;
5. NIHSS score ≥ 6;
6. Successful endovascular thrombectomy (eTICI 2b-3);
7.Total pass numbers of thrombectomy procedure ≤ 3;
8. Written informed consent signed by patients or their legal presents.

3.3.2 Exclusion Criteria

1. NIHSS score ≥ 25;
2. Intracranial hemorrhage confirmed by cranial CT or MRI;
3. Treated by intravenous thrombolysis;
4. Pre-stroke mRS score ≥ 2;
5. Intraoperative DSA angiography suggests vessel penetration, dissection, or extravasation of contrast medium;
6. Pregnant or lactating patients;
7. Allergic to contrast agents or tenecteplase;
8. Systolic pressure greater than 185 mmHg or diastolic pressure greater than 110 mmHg after aggressive treatment;
9. Genetic or acquired bleeding disposition with anticoagulation factor deficiency or already taking oral anticoagulants within 48 hours and INR > 1.7;
10. Blood glucose < 2.8 mmol/L (50 mg/dl) or > 22.2 mmol/L (400 mg/dl),

platelets < 90*10^9/L;

11. History of bleeding in the last 1 month (gastrointestinal and urinary tract bleeding);

12. Patients on chronic hemodialysis and severe renal insufficiency (glomerular filtration rate < 30 ml/min or blood creatinine > 220 μmol/L (2.5mg/dl));

13. Any terminal illness with a life expectancy of less than 6 months;

14. Intracranial aneurysm, arteriovenous malformation;

15. Brain tumors with occupying effect on imaging;

16. Puncture to recanalization time > 90 min;

17. Current participation in another clinical trial;

18. Unlikely to be available for 90-day follow-up.

3.4 Randomization

The Phase Ib trial is a single arm, non-randomized clinical trial. In the Phase IIb DATE trial, 3 arms will be studied and patients will be randomized with stratification allocation according to two strata: patient age (<70 vs ≥ 70 years), and admission NIHSS score (< 15 vs ≥ 15), to receive 1 of the 2 selected doses of tenecteplase, or to the control group in a 1:1:√2 ratio, which in turn yields probabilities of assignment of 0.293, 0.293, and 0.414, respectively using a real-time internet-based system. This process was automated from study startup, which allowed for complete concealment of the sequence of allocation.

In both Phase Ib and IIa parts of the trial, it is imperative that the informed consent is signed by the participant or their designated legal representative prior to the administration of any study-related treatments.

3.5 Study Interventions

Eligible patients assigned to the tenecteplase group will undergo an infusion of intra-arterial tenecteplase with the assigned dose. This infusion will be administered through a distal access catheter or microcatheter positioned proximal to the initially

occluded artery within 10 minutes after randomization. Patients allocated to the control group will terminate the procedure without further intra-arterial thrombolysis.

All enrolled patients should be monitored in the acute stroke unit and can be admitted to the intensive care unit if necessary. All enrolled patients will undergo standardized medical treatment management and subsequent secondary preventive medication according to the Chinese Guidelines for Endovascular Treatment of Acute ischemic Stroke 2018.[27]

3.6 Assessments and Procedures conducted during the study

General Assessments and Procedures

The flow of treatment should be the following:

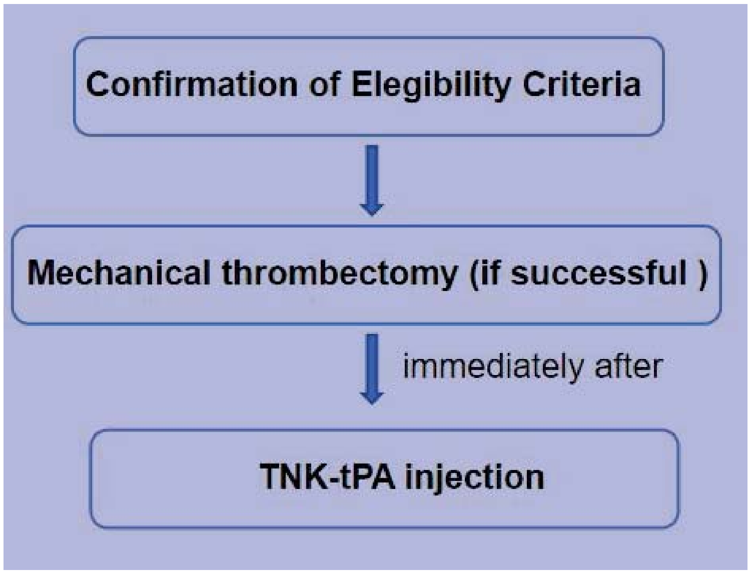

Each subject will undergo the following procedures:

- **Informed Consent Form signature**

Before initiating any procedure, participants and/or their legally authorized representatives must be provided with comprehensive oral and written information regarding the study, encompassing its objectives, design, potential risks, and anticipated benefits. If they subsequently consent to take part, signing the informed consent form (ICF) is a must. All individuals who fulfill the specified eligibility criteria are eligible for enrollment in the DATE clinical trial.

- **Demographics and Physical examination**

This encompasses race, age, gender, height, weight and blood pressure.

- **Previous clinical history collection**

Relevant medical history of patients will be collected, as well as drug use and previous concomitant medication. In detail, the collection mainly included the history of atrial fibrillation, hypertension, diabetes, transient ischemic attack, stroke, and the use of relevant drugs, etc.

- **Key time information**

This encompassed the time when symptoms first appeared, the last time the patient was well, the time of puncture, the time of successful recanalization, and the time of randomization.

- **Laboratory tests of blood**

Hemogram: white blood cell count, neutrophil count, lymphocyte count, platelet count.

Biochemistry: C-reactive protein, procalcitonin, erythrocyte sedimentation rate, random blood glucose, glycosylated hemoglobin, creatinine, urea nitrogen, triglycerides and total cholesterol.

Coagulation indicators: Prothrombin activity (PT), Activated Partial Thromboplastin Time (APTT) and INR.

- **Pre-stroke mRS and baseline NIHSS assessment**

All the assessment were conducted by those who have been trained and certified to use these scales in a blind manner.

- **Endovascular thrombectomy and intra-arterial tenecteplase administration**

In phase Ib, eligible participants received intra-arterial administration of increasing doses of tenecteplase after successful EVT. Patients were treated with up to 4 escalating dose tiers: 0.03125 mg/kg (1/8 i.v dose), 0.0625 mg/kg (1/4 i.v dose), 0. 125 mg/kg (1/2 i.v dose), and 0.1875 mg/kg (3/4 i.v dose).

In phase IIa, two doses (A and B) were chosen by the DSMB and investigators jointly based on the results of the phase Ib study. Eligible patients assigned to the tenecteplase group underwent an infusion of intra-arterial tenecteplase with the assigned dose within 10 minutes after randomization. This infusion was administered through a distal access catheter or microcatheter positioned proximal to the initially occluded artery. In patients allocated to the control group, the procedure was terminated without further intra-arterial thrombolysis.

- **Imaging procedures: non-contrast CT**

Both at admission and within 24 hours after EVT, patients undergo CT scans. And all the images will be blinded review by the Centralized Neuroimaging Core Lab.

Employing the established definitions and normative criteria, the following imaging and angiographic indicators will be extracted according to direct prior experience with these measures and scales:

Baseline:

- ASPECTS
- Hemorrhage
- Baseline occlusive lesion location

Procedure:

- eTICI score
- Arterial occlusive and/or stenosis lesion
- Distal emboli
- Emboli to new territories
- Vessel perforation
- Rupture of the perforator artery
- Arterial dissection

Post-Procedure (24 hour):

- Hemorrhage

Neurological Deterioration or Course Post-Procedure:

- Based on the availability of relevant imaging findings and measures

- **Adverse Events and Serious Adverse Events**

This part was described in section 3.8 of this protocol.

- **Follow-up mRS, NIHSS and EQ-5D-3L assessments**

All the assessments were conducted by those who have been appropriately trained and unaware of the treatment assignment. The NIHSS will be conduct at Day 5-7 or discharge if earlier. And mRS score and EQ-5D-3L will be administered at Day 90.

3.7 Study Discontinuation criteria

Possible causes for early termination may include, but are not limited to, the following:

3.7.1 Site Discontinuation

All the participating centers are carefully selected, having high-volume and experienced interventionalists. Each site needs to conduct research according to the protocol. When the sites violate the study inclusion, fail to produce timely data or significantly deviate from the protocol, the sponsor reserves the right to terminate them. The reasons for early definitive discontinuation of the study are the following:

• Significant safety concerns arising that impact the well-being of the study participants.

• Major deviations from the protocol that potentially threaten the safety of the participants.

3.7.2 Participant Discontinuation

Patients may withdraw their participation in the study whenever they wish. Meanwhile, the investigator may also determine to suspend a participant from the study if they are not conforming to the protocol requirements. The reasons for participant discontinuation from the study are the following:

• Subject decides to withdraw from the study;

• Significant protocol non-compliance;

• Adverse event or other medical condition occurs, which would violate the participant's best interests if continue their involvement in the study;

• Participant meets an exclusion criterion.

Withdrawal of consent will not result in any penalty for that subject. These patients will undergo further treatment according to local medical practices. Data collected prior to the withdrawal will not be discarded and will be used in final analysis. Subjects with unresolved AEs at the time of withdrawal must be followed until the events are resolved, stable, or have reached chronic clinical endpoints, or until death occurs. The reason for participant discontinuation must be documented in the original document and in the CRF table.

3.7.3 Lost to Follow-up

After three attempts to contact the subjects by telephone have failed, a certified letter will be sent to to their last known mailing address or other local equivalent methods. If this communication is also failed, the subjects will be deemed lost to follow-up.

3.8 Adverse Events and Serious Adverse Events

3.8.1 Adverse Events Reporting

The safety assessments will include monitoring and recording AEs. The definition of an adverse event (AE) is any untoward medical occurrence in a patient or clinical trial participant administered a medicinal product, which does not necessarily have a causal relationship with the treatment. During the study, all AEs that happen to the participants will be gathered, regardless of the relationship to the study medication. The evaluation of the severity and relationship of AE are carried out by adhering to the following definitions and criteria.

Severity categorization:

• Mild: The event typically does not interfere with the usual activities of daily life, usually temporary and easily tolerated;

• Moderate: The event causes discomfort that interferes with usual activities of daily living and may require intervention;

• Severe: The event interrupts usual activities of daily living or affects clinical status significantly and warrants intervention;

• Life-threatening: Substantial risk of dying at the time of the event;

• Death.

Relationship of adverse events:

• Definitely Related: The occurrence of the AE was caused by the study drug, implying the existence of evidence or rationales indicating a causal relationship;

• Probably Related: The occurrence time of AE is clearly related to the use of the study drug, unlikely or significantly less likely to be ascribable to another cause, and which follows a clinically reasonable response on withdrawal;

• Possibly Related: The occurrence time of AE is clearly related to the use of the study drug, but which could also be explained by alternative cause. Information on drug withdrawal may be lacking or unclear;

• Unrelated: The occurrence of AE is determined to be definitively caused by extraneous causes (diseases, environment, etc.) and does not meet the criteria for drug relationship listed above.

Serious Adverse Event:

A Serious Adverse Event refers to any unfavorable medical incident (regardless of its connection to the investigational drug) that meets any of the following conditions:

• Fatal (i.e., the AEs actually causes or leads to death);

• Life-threatening (i.e., it refers to an AE in which the patient was at risk of death at the time of the event);

• Requires or prolongs inpatient hospitalization;

• Leads to persistent or significant disability/incapacity (i.e., the AEs causes major interference with the patient's ability to perform normal life functions);

• A congenital defect or birth anomaly in a newborn or infant whose mother was exposed to the study medication;

• A major medical occurrence, in the opinion of the investigator, that could endanger the patient or necessitate medical or surgical treatment to prevent one of the outcomes mentioned above.

NOTE:All serious AEs should be reported within 24 hours through eCRF.

3.8.2 Recording of adverse events

Any AE occurring during the study, regardless of its relationship with the treatment, will be recorded from the moment the ICF is signed until Day 90. All documented AEs will be submitted to the data safety monitoring board (DSMB) during their review for decision-making. Any AE that results in a participant withdrawing from the study, as well as those that continue until the study's conclusion, must to be followed up.

For this clinical study, the follow-up process will not pose any physiological risks to the patients. All treatment medications have been approved and certified by the National Medical Products Administration.

Summaries of patients with AEs by System Organ Class and Preferred Term (MedDRA) will be prepared. Each patient will be counted only once within each category (System Organ Class or Preferred Term). If a patient experiences more than one AE within a category, only the AE with the strongest relationship or the greatest intensity, as appropriate, will be included in the summaries.

4. Statistical Methods

4.1 General remarks

All statistical analyses will be performed using SAS Software version 9.4 or a more recent version within a Windows environment. Data visualization will be achieved through the use of MS Excel 2019 (Microsoft).

Unless specified otherwise, statistical tests will be two-tailed, with a significance level set at $P < 0.05$, and all treatment effect estimates will be accompanied by 95% confidence intervals (CIs). The ITT population will be the basis for the analysis of both primary and secondary efficacy and safety outcomes, ensuring that all patients are analyzed within the group they were randomized to, irrespective of whether they received the allocated treatment.

Categorical data will be presented for each treatment group with counts and percentages, where the denominator for percentages is the number of subjects in the relevant population. Continuous data will be summarized for each group by the number of observations (n), mean, and standard deviation, while some will also be reported as median and interquartile range, as dictated by the clinical relevance of the data. Percentages will be rounded to one decimal place, with 0% and 100% exceptions that will not display decimals. Minimum and maximum values will be rounded to match the precision of the original data, means and medians to one decimal place above the original precision, and standard deviations to two decimal places above the original precision. P values greater than 0.01 will be reported to two decimal places, those between 0.01 and 0.001 to three decimal places, and P values less than 0.001 will be denoted as $P < 0.001$.

4.2 Sample size

4.2.1 Phase Ib

The Endovascular Treatment for Acute Anterior Circulation Ischemic Stroke registry in China showed that the incidence of sICH within 24 hours after EVT was 13.8%. Based on this data, we designed a 14+8 enrollment plan as follows:

1) 14 subjects will be included in the trial for the first time for each dose;

2) If<2 DLT (dose limit toxicity) symptomatic intracranial hemorrhages occur within 24 hours after EVT, the trial advances to the next dose level;

3) If 2/14 DLT, then include 8 more people at the same dose;

4) If 2/14+0-1/8DLT (9.1% to 13.6%), proceed to the next dose level;

5) If 2/14+2/8 DLT (18.2%) or 3/14 DLT, then this dose is deemed unsafe and the prior lower dose is the MTD.

4.2.2 Phase IIa

In Phase IIa randomization will allocate to randomize 46 subjects each into dose group A and dose group B, and 65 subjects into the control group in a 1:1: √2 ratio.

4.3 Population sets

There will the following analysis populations for this study:

1) Full Population Analysis (Intention-to-Treat, ITT)

• This includes all subjects who were randomized.

2) Per Protocol Population

• This encompasses all subjects who did not significantly deviate from the protocol, as determined on a per-subject basis by the trial steering committee immediately before database lock.

3) Safety Population

• This comprises all subjects who received any study treatment (including control), with the exception of subjects who dropped out before receiving any treatment.

4.4 Statistical analyses

4.4.1 Primary outcome Analysis

Dose Escalation (Phase Ib):

The proportion of patients with symptomatic intracranial hemorrhage (SICH) within 24 hours post-treatment is considered the primary safety outcome. The percentage of subjects with sICH within 24 hours after treatment will be presented for each definition by treatment group. Frequency counts and percentages of patients within each category will be provided for categorical data. Subject rates will be

compared between treatment groups using the Chi-square test or Fisher's exact test. The modified Poisson regression will be used to estimate the RR and corresponding 95%CI with treatment group as the independent variable and the presence or absence of SICH as the dependent variable. The treatment effect will be presented as the RR with the corresponding 95% CI.

Dose Expansion (Phase IIa):

The primary endpoint in phase IIa is the proportion of patients with mRS 0-1 at Day 90 and primarily analyzed in the FAS population in the phase IIa. The handling of missing data for the primary endpoint (Proportion of patients with a mRS 0-1 at Day 90) is detailed in 6.2. As the primary endpoint, the mRS score 0-1 at Day 90.

Pooled analysis of phases Ib and IIa:

A prespecified supportive analysis that combines phase Ib and phase IIa will be performed. According to the Ib/IIa combined design, phase Ib patients from the two doses chosen to be used in phase IIa, could be added to the overall patients sample size of dose A and dose B arms of phase IIa patients, respectively. The mRS score of 0-1 at Day 90 will be analyzed using modified Poisson regression, from which risk ratio (RR) with its 95% CI will be estimated. Both adjusted and unadjusted RR and 95% CI will be reported.

4.4.2 Secondary Endpoints Analysis

***Efficacy*:**

Secondary efficacy outcomes encompass mRS 0-4, mRS 0-3, mRS 0-2, mRS 0-1 (or restoration to pre-morbid mRS score), and EQ-5D-3L at Day 90. These outcomes will provide further insights into the therapeutic impact.

All efficacy analyses will be considered exploratory in nature, and thus there will be no hierarchy among the selected parameters. All statistical tests will be conducted at a significance level of 0.05 for the p-value. The specific statistical model for analyzing each of these outcome measures will be detailed in the Statistical Analysis Plan (SAP).

Modified Rankin Scale score:The mRS score will be utilized to assess the proportion of patients with a score ranging from 0 to 4 compared to those with a score of 5 or 6,

from 0 to 3 versus 4 or higher, from 0 to 2 versus 3 or higher, and from 0 to 1 (or equivalent to pre-stroke morbidity) versus 2 or higher, all measured at day 90±14. The adjusted risk ratio will be computed by applying GLM models independently.

Proportion of patients with angiographic changes on the eTICI score after Intra-arterial Tenecteplase: The proportion of patients with angiograpihc changes on the eTICI score after Intra-arterial tenecteplase will be summarized using numbers and proportion, and no between group comparison will be made.

Changes in the NIHSS score 5-7 days after surgery or at discharge: it will be analyzed using win ratio method. NIHSS will be analyzed using win ratio method since NIHSS has been empirically shown to be skewedly distributed.

Quality of life measured with the EQ-5D-3L at Day 90: it will be and analyzed using win ratio method since EQ-5D-3L has been empirically shown to be skewedly distributed.

Safety

Safety outcomes encompass the incidence of overall mortality at 90 (±14) days post-randomization, the proportion of patients experiencing sICH within 24 hours post-treatment, and the proportion of patients with any ICH within 24 hours post-treatment. The safety analysis will be conducted on the Safety Population. The incidences of each outcome will be compared using the Chi-square test or Fisher's exact test. Modified Poisson regression models will be applied to estimate the risk-ratio associated with the treatment effect. The risk ratio with 95% CI will be reported. Patients with missing outcomes will not be included in the regression analysis. Furthermore, Log-Rank tests and Kaplan-Meier estimates will be plotted over the 90-day observation period for mortality. A Cox regression model will be used to calculate the hazard ratio with 95% CI.

4.5 Handling of dropouts and missing or invalid data

Handling of missing or invalid data will be described in the SAP

5. Data handling

5.1 Completion of the electronic CRFs (eCRF)

An electronic Case Report Form (eCRF) will be completed for each study participant, summarizing all clinical screening and study data. Participants will only be referred to in the eCRF by their participant number and initials to maintain participant confidentiality.

5.2 Data management

The CRF and imaging data, which have undergone dual review, will be forwarded to the data management team. The designated individual responsible for the data management team will verify and countersign the receipt form. Upon completion of data entry, the CRF will be retained by the research center.

5.3 SAE reconciliation

At the conclusion of the study, prior to database lock, the sponsor will supply the final SAE database to the Data Manager (DM) for reconciliation purposes. Should any discrepancies arise, appropriate queries will be generated to seek clarification.

5.4 Database lock

Before locking the database, it is imperative to finalize the following activities:

- Ensure all data has been processed;
- Resolve all outstanding queries;
- Review the coding list for completeness and consistency.

5.5 Independent Committee

-Data Safety Monitoring Board (DSMB)

An independent Data Safety Monitoring Board (DSMB) will oversee the study's safety. The DSMB will consist of two stroke neurologists and a statistician who are not involved in the study and are not affiliated with the sponsor. Although the DSMB will review data in a blinded manner (Group A and B), the date of the SAP closure will be set before the first unblinded review to ensure the study's integrity and to prevent any operational bias. Any potential analysis amendments will be documented and justified, if necessary. The study adhered to regulatory recommendations concerning the functions and procedures of these committees.

- Independent Imaging Core Lab (ICL)

An independent Imaging Core Lab (ICL) will be established to regularly review the imaging data that is being accumulated from the ongoing trial. The ICL will consist of two neuroradiologists, a stroke neurologist, and a physician. Their role will be to systematically examine the imaging data as it accrues from the trial. Additionally, the ICL will enforce stringent monitoring to ensure the control of predefined adverse events (AEs) and serious adverse events (SAEs).

6. Quality control / monitoring

The information gathered in this study will be considered confidential under any circumstances.

The trial design has undergone carefully reviewed and received approval from the Steering Committee prior to its submission to the Ethics Committees and Regulatory Authorities for consent. Furthermore, the independent DSMB, and an external SAB have evaluated and approved the protocol and will oversee the trial's progress on an ongoing basis. These committees, composed of recognized independent experts, will maintain a high level of concern for all ethical and safety issues.

The investigators commit to carrying out the trial in line with this trial protocol, the International Conference on Harmonization-Good Clinical Practice (ICH-GCP) Guidelines, and relevant regulatory standards. The investigators assure to provide truthful and reliable data on the CRF, ensuring that all data collected in the CRF is consistent with the source documentation.

Investigators will allow the monitoring process. The primary role of the monitoring team is to assist investigators in ensuring that every aspect of clinical trials is highly ethical, scientific, professional and standardized. The monitoring team will observe the facilities and equipment, as well as interact with the personnel participating in the study, to ensure that all capabilities and staff meet the ICH and GCP standards. The monitoring team will regularly contact the centers via field visits, emails, or phone calls, and will dispatch inspectors to evaluate the trial's progress, ensure that investigators and patients are adhering to the trial protocol, and address any urgent matters. The monitoring team is responsible for monitoring the completion of the CRF and ensures the accuracy of data by directly accessing to source documents. In general, the key elements of inspection and monitoring include (but are not limited to): ICF signature, the recruitment and follow-up of patients, recording and reporting

the serious AE, supply of study drug, treatment compliance, quantity of the study drug, concomitant treatment and data quality.

7. Ethics and regulatory aspects

7.1 Ethical considerations

The research will be carried out in compliance with legal norms and regulations, as well as the Declaration of Helsinki (World Medical Association 2013), the Good Clinical Practice (GCP) standards (ICH 1996, ICH 2008, and ICH E6(R2)), and the fundamental principles outlined in the International Ethical Guidelines for Biomedical Research in humans (Council for International Organizations of Medical Sciences, 2002). Furthermore, the study will adhere to the protocol, GCP guidelines, ICH requirements, and relevant local laws.

7.2 Ethics Committee

The trial will not commence at any center before receiving written approval and authorization from the respective Ethics Committee. During the conduct of trial, any subsequent protocol amendment must be approved by the Ethics Committee before they can be implemented.

7.3 Informed Consent

Prior to initiating any procedure, patients or their legal representatives will be provided with both verbal and written details regarding the study, including its goals, procedures, potential risks, possible benefits, and the rights/obligations of participation. Inform them that they have the right to withdraw from the trial at any stage without facing any discrimination or repercussions, and their medical rights will remain intact. Written Informed Consent must be given after the study's context has been thoroughly explained in clear and understandable language for the participant or their representative. Additionally, the participant or their representative should be allowed to ask questions and receive answers to his/her satisfaction.

8. Insurance

The researchers ensure that patients are covered by insurance for any trial-related harm, following relevant national regulations. A certificate of insurance will be issued to the investigator at the coordinating center where this document is necessary.

9. Study report and publications

The results of the trial will be submitted to the Regulatory Authorities and Ethics Committees. The researchers will deliver an annual safety report along with the final report.

As outlined in the clinical trial protocol, the trial results will be published in a suitable journal for manuscripts or presented at a meeting for abstracts.

By signing the clinical trial protocol, the investigator consents to the publication of the results from this clinical trial.

The trial will be registered at the Chinese Clinical Trial Registry website.

References

1. Zhang X, Xie Y, Wang H, et al. Symptomatic Intracranial Hemorrhage After Mechanical Thrombectomy in Chinese Ischemic Stroke Patients: The ASIAN Score. *Stroke.* 2020;51(9):2690-2696.
2. Collaborators GBDS. Global, regional, and national burden of stroke, 1990-2016: a systematic analysis for the Global Burden of Disease Study 2016. *Lancet Neurol.* 2019;18(5):439-458.
3. Wang Y, Li Z, Zhao X, et al. Stroke care quality in China: Substantial improvement, and a huge challenge and opportunity. *Int J Stroke.* 2017;12(3):229-235.
4. Collaborators GBDS. Global, regional, and national burden of stroke and its risk factors, 1990-2019: a systematic analysis for the Global Burden of Disease Study 2019. *Lancet Neurol.* 2021;20(10):795-820.
5. Ma Q, Li R, Wang L, et al. Temporal trend and attributable risk factors of stroke burden in China, 1990-2019: an analysis for the Global Burden of Disease Study 2019. *Lancet Public Health.* 2021;6(12):e897-e906.
6. Donkor ES. Stroke in the 21(st) Century: A Snapshot of the Burden, Epidemiology, and Quality of Life. *Stroke Res Treat.* 2018;2018:3238165.
7. Powers WJ, Rabinstein AA, Ackerson T, et al. Guidelines for the Early Management of Patients With Acute Ischemic Stroke: 2019 Update to the 2018 Guidelines for the Early Management of Acute Ischemic Stroke: A Guideline for Healthcare Professionals From the American Heart Association/American Stroke Association. *Stroke.* 2019;50(12):e344-e418.
8. Liaw N, Liebeskind D. Emerging therapies in acute ischemic stroke. *F1000Res.* 2020;9.
9. Tissue plasminogen activator for acute ischemic stroke. *N Engl J Med.* 1995;333(24):1581-1587.
10. Hacke W, Kaste M, Bluhmki E, et al. Thrombolysis with alteplase 3 to 4.5 hours after acute ischemic stroke. *N Engl J Med.* 2008;359(13):1317-1329.
11. Campbell BC. Thrombolysis and Thrombectomy for Acute Ischemic Stroke: Strengths and Synergies. *Semin Thromb Hemost.* 2017;43(2):185-190.
12. Murphy LR, Hill TP, Paul K, et al. Tenecteplase Versus Alteplase for Acute Stroke: Mortality and Bleeding Complications. *Ann Emerg Med.* 2023;82(6):720-728.
13. Warach SJ, Dula AN, Milling TJ, Jr. Tenecteplase Thrombolysis for Acute Ischemic Stroke. *Stroke.* 2020;51(11):3440-3451.
14. Goyal M, Menon BK, van Zwam WH, et al. Endovascular thrombectomy after large-vessel ischaemic stroke: a meta-analysis of individual patient data from five randomised trials. *Lancet.* 2016;387(10029):1723-1731.
15. Nogueira RG, Jadhav AP, Haussen DC, et al. Thrombectomy 6 to 24 Hours after Stroke with a Mismatch between Deficit and Infarct. *N Engl J Med.* 2018;378(1):11-21.
16. Ganesh A, Al-Ajlan FS, Sabiq F, et al. Infarct in a New Territory After Treatment Administration in the ESCAPE Randomized Controlled Trial

(Endovascular Treatment for Small Core and Anterior Circulation Proximal Occlusion With Emphasis on Minimizing CT to Recanalization Times). *Stroke.* 2016;47(12):2993-2998.

17. Singh N, Cimflova P, Ospel JM, et al. Infarcts in a New Territory: Insights From the ESCAPE-NA1 Trial. *Stroke.* 2023;54(6):1477-1483.
18. Mujanovic A, Jungi N, Kurmann CC, et al. Importance of Delayed Reperfusions in Patients With Incomplete Thrombectomy. *Stroke.* 2022;53(11):3350-3358.
19. Ames A, 3rd, Wright RL, Kowada M, Thurston JM, Majno G. Cerebral ischemia. II. The no-reflow phenomenon. *Am J Pathol.* 1968;52(2):437-453.
20. Sperring CP, Savage WM, Argenziano MG, et al. No-Reflow Post-Recanalization in Acute Ischemic Stroke: Mechanisms, Measurements, and Molecular Markers. *Stroke.* 2023;54(9):2472-2480.
21. Renu A, Millan M, San Roman L, et al. Effect of Intra-arterial Alteplase vs Placebo Following Successful Thrombectomy on Functional Outcomes in Patients With Large Vessel Occlusion Acute Ischemic Stroke: The CHOICE Randomized Clinical Trial. *JAMA.* 2022;327(9):826-835.
22. Campbell BCV, Mitchell PJ, Churilov L, et al. Tenecteplase versus Alteplase before Thrombectomy for Ischemic Stroke. *N Engl J Med.* 2018;378(17):1573-1582.
23. Jiacheng Huang CG, Jie Yang, Xiaolei Shi, Chang Liu, Jiaxing Song, Fengli Li, Weilin Kong, Shitao Fan, Zhouzhou Peng, Shihai Yang, Jinfu Ma, Xu Xu, Linyu Li, Zhixi Wang, Nizhen Yu, Wenzhe Sun, Chengsong Yue, Xiang Liu, Dahong Yang, Cheng Huang, Duolao Wang, Raul G. Nogueira, Thanh N. Nguyen, Jeffrey L. Saver, Yangmei Chen, Wenjie Zi. Adjunctive Intra-arterial Tenecteplase after Successful Endovascular Thrombectomy in Patients with Large Vessel Occlusion Stroke (POST-TNK): Study Rationale and Design. *European Stroke Journal.* 2024.
24. Huo X, Luo G, Sun D, et al. Intra-arterial tenecteplase after successful endovascular therapy (ANGEL-TNK): protocol of a multicentre, open-label, blinded end-point, prospective, randomised trial. *Stroke and vascular neurology.* 2024.
25. Huang JH, Su QM, Yang J, et al. Sample sizes in dosage investigational clinical trials: a systematic evaluation. *Drug design, development and therapy.* 2015;9:305-312.
26. Yuan Y, Hess KR, Hilsenbeck SG, Gilbert MR. Bayesian Optimal Interval Design: A Simple and Well-Performing Design for Phase I Oncology Trials. *Clinical cancer research : an official journal of the American Association for Cancer Research.* 2016;22(17):4291-4301.
27. Wang Y, Han S, Qin H, et al. Chinese Stroke Association guidelines for clinical management of cerebrovascular disorders: executive summary and 2019 update of the management of high-risk population. *Stroke and vascular neurology.* 2020;5(3):270-278.

Appendix table 1 Modified Rankin Scale

The modified Rankin Scale (mRS) is an ordinal hierarchical scale ranging from 0 to 6, with higher scores indicating more severe disability. A score of 6 has been added to signify death.

Category	Short description	Long description
0	No symptoms	No symptoms
1	Symptoms, no disability	Minor symptoms that do not interfere with lifestyle
2	Slight disability	Slight disability, symptoms that lead to some restriction in lifestyle, but do not interfere with the patient's capacity to look after himself.
3	Moderate disability	Moderate disability, symptoms that significantly restrict lifestyle and prevent totally independent existence
4	Moderately severe disability	Moderately severe disability, symptoms that clearly prevent independent existence though not needing constant attention
5	Severe disability	Severe disability, totally dependent patient requiring constant attention day and night.
6	Death	Death

Appendix table 2 Expanded Treatment in Cerebral Ischemia (eTICI) Scale

eTICI grade	Description
0	No reperfusion of the target downstream territory
1	Contrast passes the occlusion but does not result in distal perfusion
2a	Partial reperfusion of<50% of the target downstream territory
2b50	Partial reperfusion of 50%-66% of the target downstream territory
2b67	Partial reperfusion of 67%-89% of the target downstream territory
2c	Partial reperfusion of 90%-99% of the target downstream territory
3	Complete reperfusion of the target downstream territory

Appendix table 3 NIHSS

The NIHSS is an ordinal hierarchical scale to evaluate the severity of stroke by assessing a patient's performance. (23) Scores range from 0 to 42, with higher scores indicating a more severe deficit. Administer stroke scale items in the order listed. Record performance in each category after each subscale exam. Do not go back and change scores. Follow directions provided for each exam technique. Scores should reflect what the patient does, not what the clinician thinks the patient can do. The clinician should record answers while administering the exam and work quickly. Except where indicated, the patient should not be coached (i.e. repeated requests to patient to make a special effort).

Instructions	Scale definition
la. Level of consciousness. The investigator must choose a response if a full evaluation is prevented by such obstacles as an endotracheal tube,language barrier, orotracheal trauma/bandages.A 3 is scored only if the patient makes no movement (other than reflexive posturing)in response to noxious stimulation.	0=Alert;keenly responsive.<br>1=Not alert;but arousable by minor stimulation to obey,answer,or respond.<br>2=Not alert;required repeated stimulation to attend,or is obtunded and requires strong or painful stimulation to make movements (not stereotyped).<br>3=Responds only with reflex motor or autonomic effects or totally unresponsive, flaccid and areflexic.
1b. LOC Questions: The patient is asked the month and his/her age. The answer must be correct – there is not partial credit for being close. Phasic and stuporous patients who do not comprehend the questions will score 2. Patients unable to speak because of endotracheal intubation, orotracheal trauma, severe dysarthria from any cause, language barrier, or any other problem not secondary to aphasia are given a 1. It is important that only the initial answer be graded and that the examiners not "help" the patient with verbal or non-verbal clues.	0 =Answers both questions correctly.<br>1 =Answers one question correctly.<br>2 =Answers neither question correctly.
1c. LOC Commands: The patient is asked to open and close the eyes and then to grip and release the non-paretic hand. Substitute another one step command if the hand cannot be used. Credit is given if an unequivocal attempt is made but not completed due to weakness. If the patient does not respond to command, the task should be demonstrated to him or her (pantomime), and the result scored (i.e. follows none, one or two commands). Patients with trauma, amputation, or other physical impediments should be given suitable one-step commands. Only the first attempt is scored.	0 = Performs both tasks correctly.<br>1 = Performs one task correctly.<br>2 = Performs neither task correctly.
2.Best Gaze: Only horizontal eye movements will be tested. Voluntary or reflexive (oculocephalic)eye movements will be scored,but caloric testing is not done.If the patient has a conjugate deviation of the eyes that can be overcome by voluntary or reflexive activity,the score will be a 1.Ifa patient has an isolated peripheral nerve paresis (CN III,IV or VD),s score a 1.Gaze is testable in all aphasic patients.Patients with ocular trauma,bandages,preexisting blindness,or other disorder of visual acuity or fields should be tested with reflexive movements, and a choice made by the investigator.Establishing eye contact and then moving about the patient from side to side will occasionally clarify the presence of a partial gaze palsy.	0= Normal.<br>1= Partial gaze palsy; gaze is abnormal in one or both eyes, but forced deviation or total gaze paresis is not present.<br>2= Forced deviation; or total gaze paresis not overcome by the oculocephalic maneuver.

3.Visual: Visual fields (upper and lower quadrants)are tested by confrontation,using finger counting or visual threat,as appropriate.Patients may be encouraged,but if they look at the side ofthe moving finger appropriately,this can be scored as normal.If there is unilateral blindness or enucleation,visual fields in the remaining eye are scored.Score 1 only ifa clear-cut asymmetry,including quadrantanopia,is found.If patient is blind from any cause,score 3. Double simultaneous stimulation is performed in this case. Ifthere is extinction,the patient receives a 1,and the results are used to respond to item 11.	0= No visual loss.<br>1= Partial hemianopia.<br>2= Complete hemianopia.<br>3= Bilateral hemianopia (blind including cortical blindness)
4.Facial palsy: Ask or use pantomime to encourage the patient to show teeth or raise eyebrows and close eyes.Score symmetry of grimace in response to noxious stimuli in the poorly response or non-comprehending patient.If facial trauma/bandages, orotracheal tube,tape or other physical barriers obscure the face, these should be removed to the extent possible.	0 = Normal symmetrical movements.<br>1= Minor paralysis (flattened nasolabial fold, asymmetry on smiling)<br>2= Partial paralysis (total or near-total paralysis of lower face)<br>3= Complete paralysis of one or both sides<br>(absence of facial movement in the upper and lower face).
5.Motor arm: The limb is placed in the appropriate position: extend the arms (palms down)90 degrees (ifsitting)or 45 degrees (if supine).Drift is scored ifthe arm falls before 10 seconds.The aphasic patient is encouraged using urgency in the voice and pantomime,but not noxious stimulation.Each limb is tested in turn,beginning with the non-paretic arm.Only in the case of amputation or joint fusion at the shoulder,the examiner should record the score asuntestable (UN),and clearly write the explanation for this choice.	0= No drift; limb holds 90 (or 45) degrees for full 10 seconds.<br>1= Drift; limb holds 90 (or 45) degrees, but<br>drifts down before full 10 seconds; does not hit bed or other support.<br>2= Some effort against gravity; limb cannot get to or maintain (if cued) 90 (or 45) degrees,<br>drifts down to bed, but has some effort against gravity.<br>3= No effort against gravity; limb falls. 4= No movement.<br>UN = Amputation or joint fusion: explain:<br>5a = Left Arm. 5b = Right arm.
6.Motor leg: The limb is placed in the appropriate position: hold the leg at 30 degrees (always tested supine).Drift is scored ifthe leg falls before 5 seconds.The aphasic patient is encouraged using urgency in the voice and pantomime,but not noxious stimulation.Each limb is tested in turn,beginning with the non-paretic leg.Only in the case of amputation or joint fusion at the hip,the examiner should record the score as untestable (UN),and clearly write the explanation for this choice.	0= No drift; leg holds 30-degree position for full 5 seconds.<br>1= Drift; leg falls by the end of the 5-second period but does not hit bed.<br>2= Some effort against gravity; leg falls to bed by 5 seconds, but has some effort against gravity.<br>3= No effort against gravity; leg falls to bed immediately.<br>4= No movement.<br>UN = Amputation or joint fusion: explain:<br>6a. Left Leg<br>6b. Right Leg.

7.Limb ataxia:This item is aimed at finding evidence ofa unilateral cerebellar lesion.Test with eyes open.In case of visual defect,ensure testing is done in intact visual field. The finger-nose-finger and heel-shin tests are performed on both sides,and ataxia is scored only ifpresent out ofproportion to weakness.Ataxia is absent in the patient who cannot understand or is paralyzed.Only in the case of amputation or joint fusion, the examiner should record the score as untestable (UN), and clearly write the explanation for this choice.In case of blindness,test by having the patient touch nose from extended arm position.	0-Absent.<br>1=Present in one limb.<br>2=Present in two limbs.<br>UN = Amputation or joint fusion: explain:

8.Sensory:Sensation or grimace to pinprick when tested,or withdrawal from noxious stimulus in the obtunded or aphasic patient.Only sensory loss attributed to stroke is scored as abnormal and the examiner should test as many body areas(arms [not hands],legs,trunk,face)as needed to accurately check for hemisensory loss.A score of2,'severe or total sensory loss',should only be given when a severe or total loss of sensation can be clearly demonstrated.Stuporous and aphasic patients will,therefore,probably score 1 or 0.The patient with brainstem stroke who has bilateral loss of sensation is scored 2. If the patient does not respond and is quadriplegic,score 2. Patients in a coma (item la=3)are automatically given a 2 on this item.	0= Normal; no sensory loss.<br>1= Mild-to-moderate sensory loss; patients feel pinprick is less sharp or is dull on the affected side; or there is a loss of superficial pain with pinprick, but patient is aware of being touched.<br>2= Severe to total sensory loss; patient is not aware of being touched in the face, arm and leg.
9.Best language:A great deal of information about comprehension will be obtained during the preceding sections of the examination.For this scale item,the patient is asked to describe what is happening in the attached picture,to name the items on the attached naming sheet and to read from the attached list of sentences.Comprehension is judged from responses here,as well as to all ofthe commands in the preceding general neurological exam.If visual loss interferes with the tests,ask the patient to identify objects placed in the hand,repeat,and produce speech.The intubated patient should be asked to write.The patient in a coma (item 1a=3)will automatically score 3 on this item.The examiner must choose a score for the patient with stupor or limited cooperation,but a score of 3 should be used only if the patient is mute and follows no one-step commands.	0=No aphasia;normal<br>1=Mild-to-moderate aphasia;some obvious loss offluency or facility of comprehension, without significant limitation on ideas expressed or form of expression.Reduction of speech and/or comprehension,however,makes conservation about provided materials difficult or impossible.For example,in conversation about provided materials,examiner can identify picture or naming card content from patient's response.<br>2=Severe aphasia;all communication is through fragmentary expression;great need for inference,questioning,and guessing by the listener.Range of information that can be exchanged is limited;listener carries burden of communication.Examiner cannot identify materials provided from patient response.<br>3 Mute,global aphasia:no usable speech or auditory comprehension.
10.Dysarthria:If patient is thought to be normal,an adequate sample of speech must be obtained by asking patient to read or repeat words from the attached list.If the patient has severe aphasia,the clarity of articulation of spontaneous speech can be rated.Only if patient is intubated or has other physical barriers to producing speech,the examiner should record the score as untestable (UN),and clearly write an explanation for this choice.Do not tell the patient why he or she is being tested.	0=Normal.<br>1=Mild-to-moderate dysarthria;patient slurs at least some words and,at worst,can be understood by some difficulty.<br>2=Severe dysarthria:patient's speech is so slurred as to be unintelligible in the absence of or out of proportion to any dysphasia,or is mute/anarthric.<br>UN Intubated or other physical barrier.

11.Extinction and Inattention (formerly Neglect):Sufficient information to identify neglect maybe obtained during the prior testing.If the patient has a severe visual loss preventing visual double simultaneous stimulation,and the cutaneous stimuli are normal,the score is normal.If the patient has aphasia but does appear to attend to both sides,the score is normal.The presence of visual spatial neglect or anosagnosia may also be taken as evidence of abnormality.Since the abnormality is scored only if present,the item is never untestable.	0=No abnormality.<br>1=Visual,tactile,auditory,spatial,or personal inattention or extinction to bilateral simultaneous stimulation in one ofthe sensory modalities.<br>2=Profound hemi-inattention or extinction to more than one modality;does not recognize own hand or orients to only one side ofspace.

Appendix table 4 EUROQOL 5D-3L

The EuroQoL 5-dimensions 3-level (EQ-5D-3L) questionnaire is a standardized measure of health outcome that has been used extensively inpatients with stroke.
Under each heading, please tick the ONE box that best describes your health TODAY.

Mobility

I have no problems in walking about ☐

I have some problems in walking about ☐

I am confined to bed ☐

Self-care

I have no problems with self-care ☐

I have some problems washing or dressing myself ☐

I am unable to wash or dress myself ☐

Usual activities (e.g. work, study, housework, family or leisure activities)

I have no problems with performing my usual activities ☐

I have some problems with performing my usual activities ☐

I am unable to perform my usual activities ☐

Pain/discomfort

I have no pain or discomfort ☐

I have moderate pain or discomfort ☐

I have extreme pain or discomfort ☐

Anxiety/depression

I am not anxious or depressed ☐

I am moderately anxious or depressed ☐

I am extremely anxious or depressed ☐

Appendix table 4 EUROQOL 5D-3L

- We would like to know how good or bad your health is TODAY.
- This scale is numbered from 0 to 100.
- 100 means the best health you can imagine.
  0 means the worst health you can imagine.
- Mark an X on the scale to indicate how your health is TODAY.
- Now, please write the number you marked on the scale in the box below.

YOUR HEALTH TODAY = ☐

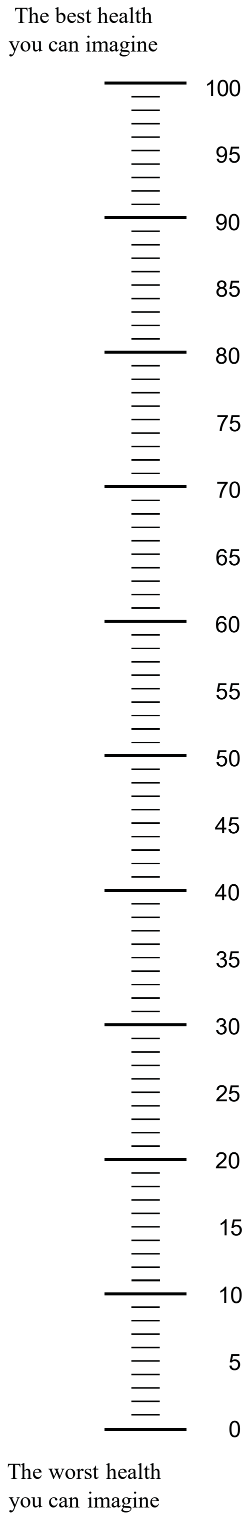

In previous versions of the EQ-5D-3L, the numerical scale straddled the EQ VAS (provided in the annex for reference). Users are encouraged to use the latest version of the EQ-5D-3L in new studies.
