# Supplementary material for: Intra-Arterial Tenecteplase After Successful Reperfusion in Large Vessel Occlusion Stroke: A Randomized Clinical Trial
Source: JAMA Neurol. 2025 Jul 5;82(9):895–904. doi: 10.1001/jamaneurol.2025.2036 (PMC12228979; doi:10.1001/jamaneurol.2025.2036)
Supplement: Supplement 2. — Statistical Analysis Plan. [file jamaneurol-e252036-s002.pdf]

DATE STUDY

The Optimal Therapeutic Dosage of Adjunctive Intra-arterial Tenecteplase following Successful Endovascular Thrombectomy in Patients with Large Vessel Occlusion Acute Ischemic Stroke

A Prospective, Multi-center, Open-label, Blinded Endpoint Phase Ib/IIa clinical trial

Statistical Analysis Plan

Principal Investigator:

Pro Zhenhua Zhou, MD, PhD, Professor of Neurology

Southwest Hospital, Army Medical University

SAP Authors:

Xianhua Hou, MD, PhD; Yuxuan He, MD; Southwest Hospital, Army Medical University, Chongqing, China

Duolao Wang, PhD, Global Health Trials Unit, Liverpool School of Tropical Medicine, Liverpool, United Kingdom

SIGNATURE PAGE

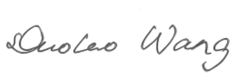

Duolao Wang **Date**

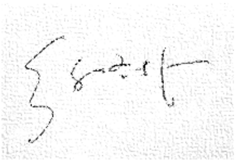

Gelin Xu **Date**

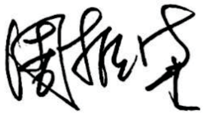

Zhenhua Zhou **Date**

TABLE OF CONTENTS

1. Introduction 1
2. Purpose of Statistical Analysis Plan 1
3. Study Objectives and Designs 2
4. Sample Size 4
5. Analysis Populations 5
6. Statistical Analysis 6
7. Tables and Figures for the Main Paper 12
8. Changed to Planned Analyses 12
9. Fundings 12
10. APPENDIX 13
Appendix table 1 Modified Rankin Scale 13
Appendix table 2 Extended Treatment In Cerebral Ischemia (eTICI) Scale 14
Appendix table 3 National Institute of Health Stroke Scale 15
Appendix table 4 EUROQOL-5D-3L 20
Mobility 20
Self-care 20
Usual activities (e.g. work, study, housework, family or leisure activities) 20
Pain/discomfort 20
Anxiety/depression 20
Appendix Table 4 EUROQOL 5D-3L 21

LIST OF ABBREVIATIONS

Abbreviations	Definitions
AE	Adverse Event
AIS	Acute Ischemic Stroke
AOL	Arterial occlusive lesion classification
APTT	Activated Partial Thromboplastin Time
ASPECTS	the Alberta Stroke Program Early CT Score
BI	Barthel Index
CI	Confidence interval
CONSORT	Consolidated Standards of Reporting Trials
cOR	Common odds ratio
CT	Computed tomography
CTA	Computed tomography angiography
CRF	Case Report Form
CRO	Contract Research Organization
DSA	Digital substraction angiography
DSMB	Data Safety Monitoring Board
EC	Ethics committee
eTICI	extended treatment in cerebral ischemia scale
EQ5D-3L	EuroQol-5 dimensions-3 level
EVT	Endovascular treatment
IAT	Intra-arterial treatment
INR	International normalized ratio
ITT	Intention-to-treat
IVT	Intravenous treatment
LOC	Level of consciousness
mAOL	Modified arterial occlusive lesion classification
MCA	Middle cerebral artery

Abbreviations	Definitions
MedDRA	Medical Dictionary for Drug Regulatory Activities
MRI	Magnetic resonance imaging
mRS	Modified Rankin scale
MT	Mechanical thrombectomy
NCCT	Non-contrast computed tomography
NIHSS	National Institute of Health Stroke Scale
PPS	Per-protocol set
PT	Preferred term
SAE	Serious adverse event
SAP	Statistical analysis plan
SAS	Statistical analysis system
SD	Standard deviation
sICH	Symptomatic intracerebral hemorrhage
SOC	System organ class

1. Introduction

The Statistical Analysis Plan (SAP) has been meticulously crafted in alignment with the latest study protocol (Version 1.0, dated 1-**June**-2023) and the Case Report Form (CRF, Version 1.0, dated 1-**June**-2023). It delineates the comprehensive statistical analysis strategies and methodologies that will be employed in the data analysis phase of the study entitled "A Prospective, Multi-center, Open-label, End-point, Blinded Phase Ib/IIa clinical trial of Adjunctive Intra-arterial Tenecteplase (TNK) following Successful Endovascular Thrombectomy in Patients with Large Vessel Occlusion Acute Ischemic Stroke".

The structure and content of this SAP provides sufficient details to meet the requirements determined by the National Medical Products Administration (NMPA) and International Conference on Harmonization of Technical Requirements for Registration of Pharmaceuticals for Human Use (ICH-E9): Guidance on Statistical Principles in Clinical Trials[1]. All work planned and reported for this SAP will follow internationally recognized guidelines, published by the American Statistical Association[2] and the Royal Statistical Society[3], for statistical practice.

In preparing this SAP, the following documents were reviewed:

- Clinical Study Protocol.
- Case report forms (CRFs).
- ICH-E9 Guidance on Statistical Principles for Clinical Trials.
- Guidelines for the Content of Statistical Analysis Plans in Clinical Trials.

Readers of this SAP are encouraged to also read the clinical trial protocol to understand the implementation details of this study, the operational aspects of clinical evaluation, and the schedule of patients completing this study.

2. Purpose of Statistical Analysis Plan

The purpose of this SAP is to outline the planned analyses to be completed to support the completion of the clinical study report. The planned analyses identified in this SAP will be included in regulatory submissions and/or future manuscripts. In addition, exploratory analyses not necessarily identified in this SAP may be conducted

to support the clinical development plan. Any post-hoc, or unplanned, analyses outside of this SAP will be clearly identified in the respective clinical study report.

3. Study Objectives and Designs

This clinical study aims to assess the optimal dose, safety and efficacy of intra-arterial TNK in large vessel occlusion patients who achieved successful reperfusion. The study is designed as a phase Ib/IIa trial, in which the two dosages chosen during the phase Ib, determined by safety criteria, will be administered in the subsequent phase IIa.

3.1 Study objective

The primary objective of DATE trial is to assess the safety and efficacy of adjunctive intra-arterial Tenecteplase following successful endovascular thrombectomy in patients with acute ischemic stroke due to large vessel occlusion.

3.2 Study design

The DATE trial will be divided into two parts: the first part (phase Ib) is a pilot dose-escalation safety study, and the second one (phase IIa) is an exploratory study to assess safety and efficacy of adjunctive intra-arterial tenecteplase after successful EVT in patients with LVO stroke at two different doses. After the completion of phase Ib, the data safety monitoring board (DSMB) unblinded to study groups will select 2 doses (A or B) jointly with the investigators to be tested in phase IIa according to initial safety results.

Dose Escalation (Phase Ib):

This is a single arm phase, during this phase, participants received intra-arterial administration of different doses of tenecteplase after successful EVT. Patients will be treated in 4 escalating dosage level: 0.03125 mg/kg (1/8 i.v dose), 0.0625 mg/kg (1/4 i.v dose),0.125 mg/kg (1/2 i.v dose), and 0.1875 mg/kg (3/4 i.v dose).

According to the Chinese Acute Anterior Circulatory Ischemic Stroke Endovascular Treatment Registry Study, 13.8% of patients experienced symptomatic intracranial hemorrhage (sICH) within 24 hours of receiving EVT[4]. Using sample sizes typical for dose escalation phase 1 clinical trials[5,6], we used a 14+8 design scheme with the occurrence of sICH within 24 hours after EVT as the dose-limiting toxicity. At each tier, 14 patients were enrolled, beginning with the lowest prespecified dose. If fewer than 2 of 14 subjects developed sICH, the trial advanced to the next tier dose. If 2 of the 14 patients developed sICH, 8 more patients were enrolled at that dose. If 0-1 of the additional 8 patients developed sICH, the trial advanced to the next tier dose. If 2 of the additional 8 patients developed sICH or 3 of the first 14 developed sICH, that dose was deemed not tolerated and the immediately preceding dose was the estimated maximum tolerated dose.

Dose Expansion (Phase IIa):

Two doses (A and B) will be chosen by DSMB and the investigators jointly according to the results in phase Ib. It is decided that a total of 157 new patients are required for enrollment, with 46 patients assigned to dose A group, 46 patients assigned to dose B group, and an additional 65 patients allocated to the control group.

During this phase, 3 arms will be studied and patients will be randomized to receive 1 of the 2 selected doses of tenecteplase, or to the control group in a 1:1: $\sqrt{2}$ ratio, which in turn yields probabilities of assignment of 0.293, 0.293, and 0.414, respectively. Patients will be randomly assigned by using a real-time internet-based system. This process is automated from a study startup, which allows for complete concealment of the sequence of allocation.

3.3 Study Endpoints

3.3.1 Primary endpoints

Dose Escalation (Phase Ib):

The primary endpoint is the incidence of symptomatic intracranial hemorrhage(sICH) at 24 hours (binary). The detailed definition of the symptomatic intracranial hemorrhage was provided at the Protocol.

Dose Expansion (Phase IIa):

The primary endpoint will be the proportion of patients with a mRS 0 to 1 at 90 days (binary). The assessment will be conducted by two independent certified physicians who are blinded to the grouping information. To ensure the reliability, evaluability, and traceability of the mRS score, we kept the patients' follow-up video or audio version for 90 days, except for those who died or refused to take the video.

3.3.2 Secondary endpoints

Dose Escalation (Phase Ib):

1. 90-day modified Rankin scale (mRS) score 0-1 (%) (binary);

2. Proportion of patients with functional independence (mRS score 0 to 2) at 90 days (binary);

3. Shift in the distribution of mRS scores at 90 days (ordinal);

4. Proportion of patients with angiographic changes on the eTICI score after Intra-arterial Tenecteplase thrombolysis (binary). To that aim, all the baseline angiographies will be scored at the core lab by central and blinded reviewers using the eTICI and classified as eTICI2b, eTICI2c, and eTICI3. The post treatment angiographies will be

scored using the eTICI and classified as “improved”, “worsened” or “unchanged” with regard to the baseline eTICI score.

5. Changes in the national institutes of health stroke scale (NIHSS) score 5-7 days from baseline (continuous, non-normal);

6. Quality of life measured with the EuroQol Group 5-Dimension Self-Report Questionnaire (EQ-5D-3L) at 90 days (continuous, non-normal);

7. Mortality within 90 days (binary).

Dose Expansion (Phase IIa):

1. Proportion of patients with functional independence (mRS score 0 to 2) at 90 days (binary);

2. Shift in the distribution of mRS scores at 90 days(ordinal);

3. Proportion of patients with angiographic changes on the eTICI score after Intra-arterial Tenecteplase thrombolysis (binary);

4. Changes in the National Institutes of Health Stroke Scale (NIHSS) score 5-7 days after surgery (continuous, non-normal);

5. Quality of life measured with the EuroQol Group 5-Dimension Self-Report Questionnaire (EQ-5D-3L) at 90 days (continuous, non-normal);

6. Symptomatic intracerebral hemorrhage (sICH) rate within 24 h (binary);

7. Mortality within 90 days (binary).

4. Sample Size

The following analysis populations are planned for the studies.

Dose Escalation (Phase Ib):

The registration study of endovascular thrombectomy (EVT) for acute anterior circulation ischemic stroke in China showed that the incidence of symptomatic intracranial hemorrhage within 24 hours after EVT was 13.8%. Based on this data, we have designed a 14+8 enrollment plan as follows:

(1)14 subjects were included in the trial for the first time for each dose;

(2) If<2dose limit toxicity (DLT, defined as the sICH occurrence with in 24 hours after EVT), the trial advances to the next dose level;

(3) If 2/14 DLT, then include 8 more people at the same dose ;

(4) If 2/14+0-1/8DLT (9.1% to 13.6%), proceed to the next dose level;

(5) If 2/14+2/8DLT (18.2%) or 3/14DLT, then this dose is the estimated maximum tolled dose (MTD).

Dose Expansion (Phase IIa):

Phase IIa plans to randomize 46 subjects each into dose group A and dose group B, and 65 subjects into the control group in a 1:1: √2 ratio. This approach is similar to the sample size calculation method used in the APRIL study[7].

5. AnalysisPopulations

5.1 Intention-to-Treat (ITT) population

The ITT population included all patients who have been randomized into the trial and have a documented record of receiving any quantity of the study drug, irrespective of whether they received the intended treatment or adhered to the protocol until its conclusion. This ITT population serves as the primary analytical cohort for assessing efficacy endpoints, with subjects being analyzed in accordance with the treatment arm they were assigned during the randomization process. ITT is equivalent to Full Analysis Set (FAS) used in some trial reports, and we will use either ITT or FAS in the trial report.

The ITT analysis strategy for DATE is outlined as follows:

- It is grounded in an ITT design, which intends to gather comprehensive outcome data for all randomized participants;
- The primary analysis maintains participants in their original randomized groups, examines all accessible outcome data, and is valid under a specified plausible assumption regarding missing data;
- Sensitivity analyses are conducted, taking into account a variety of plausible alternative assumptions about the missing data;
- All randomized individuals are incorporated into the sensitivity analyses.

5.2 Per-Protocol Set (PPS)

PPS is a subset of the ITT population, encompassing all randomized participants who have undergone treatment in the study without any significant protocol deviations that could notably affect the interpretation of efficacy outcomes. The specific criteria for protocol deviations will be established no later than prior to the database lock. The PPS will serve as the basis for the primary efficacy and safety analyses. Participants included in the PPS must meet all the following essential criteria:

- Received but did not finish the treatment with the study drug, or the dose of the study drug was administered outside the recommended dosage.
- Failed to meet the inclusion or exclusion criteria.
- Essential information is missing.

A list of patients to be excluded from the randomized cohort for the PP-Efficacy analysis will be determined and confirmed by the Steering Committee before the unblinding process.

5.3 Safety Population

The Safety Population comprises all patients who have received any dosage of the study drug. Should there be any deviations from the randomization protocol, participants will be categorized based on the treatment they actually received. Assignment of patients to various populations will occur before the database is unblinded. Patients who withdraw their informed consent immediately following randomization and are not set to receive any treatment should be excluded from the Safety Population.

6. Statistical Analysis

6.1. Analysis Principles and General Considerations

This SAP outlines two distinct research phases: 1) Phase Ib, which focuses on clinical trial safety during dose escalation, and 2) Phase IIa, which assesses both safety and efficacy during the dose-confirmation phase. In phase Ib, four incrementally increasing doses of DATE have been scheduled to ascertain the safety profile of the drug and the appropriate dosage levels. The analysis will be based on the assumption of a progressive (monotonic) relationship, meaning that any dose exceeding an unsafe threshold will also be deemed unsafe. Key potential predictors, such as patient age (<70 vs. ≥70y) and NIHSS score (<15 vs. ≥15), have been balanced at the time of randomization using the minimization method. This dynamic allocation technique optimizes resource utilization in terms of sample size and patient distribution across all trial arms, ensuring the best possible spread of two characteristics of interest. In phase IIa, which is a dose extension

The phase Ib participants will be combined with those in phase IIa (provided comparability is preserved) to enhance statistical power as a supportive analysis. To effectively integrate these phases, the study design, follow-up procedures, and data collection will be rigorously standardized across both the phase Ib and phase IIa segments of the trial.

All statistical analyses will be performed using SAS Software version 9.4 or R Version 4.1. or higher. Data visualization will be achieved through the use of MS Excel 2019 (Microsoft).

Unless specified otherwise, statistical tests will be two-tailed, with a significance level set at $P < 0.05$, and all treatment effect estimates will be accompanied by 95%

confidence intervals (CIs). The ITT population in each phase will be the basis for the analysis of both primary and secondary efficacy and safety outcomes, ensuring that all patients are analyzed within the group they were randomized to, irrespective of whether they received the allocated treatment.

Categorical data will be presented for each treatment group with counts and percentages, where the denominator for percentages is the number of subjects in the relevant population. Continuous data will be summarized for each group by the number of observations (n), mean, and standard deviation, while some will also be reported as median and interquartile range, as dictated by the clinical relevance of the data. Percentages will be rounded to one decimal place, with 0% and 100% exceptions that will not display decimals. Minimum and maximum values will be rounded to match the precision of the original data, means and medians to one decimal place above the original precision, and standard deviations to two decimal places above the original precision. *P* values greater than 0.01 will be reported to two decimal places, those between 0.01 and 0.001 to three decimal places, and *P* values less than 0.001 will be denoted as $P < 0.001$.

6.2 Methods for Withdrawals and Missing Data

If any participant withdraws from the study early (prior to the final visit day 90 ± 14 for evaluation), they must complete a withdrawal visit as documented in the Case Report Form. The reasons for withdrawal will be summarized in a table. Participants who withdrew before the study's conclusion but provided at least one post-baseline measurement for a specific endpoint will be included in the analysis. Participants who fail to attend any visits after study enrollment will be excluded from endpoint analyses due to the absence of post-baseline data.

Efforts will be made to minimize missing data, especially for the 90-day outcome assessment, by utilizing modern communication tools. Participants, their families, and doctors will maintain WeChat and phone contact to prevent loss to follow-up. Nevertheless, some missing data may be unavoidable. Missing values for age, baseline NIHSS score, baseline ASPECTS, time from stroke onset to enrollment, and occlusion site will be imputed using multiple imputation by fully conditional specification regression for continuous variables or fully conditional specification logistic regression for binary and ordinal variables. The handling of missing data for primary, secondary, and safety endpoints will be detailed in a separate section of the statistical analysis.

6.3 Multicenter Studies

For the primary analysis, no adjustment will be made for the center based on the following rationale:

The DATE study is conducted in two parts (phase Ib and phase IIa) to enroll AIS patients across approximately 30 stroke centers in China. Nevertheless, it is anticipated that some centers will recruit significantly fewer patients, while a few larger centers will enroll substantially more. This distribution pattern serves as the rationale for not adjusting the primary analysis by center. As cited from the International Conference on Harmonization 9 guidelines,

“In some trials, for example some large mortality trials with very few subjects per centre, there may be no reason to expect the centres to have any influence on the primary or secondary variables because they are unlikely to represent influences of clinical importance.”

6.4 Demographic and Baseline Characteristics

The demographic profile and baseline characteristics of the study subjects will be detailed using descriptive statistics for each treatment arm. The demographic data to be presented and analyzed for participants in each group are age, and gender. Baseline characteristics, which are not exhaustive, encompass medical history (such as hypertension, atrial fibrillation, coronary heart disease, smoking habits, diabetes mellitus, hyperlipidemia, and history of stroke, pre-treatment mRS score, initial NIHSS score, systolic and diastolic blood pressure levels, baseline ASPECTS score, location of occlusion, serum glucose levels, time from stroke onset to randomization, time from stroke onset to the start of intra-arterial injection of study drug administration, time from stroke onset to recanalization, and time from puncture to recanalization. Summaries will be furnished for the full analysis set, per-protocol, and as-treated populations.

The continuous data will be presented as either the mean and standard deviation or the median and interquartile range, as appropriate; categorical data will be presented as n (%). For the comparison of two continuous datasets, the Kruskal-wallis test will be employed, while the Chi-squared tests or Fisher exact test will be utilized for comparing two sets of categorical data. If the proportion of missing values exceeds 5%, the denominator will be indicated in the footnote of the respective summary table.

6.5 Analysis of outcomes

With regard to phase Ib, analyses will be regarded as descriptive. With regard to phase IIa, both descriptive and model-based approaches (tests and 95% CIs) will be performed.

A prespecified pooled analysis of phase Ib and IIa will be performed to compare 2 selected doses of tenecteplase with the control group. All efficacy analyses will be performed primarily in the FAS and then repeated in the PPS.

In the covariates-adjusted analysis, the following variables will be adjusted.

- Age (Continuous);
- Baseline NIHSS score (Continuous);
- Baseline ASPECTS score (Continuous);
- Time from stroke onset to enrollment (Continuous);
- Occlusion location (Categorical).

If a covariates-adjusted model does not converge, inverse probability of treatment weighting (IPTW) method will be used.

6.5.1 Handling of Multiplicity

Due to the exploratory nature of this study, no adjustments for multiple comparisons will be made.

6.5.2 Analysis of the primary endpoint

Dose Escalation (Phase Ib):

The proportion of patients with symptomatic intracranial hemorrhage (sICH) within 24 hours post-treatment is considered the primary safety outcome. The percentage of subjects with SICH within 24 hours after treatment will be presented for each definition by treatment group. Frequency counts and percentages of patients within each category will be provided for categorical data. Subject rates will be compared between treatment groups using the Chi-square test or Fisher's exact test. The modified Poisson regression will be used to estimate the RR and corresponding 95%CI with treatment group as the independent variable and the presence or absence of SICH as the dependent variable.[8] The treatment effect will be presented as the RR with the corresponding 95% CI.

Dose Expansion (Phase IIa):

The primary endpoint in phase IIa is the proportion of patients with mRS 0-1 at Day 90 and primarily analyzed in the FAS population in the phase IIa. The handling of

missing data for the primary endpoint (Proportion of patients with a mRS 0-1 at Day 90) is detailed in 6.2. As the primary endpoint, the mRS score 0-1 at Day 90.

Pooled analysis of phases Ib and IIa:

A prespecified supportive analysis that combines phase Ib and phase IIa will be performed. According to the Ib/IIa combined design, phase Ib patients from the two doses chosen to be used in phase IIa, could be added to the overall patients sample size of dose A and dose B arms of phase IIa patients, respectively. The mRS score of 0-1 at Day 90 will be analyzed using modified Poisson regression, from which risk ratio (RR) with its 95% CI will be estimated. Both adjusted and unadjusted RR and 95% CI will be reported.

6.5.3 Analysis of the Secondary Outcome

6.5.3.1 Dichotomized outcomes

Secondary efficacy outcomes encompass mRS 0-4, mRS 0-3, mRS 0-2 and mortality at 90 days. These outcomes will provide further insights into the therapeutic impact.

The therapeutic effect will be assessed in the intention-to-treat (ITT) population through a modified Poisson regression to calculate the risk ratio (RR) and its 95% CI. Should this model fail to converge, alternative GLMs will be sequentially tested until convergence is achieved: a GLM with a binomial distribution and log link function, a GLM with a negative binomial distribution and log-link function, and a GLM with a binomial distribution and logit-link function, where the odds ratio (OR) will be transformed into RR. Covariate adjusted RR will be calculated the adjusted analysis of the primary endpoint.

6.5.3.2 Changes in the NIHSS score 5-7 days after surgery or at discharge

The NIHSS is a standardized neurological assessment tool, scoring from 0 to 42, where higher scores denote greater severity, and will be analyzed using win ratio method. NIHSS will be summarized using numbers and proportion of wins across all possible pairs by treatment arm and analyzed using win ratio method since NIHSS has been empirically shown to be skewedly distributed. Both crude and adjusted win ratio via ITPW approach will be estimated using WINS package based on R. Participants with missing NIHSS scores at these times will be excluded. [9,10]

6.5.3.3 Quality of life measured with the EQ-5D-3L at Day 90.

The EQ-5D-3L is a health assessment tool that encompasses five dimensions: mobility, self-care, daily activities, pain/discomfort, and anxiety/depression. Each

dimension features three categories, ranging from no problem to extreme problems. The tool is designed for self-administration, with respondents rating their overall health on a 0-100 vertical visual analogue scale (EQ-VAS) on the day of the interview. The EQ-5D-3L will be completed 90 days post-enrollment. EQ-5D-3L will be summarized using numbers and proportion of wins across all possible pairs by treatment arm and analyzed using win ratio method since EQ-5D-3L has been empirically shown to be skewedly distributed. Both crude and adjusted win ratio (covariates are defined as Section 9.6) will be estimated using WINS package based on R. Subjects with missing observations for this endpoint will be excluded from the analysis.

6.5.3.4 Mortality at Day 90

All-cause mortality is the principal safety endpoint. This endpoint is assessed on a binary scale, where a positive result indicates death due to any cause, and a negative result indicates no death. Deaths occurring on or before the 90th (±14 days) calendar day from the date of randomization (day 0) are included in the count. Subjects who are alive at day 90 are censored at this point. Mortality rates are calculated as the number of deaths observed divided by the total number of subjects monitored over the 90-day study duration. A modified Poisson regression will be conducted, with treatment group as the independent variable and death at 90 days as the dependent variable. The effect of the treatment will be expressed as a RR along with the corresponding 95%CI. Furthermore, a proportional hazards regression model will be utilized to determine the hazard ratio with a 95% CI. Additionally, the Kaplan-Meier method will be used to generate survival curves stratified by treatment arm. A log-rank test will be performed to compare the survival curves between the two treatment groups.

6.5.3.5 Adverse Events

The occurrence and prevalence of adverse events (AEs) among participants in the TNK group and the control group will be documented according to the System Organ Class (SOC) of the Medical Dictionary for Regulatory Activities (MedDRA). AE data collection will cease on day 30, while serious adverse events (SAEs) will continue to be monitored until day 90 or the final study contact. Descriptive statistics will be utilized to present AE data at baseline hospitalization and subsequent follow-up appointments. AEs will be classified, and their incidence rates will be detailed by category and severity. Each participant will be accounted for only once per category, with the most severe AE within that category being considered. Comprehensive records of AEs observed during the study will be compiled for each participant, including the

investigator's direct report, category, onset and resolution dates, and the assessment of causality and severity. The timing of AE onset will be indicated in relation to the procedure day, measured in days. A detailed tabulation of SAEs will be compiled for each participant. Analysis will focus on categories that are reported by at least 3% of the patients.

7.Tables and Figures for the Main Paper

The proposed table and figures for the main results are described below:

- Table 1 will report the key baseline characteristics of participants by patient group.
- Table 2 will report the primary and secondary efficacy and safety outcomes.
- Figure 1 will be the CONSORT diagram.
- Figure 2 will report a proportional bar chart of Intracranial Hemorrhage within 24 Hours in Phase Ib and Distribution of Score on the Modified Rankin Scale at 90 Days in Phase IIa.

8. Changed to Planned Analyses

All modifications to the statistical analysis plan will be recorded in an updated statistical analysis plan or within the clinical study report.

9. Fundings

DATE trial is an investigator-initiated study which is organized by the first affiliated hospital of the Army Medical University (Southwest Hospital of Army Medical University) and conducted in about 30 comprehensive stroke centers in China. The project was supported by the Key Project of Chongqing Science Health Joint Medical Research Project, National Natural Science Foundation of China, the Major Project of Clinical Research Incubation at the First Affiliated Hospital of Army Medical University, Key Special Projects for Technological Innovation and Application Development in Chongqing and Chongqing Postdoctoral Program for Innovative Talent.

10. APPENDIX

Appendix table 1 Modified Rankin Scale

The modified Rankin Scale (mRS) is an ordinal hierarchical scale ranging from 0 to 6, with higher scores indicating more severe disability. A score of 6 has been added to signify death.

Category	Short description	Long description
0	No symptoms	No symptoms
1	Symptoms, no disability	Minor symptoms that do not interfere with lifestyle
2	Slight disability	Slight disability, symptoms that lead to some restriction in lifestyle, but do not interfere with the patient's capacity to look after himself.
3	Moderate disability	Moderate disability, symptoms that significantly restrict lifestyle and prevent totally independent existence
4	Moderately severe disability	Moderately severe disability, symptoms that clearly prevent independent existence though not needing constant attention
5	Severe disability	Severe disability, totally dependent patient requiring constant attention day and night.
6	Death	Death

Appendix table 2 Extended Treatment In Cerebral Ischemia (eTICI) Scale

eTICI grade	Description
0	No reperfusion of the target downstream territory
1	Contrast passes the occlusion but does not result in distal perfusion
2a	Partial reperfusion of<50% of the target downstream territory
2b50	Partial reperfusion of 50%-66% of the target downstream territory
2b67	Partial reperfusion of 67%-89% of the target downstream territory
2c	Partial reperfusion of 90%-99% of the target downstream territory
3	Complete reperfusion of the target downstream territory

Appendix table 3 National Institute of Health Stroke Scale

The NIHSS is an ordinal hierarchical scale to evaluate the severity of stroke by assessing a patient's performance. Scores range from 0 to 42, with higher scores indicating a more severe deficit. Administer stroke scale items in the order listed. Record performance in each category after each subscale exam. Do not go back and change scores. Follow directions provided for each exam technique. Scores should reflect what the patient does, not what the clinician thinks the patient can do. The clinician should record answers while administering the exam and work quickly. Except where indicated, the patient should not be coached (i.e. repeated requests to patient to make a special effort).

Instructions	Scale definition
1a. Level of consciousness. The investigator must choose a response if a full evaluation is prevented by such obstacles as an endotracheal tube, language barrier, orotracheal trauma/bandages. A 3 is scored only if the patient makes no movement (other than reflexive posturing) in response to noxious stimulation.	0 = Alert; keenly responsive.<br>1 = Not alert; but arousable by minor stimulation to obey, answer, or respond.<br>2 = Not alert; required repeated stimulation to attend, or is obtunded and requires strong or painful stimulation to make movements (not stereotyped).<br>3 = Responds only with reflex motor or autonomic effects or totally unresponsive, flaccid and areflexic.
1b. LOC Questions: The patient is asked the month and his/her age. The answer must be correct – there is not partial credit for being close. Phasic and stuporous patients who do not comprehend the questions will score 2. Patients unable to speak because of endotracheal intubation, orotracheal trauma, severe dysarthria from any cause, language barrier, or any other problem not secondary to aphasia are given a 1. It is important that only the initial answer be graded and that the examiners not "help" the patient with verbal or non-verbal clues.	0 = Answers both questions correctly.<br>1 = Answers one question correctly.<br>2 = Answers neither question correctly.
1c. LOC Commands: The patient is asked to open and close the eyes and then to grip and release the non-paretic hand. Substitute another one step command if the hand cannot be used. Credit is given if an unequivocal attempt is made but not completed due to weakness. If the patient does not respond to command, the task should be demonstrated to him or her (pantomime), and the result scored(i.e. follows none, one or two commands). Patients with trauma, amputation, or other physical impediments should be given suitable one-step commands. Only the first attempt is scored.	0 = Performs both tasks correctly.<br>1 = Performs one task correctly.<br>2 = Performs neither task correctly.

2. Best Gaze: Only horizontal eye movements will be tested. Voluntary or reflexive (oculocephalic) eye movements will be scored, but caloric testing is not done. If the patient has a conjugate deviation of the eyes that can be overcome by voluntary or eflexive activity, the score will be a 1. If a patient has an isolated peripheral nerve paresis (CN III, IV or VI), score a 1. Gaze is testable in all aphasic patients. Patients with ocular trauma, bandages, preexisting blindness, or other disorder of visual acuity or fields should be tested with reflexive movements, and a choice made by the investigator. Establishing eye contact and then moving about the patient from side to side will occasionally clarify the presence of a partial gaze palsy.	0= Normal.<br>1= Partial gaze palsy; gaze is abnormal in one or both eyes, but forced deviation or total gaze paresis is not present.<br>2= Forced deviation; or total gaze paresis not overcome by the oculocephalic maneuver.
3. Visual: Visual fields (upper and lower quadrants) are tested by confrontation, using finger counting or visual threat, as appropriate. Patients maybe encouraged, but if they look at the side of the moving finger appropriately, this can be scored as normal. If there is unilateral blindness or enucleation, visual fields in the remaining eye are scored. Score 1 only if a clear-cut asymmetry, including quadrantanopia, is found. If patient is blind from any cause, score 3. Double simultaneous stimulation is performed in this case. If there is extinction, the patient receives a 1, and the results are used to respond to item 11.	0= No visual loss.<br>1= Partial hemianopia.<br>2= Complete hemianopia.<br>3= Bilateral hemianopia (blind including cortical blindness)
4. Facial palsy: Ask or use pantomime to encourage the patient to show teeth or raise eyebrows and close eyes. Score symmetry of grimace in response to noxious stimuli in the poorly response or non-comprehending patient. If facial trauma/bandages, orotracheal tube, tape or other physical barriers obscure the face, these should be removed to the extent possible.	0 = Normal symmetrical movements.<br>1= Minor paralysis (flattened nasolabial fold, asymmetry on smiling)<br>2= Partial paralysis (total or near-total paralysis of lower face)<br>3= Complete paralysis of one or both sides (absence of facial movement in the upper and lower face).
5. Motor arm: The limb is placed in the appropriate position: extend the arms (palms down) 90 degrees (if sitting) or 45 degrees (if supine). Drift is scored if the arm falls before 10 seconds. The aphasic patient is encouraged using urgency in the voice and pantomime, but not noxious stimulation. Each limb is tested in turn, beginning with the non-paretic arm. Only in the case of amputation or joint fusion at the shoulder, the examiner should record the score asuntestable (UN), and clearly write the explanation for this choice.	0= No drift; limb holds 90 (or 45) degrees for full 10 seconds.<br>1= Drift; limb holds 90 (or 45) degrees, but drifts down before full 10 seconds; does not hit bed or other support.<br>2= Some effort against gravity; limb cannot get to or maintain (if cued) 90 (or 45) degrees,drifts down to bed, but has some effort against gravity.<br>3= No effort against gravity; limb falls.<br>4= No movement.<br>UN = Amputation or joint fusion: explain:<br>5a = Left Arm. 5b = Right arm.

6. Motor leg: The limb is placed in the appropriate position: hold the leg at 30 degrees (always tested supine). Drift is scored if the leg falls before 5 seconds. The aphasic patient is encouraged using urgency in the voice and pantomime, but not noxious stimulation. Each limb is tested in turn, beginning with the non-paretic leg. Only in the case of amputation or joint fusion at the hip, the examiner should record the score as untestable (UN), and clearly write the explanation for this choice.	0= No drift; leg holds 30-degree position for full 5 seconds.<br>1= Drift; leg falls by the end of the 5-second period but does not hit bed.<br>2= Some effort against gravity; leg falls to bed by 5 seconds, but has some effort against gravity.<br>3= No effort against gravity; leg falls to bed immediately.<br>4= No movement.<br>UN = Amputation or joint fusion: explain:<br>6a. Left Leg<br>6b. Right Leg.
7. Limb ataxia: This item is aimed at finding evidence of a unilateral cerebellar lesion. Test with eyes open. In case of visual defect, ensure testing is done in intact visual field. Thefinger-nose-finger and heel-shin tests are performed on both sides, and ataxia is scored only if present out of proportion to weakness. Ataxia is absent in the patient who cannot understand or is paralyzed. Only in the case of amputation or joint fusion, the examiner should record the score asuntestable (UN), and clearly write the explanation for this choice. In case of blindness, test by having the patient touch nose from extended arm position.	0= Absent.<br>1= Present in one limb.<br>2= Present in two limbs.<br>UN = Amputation or joint fusion: explain:
8. Sensory: Sensation or grimace to pinprick when tested, or withdrawal from noxious stimulus in the obtunded or aphasic patient. Only sensory loss attributed to stroke is scored as abnormal and the examiner should test as many body areas (arms [not hands], legs, trunk, face) as needed to accurately check for hemisensory loss. A score of 2, 'severe or total sensory loss', should only be given when a severe or total loss of sensation can be clearly demonstrated. Stuporous and aphasic patients will, therefore, probably score 1 or 0. The patient with brainstem stroke who has bilateral loss of sensation is scored 2. If the patient does not respond and is quadriplegic, score 2. Patients in a coma (item 1a=3) are automatically given a 2 on this item.	0= Normal; no sensory loss.<br>1= Mild-to-moderate sensory loss; patients feels pinprick is less sharp or is dull on the affected side; or there is a loss of superficial pain with pinprick, but patient is aware of being touched.<br>2= Severe to total sensory loss; patient is not aware of being touched in the face, arm and leg.
9. Best language: A great deal of information about comprehension will be obtained during the preceding sections of the examination. For this scale item, the patient is asked to describe what is happening in the attached picture, to name the items on the attached naming sheet and to read from the attached list of sentences. Comprehension is judged from responses here, as well as to all of the commands in the preceding general neurological exam. If visual loss interferes with the tests, ask the patient to identify objects placed in the hand, repeat, and produce speech. The intubated patient should be asked to write. The patient in a coma (item 1a=3) will	0= No aphasia; normal<br>1= Mild-to-moderate aphasia; some obvious loss of fluency or facility of comprehension, without significant limitation on ideas expressed or form of expression. Reduction of speech and/or comprehension, however, makes conservation about provided materials difficult or impossible. For example, in conversation about provided materials, examiner can identify picture or naming card content from patient's response.

automatically score 3 on this item. The examiner must choose a score for the patient with stupor or limited cooperation, but a score of 3 should be used only if the patient is mute and follows no one-step commands.	2= Severe aphasia; all communication is through fragmentary expression; great need for inference, questioning, and guessing by the listener. Range of information that can be exchanged is limited; listener carries burden of communication. Examiner cannot identify materials provided from patient response.<br>3 = Mute, global aphasia: no usable speech or auditory comprehension.
10. Dysarthria: If patient is thought to be normal, an adequate sample of speech must be obtained by asking patient to read or repeat words from the attached list. If the patient has severe aphasia, the clarity of articulation of spontaneous speech can be rated. Only if patient is intubated or has other physical barriers to producing speech, the examiner should record the score as untestable (UN), and clearly write an explanation for this choice. Do not tell the patient why he or she is being tested.	0= Normal.<br>1= Mild-to-moderate dysarthria; patient slurs at least some words and, at worst, can be understood by some difficulty.<br>2= Severe dysarthria: patient's speech is so slurred as to be unintelligible in the absence of or out of proportion to any dysphasia, or is mute/anarthric.<br>UN = Intubated or other physical barrier.
11. Extinction and Inattention (formerly Neglect): Sufficient information to identify neglect maybe obtained during the prior testing. If the patient has a severe visual loss preventing visual double simultaneous stimulation, and the cutaneous stimuli are normal, the score is normal. If the patient has aphasia but does appear to attend to both sides, the score is normal. The presence of visual spatial neglect or anosagnosia may also betaken as evidence of abnormality. Since the abnormality is scored only if present, the item is never untestable.	0= No abnormality.<br>1= Visual, tactile, auditory, spatial, or personal inattention or extinction to bilateral simultaneous stimulation in one of the sensorymodalities.<br>2= Profound hemi-inattention or extinction to more than one modality; does not recognize own hand or orients to only one side of space.

Chinese Version

项目	评分标准
1a.意识水平: 即使不能全面评价(如气管插管、语言障碍、气管 创伤及绷带包扎等)，检查者也必须选择 1 个反应。只在患者对有害刺激无反应时(不是反射)才能记录 3 分。	0 清醒，反应灵敏<br>1 嗜睡，轻微刺激能唤醒，可回答问题，执行指令<br>2 昏睡或反应迟钝，需反复刺激、强烈或疼痛刺激才 有非刻板的反应<br>3 昏迷，仅有反射性活动或自发性反应或完全无反应、 软瘫、无反射
1b.意识水平提问: 月份、年龄。仅对初次回答评分。失语和昏迷者不能理解问题记 2 分，因气管插管、气管创伤、严重构 音障碍、语言障碍或其他任何原因不能完成者(非失语 所致)记 1 分。可书面回答。	0 两项均正确<br>1 一项正确<br>2 两项均不正确
1c.意识水平指令: 睁闭眼;非瘫痪侧握拳松开。仅对最初反应评 分，有明确努力但未完成的也给分。若对指令无反应，用动作示意，然后记录评分。对创伤、截肢或其他生理缺陷者，应予适当的指令。	0 两项均正确<br>1 一项正确<br>2 两项均不正确
2.凝视: 只测试水平眼球运动。对随意或反射性眼球运 动记分。若眼球偏斜能被随意或反射性活动纠正，记 1 分。若为孤立的周围性眼肌麻痹记 1 分。对失语者，凝 视是可以测试的。对眼球创伤、绷带包扎、盲人或有其 他视力、视野障碍者，由检查者选择一种反射性运动来 测试，确定眼球的联系，然后从一侧向另一侧运动，偶 尔能发现部分性凝视麻痹。	0 正常<br>1 部分凝视麻痹(单眼或双眼凝视异常，但无强迫凝 视或完全凝视麻痹)<br>2 强迫凝视或完全凝视麻痹(不能被头眼反射克服)
3.视野: 若能看到侧面的手指，记录正常，若单眼盲或眼球<br>摘除，检查另一只眼。明确的非对称盲(包括象限盲)， 记 1 分。若全盲(任何原因)记 3 分。若频临死亡记 1 分，结果用于回答问题 11。	0 无视野缺损<br>1 部分偏盲<br>2 完全偏盲<br>3 双侧偏盲(包括皮质盲)

4.面瘫:	0 正常<br>1 轻微(微笑时鼻唇沟变平、不对称)<br>2 部分(下面部完全或几乎完全瘫痪)<br>3 完全(单或双侧瘫痪，上下面部缺乏运动)
5、6.上下肢运动: 置肢体于合适的位置:坐位时上肢平举 90°， 仰卧时上抬 45°，掌心向下，下肢卧位抬高 30°，若上肢 在 10 秒内，下落在 5 秒内下落，记 1~4 分。对失语者 用语言或动作鼓励，不用有害刺激。依次检查每个肢体， 从非瘫痪侧上肢开始。	上肢:<br>0 无下落，置肢体于 90° (或 45°)坚持 10 秒<br>1 能抬起但不能坚持 10 秒，下落时不撞击床或其他支持物<br>2 试图抵抗重力，但不能维持坐位 90° 或仰位 45°<br>3 不能抵抗重力，肢体快速下落<br>4 无运动<br>9 截肢或关节融合，解释:<br>5a 左上肢;5b 右上肢
	下肢:<br>0 无下落，于要求位置坚持 5 秒<br>1 5 秒末下落，不撞击床<br>2 5 秒内下落到床上，可部分抵抗重力<br>3 立即下落到床上，不能抵抗重力<br>4 无运动<br>9 截肢或关节融合，解释:<br>6a 左下肢; 6b 右下肢
7.肢体共济失调: 目的是发现一侧小脑病变。检查时睁眼，若有 视力障碍，应确保检查在无视野缺损中进行。进行双侧 指鼻试验、跟膝径试验，共济失调与无力明显不呈比例 时记分。若患者不能理解或肢体瘫痪不记分。盲人用伸 展的上肢摸鼻。若为截肢或关节融合记 9 分，并解释。	0 无共济失调<br>1 一个肢体有<br>2 两个肢体有，共济失调在:<br>右上肢 1=有，2=无<br>9 截肢或关节融合，解释: 左上肢 1=有，2=无<br>9 截肢或关节融合，解释:<br>右上肢 1=有，2=无<br>9 截肢或关节融合，解释: 左下肢 1=有，2=无<br>9 截肢或关节融合，解释:<br>右下肢 1=有，2=无
8.感觉:<br>检查对针刺的感觉和表情，或意识障碍及 失语者对有害刺激的躲避。只对与脑卒中有关的感 觉缺失评分。偏身感觉丧失者需要精确检查，应测试身 体多处[上肢(不包括手)、下肢、躯干、面部]确定有 无偏身感觉缺失。严重或完全的感觉缺失记 2 分。昏睡 或失语者记 1 或 0 分。脑干卒中双侧感觉缺失记 2 分。 无反应或四肢瘫痪者记 2 分。昏迷患者(1a=3)记 2 分。	0 正常<br>1 轻-中度感觉障碍，(患者感觉针刺不尖锐或迟钝，<br>或针刺感缺失但有触觉)<br>2 重度-完全感觉缺失(面、上肢、下肢无触觉)
9. 语言: 命名、阅读测试。若视觉缺损干扰测试，可让 患者识别放在手上的物品，重复和发音。气管插管者手 写回答。昏迷者记 3 分。给恍惚或不合作者选择一个记 分，但 3 分仅给不能说话且不能执行任何指令者。	0 正常<br>1 轻-中度失语:流利程度和理解能力部分下降，但表 达无明显受限<br>2 严重失语，交流是通过患者破碎的语言表达，听者须推理、询问、猜测，交流困难<br>3 不能说话或者完全失语，无言语或听力理解能力
10.构音障碍: 读或重复表上的单词。若有严重的失语，评估 自发语言时发音的清晰度。若因气管插管或其他物理障 碍不能讲话，记 9 分。同时注明原因。不要告诉患者为 什么做测试。	0 正常<br>1 轻-中度，至少有些发音不清，虽有困难但能被理解 2 言语不清，不能被理解，但无失语或 与失语不成<br>比例，或失音<br>9 气管插管或其他物理障碍，解释:
11.忽视: 若患者严重视觉缺失影响双侧视觉的同时检查，皮 肤刺激正常，记为正常。若失语，但确实表现为对双侧 的注意，记分正常。视空间忽视或疾病失认也可认为是 异常的证据。	0 正常<br>1 视、触、听、空间觉或个人的忽视;或对一种感觉 的双侧同时刺激忽视<br>2 严重的偏侧忽视或一种以上的偏侧忽视;不认识自 己的手;只能对一侧空间定位

Appendix table 4 EUROQOL-5D-3L

The EuroQoL 5-dimensions 3-level (EQ-5D-3L) questionnaire is a standardized measure of health outcome that has been used extensively in patients with stroke.

Under each heading, please tick the ONE box that best describes your health today.

Mobility

I have no problems in walking about □

I have some problems in walking about □

I am confined to bed □

Self-care

I have no problems with self-care □

I have some problems washing or dressing myself □

I am unable to wash or dress myself □

Usual activities (e.g. work, study, housework, family or leisure activities)

I have no problems with performing my usual activities □

I have some problems with performing my usual activities □

I am unable to perform my usual activities □

Pain/discomfort

I have no pain or discomfort □

I have moderate pain or discomfort □

I have extreme pain or discomfort □

Anxiety/depression

I am not anxious or depressed □

I am moderately anxious or depressed □

I am extremely anxious or depressed □

Appendix Table 4 EUROQOL 5D-3L

- We would like to know how good or bad your health is TODAY.
- This scale is numbered from 0 to 100.
- 100 means the best health you can imagine.
  0 means the worst health you can imagine.
- Mark an X on the scale to indicate how your health is TODAY.
- Now, please write the number you marked on the scale in the box below.

YOUR HEALTH TODAY =

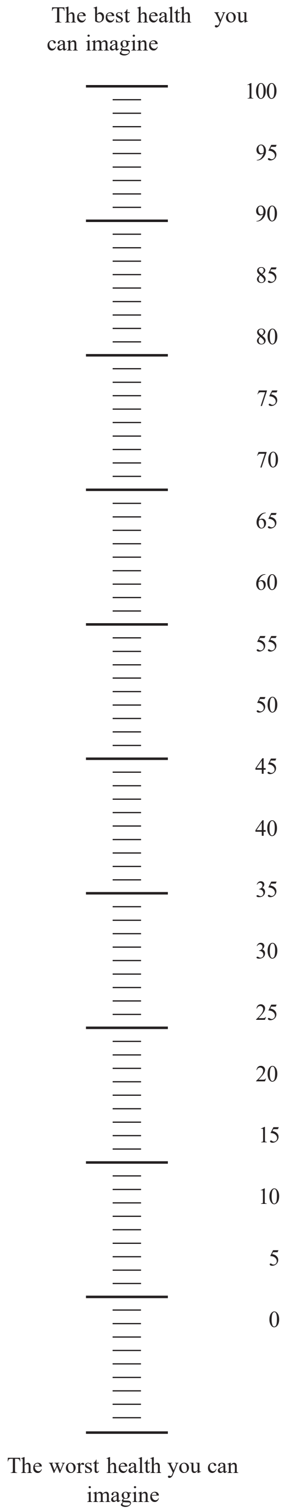

In previous versions of the EQ-5D-3L, the numerical scale straddled the EQ VAS (provided in the annex for reference). Users are encouraged to use the latest version of the EQ-5D-3L in new studies.

Reference:

1. CPMP/ICH/363/96. ICH E9 Statistical Principles for Clinical Trials.
2. Association AS. Ethical Guidelines for Statistical Practice. Prepared by the Committee on Professional Ethics.
3. Society RS. The Royal Statistical Society: Code of Conduct.
4. Zhang X, Xie Y, Wang H, et al. Symptomatic Intracranial Hemorrhage After Mechanical Thrombectomy in Chinese Ischemic Stroke Patients: The ASIAN Score. *Stroke.* 2020;51(9):2690-2696.
5. Huang JH, Su QM, Yang J, et al. Sample sizes in dosage investigational clinical trials: a systematic evaluation. *Drug design, development and therapy.* 2015;9:305-312.
6. Yuan Y, Hess KR, Hilsenbeck SG, Gilbert MR. Bayesian Optimal Interval Design: A Simple and Well-Performing Design for Phase I Oncology Trials. *Clinical cancer research : an official journal of the American Association for Cancer Research.* 2016;22(17):4291-4301.
7. Hernández-Jiménez M, Abad-Santos F, Cotgreave I, et al. Safety and Efficacy of ApTOLL in Patients With Ischemic Stroke Undergoing Endovascular Treatment: A Phase 1/2 Randomized Clinical Trial. *JAMA neurology.* 2023;80(8):779-788.
8. Zou G. A modified poisson regression approach to prospective studies with binary data. *Am J Epidemiol.* 2004;159(7):702-706.
9. Pocock SJ, Ariti CA, Collier TJ, Wang D. The win ratio: a new approach to the analysis of composite endpoints in clinical trials based on clinical priorities. *Eur Heart J.* 2012;33(2):176-182.
10. Duolao Wang SZ, Ying Cui, Nengjie He, Tao Chen and Bo Huang. Adjusted win ratio using the inverse probability of treatment weighting (IPTW). (drafting).
