# Supplementary material for: Intra-Arterial Tenecteplase After Successful Reperfusion in Large Vessel Occlusion Stroke: A Randomized Clinical Trial
Source: JAMA Neurol. 2025 Jul 5;82(9):895–904. doi: 10.1001/jamaneurol.2025.2036 (PMC12228979; doi:10.1001/jamaneurol.2025.2036)
Supplement: Supplement 3. — eAppendix. List of Investigators eMethods 1. Study Design of Phase 1b Dose Escalation Trial eMethods 2. Detailed Inclusion and Exclusion Criteria eMethods 3. Definition of Analyzed Population eMethods 4. Characteristics of EQ-5D-3L Value Sets in China eTable 1. Characteristics of the Phase 1b Population by Group eTable 2. Characteristics of the Pooled Analysis eTable 3. Pooled Analysis of Study Outcomes eTable 4. Summary of Serious Adverse Events Reported by Local Investigators of the Phase 1b (Safety Population) eTable 5. Summary of Adverse Events Reported by Local Investigators of the Phase 1b (Safety Population) eTable 6. Summary of Serious Adverse Events Reported by Local Investigators of the Phase 2a (Safety Population) eTable 7. Summary of Adverse Events Reported by Local Investigators of the Phase 2a (Safety Population) eFigure 1. Distribution of 30 Enrollment Center in China eFigure 2. Distribution of the Modified Rankin Scale Score at 90 Days (Phase 1b) eFigure 3. Distribution of the Modified Rankin Scale Score at 90 Days (Phase 1b+2a) [file jamaneurol-e252036-s003.pdf]

Supplementary Online Content

Hou X, Huang J, Wang L, et al. Intra-arterial tenecteplase after successful reperfusion in large vessel occlusion stroke: a randomized clinical trial. *JAMA Neurol*. Published online July 5, 2025. doi:10.1001/jamaneurol.2025.2036

eAppendix. List of Investigators

eMethods 1. Study Design of Phase 1b Dose Escalation Trial

eMethods 2. Detailed Inclusion and Exclusion Criteria

eMethods 3. Definition of Analyzed Population

eMethods 4. Characteristics of EQ-5D-3L Value Sets in China

eTable 1. Characteristics of the Phase 1b Population by Group

eTable 2. Characteristics of the Pooled Analysis

eTable 3. Pooled Analysis of Study Outcomes

eTable 4. Summary of Serious Adverse Events Reported by Local Investigators of the Phase 1b (Safety Population)

eTable 5. Summary of Adverse Events Reported by Local Investigators of the Phase 1b (Safety Population)

eTable 6. Summary of Serious Adverse Events Reported by Local Investigators of the Phase 2a (Safety Population)

eTable 7. Summary of Adverse Events Reported by Local Investigators of the Phase 2a (Safety Population)

eFigure 1. Distribution of 30 Enrollment Center in China

eFigure 2. Distribution of the Modified Rankin Scale Score at 90 Days (Phase 1b)

eFigure 3. Distribution of the Modified Rankin Scale Score at 90 Days (Phase 1b+2a)

This supplementary material has been provided by the authors to give readers additional information about their work.

© 2025 Hou X et al. *JAMA Neurology*.

eAppendix. List of Investigators

Enrolling Clinical Centers (number of patients enrolled), Principal Investigators

Phase 1b (Dose Escalation)

Jingzhou Central Hospital (14), Junfeng Su

The First Affiliated Hospital (Southwest Hospital) of the Army Medical University (11), Zhenhua Zhou

Zigong Third People's Hospital (9), Li Wang;

Three Gorges Hospital affiliated to Chongqing University (6), Shengli Chen

Ya'an People's Hospital (4), Jian Wang

Chengdu Second People's Hospital (3), Changchuan Wu

Chongqing Hechuan District People's Hospital (1), Zicheng Hu

Phase 2a (Dose Expansion)

Zigong Third People's Hospital (23), Li Wang;

The First Affiliated Hospital of University of South China (15), Lin Zeng

The People's Hospital of QianNan (14), Maojun Jiang;

Qujing First People's Hospital (13), Boyu Chen;

Gulin People's Hospital (13), Xiangping Cheng

Jingzhou Central Hospital (12), Junfeng Su

The First Affiliated Hospital (Southwest Hospital) of the Army Medical University (10), Zhenhua Zhou

Guilin People's Hospital (10), Honghua Pan

JiuJiang No.1 People's Hospital (9), Shen Xiaoping

Chongzhou People's Hospital (8), Youlin Wu

Three Gorges Hospital affiliated to Chongqing University (5), Shengli Chen

Affiliated Hospital of Youjiang Medical University for Nationalities (5), Xionglin Tang

Guangyuan Central Hospital (3), Tianqiang Pu

People's Hospital of Dali Prefecture (3), Shibo Han

© 2025 Hou X et al. *JAMA Neurology*.

Chongqing Changshou District People's Hospital (2), Lunxue Qu

Qianjiang Central Hospital of Chongqing (2), Yu Li

Wuhan Puren Hospital (2), Fengguang Li

The Third People's Hospital of Chengdu (2), Hua Liu

The People's Hospital of Jianyang (2), Zhong Fu

Dazhou Central Hospital (1), Haochun Zhang

Zhongnan Hospital of Wuhan University (1), Bin Mei

The Affiliated Hospital of Xuzhou Medical University (1), Yanbo Cheng

Ya'an People's Hospital (1), Jian Wang

© 2025 Hou X et al. *JAMA Neurology*.

eMethods

eMethods 1. Study Design of Phase 1b dose-escalation trial

The Endovascular Treatment for Acute Anterior Circulation Ischemic Stroke registry in China showed that the incidence of sICH within 24 hours after EVT was 13.8%. Based on this data, we designed a 14+8 enrollment plan as follows:

1) 14 subjects will be included in the trial for the first time for each dose;

2) If<2 DLT (dose limit toxicity) symptomatic intracranial hemorrhages occur within 24 hours after EVT, the trial advances to the next dose level;

3) If 2/14 DLT, then include 8 more people at the same dose;

4) If 2/14+0-1/8DLT (9.1% to 13.6%), proceed to the next dose level;

5) If 2/14+2/8 DLT (18.2%) or 3/14 DLT, then this dose is deemed unsafe and the prior lower dose is the MTD.

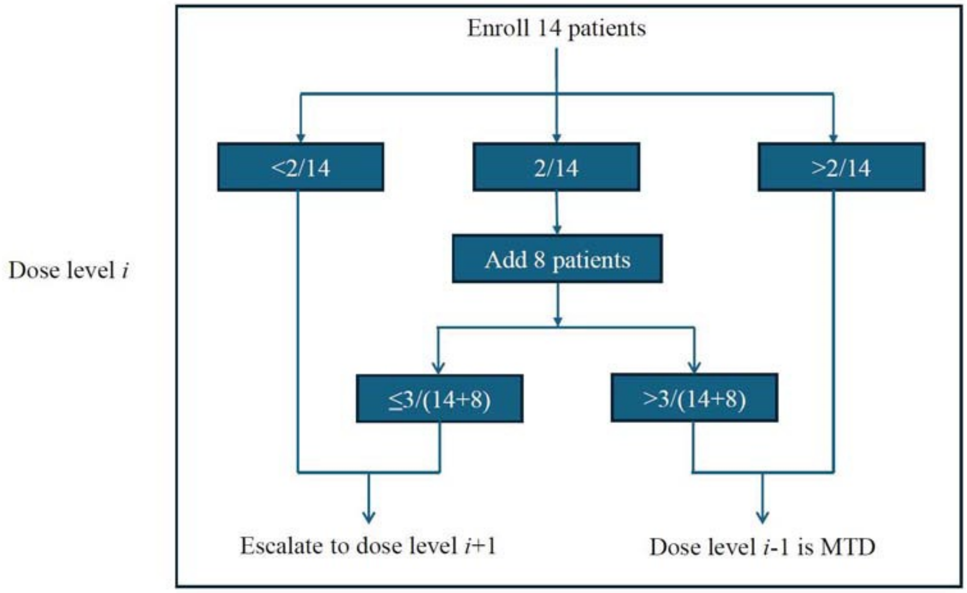

Trial Design of Phase 1b

© 2025 Hou X et al. *JAMA Neurology*.

eMethods 2. Detailed Inclusion and Exclusion Criteria

Inclusion criteria

- Age ≥18 years;
- Time from last known well within 24 hours;
- Large vessel occlusive stroke in the anterior circulation confirmed by CTA/MRA (Including intracranial segment of internal carotid artery and M1 or M2 segment of middle cerebral artery) and the vessel responsible for the signs and symptoms of acute ischemic stroke;
- ASPECTS score ≥6 based on NCCT;
- NIHSS score ≥6;
- Successful endovascular thrombectomy (eTICI 2b-3);
- Total pass numbers of thrombectomy procedure ≤3;
- Written informed consent signed by patients or their family members.

Exclusion Criteria:

Patients meeting any of the following criteria were be excluded from study enrolment.

- NIHSS score ≥25;
- Intracranial hemorrhage confirmed by cranial computed tomography (CT) or magnetic resonance imaging (MRI);
- Treated by intravenous thrombolysis;
- Prestroke mRS score ≥2;
- Intraoperative DSA angiography suggests vessel penetration, dissection, or extravasation of contrast medium;
- Pregnant or lactating patients;
- Allergic to contrast agents or tenecteplase;
- Systolic pressure greater than 185 mmHg or diastolic pressure greater than 110 mmHg after aggressive treatment;
- Genetic or acquired bleeding disposition with anticoagulation factor deficiency or

© 2025 Hou X et al. *JAMA Neurology*.

already taking oral anticoagulants within 48 hours and INR > 1.7;

- Blood glucose < 2.8 mmol/L (50 mg/dl) or > 22.2 mmol/L (400 mg/dl), platelets < 90*10'9/L;
- History of bleeding in the last 1 month (gastrointestinal and urinary tract bleeding);
- Patients on chronic hemodialysis and severe renal insufficiency (glomerular filtration rate < 30 ml/min or blood creatinine > 220 μmol/L (2.5mg/dl));
- Any terminal illness with a life expectancy of less than 6 months;
- Intracranial aneurysm, arteriovenous malformation;
- Brain tumors with occupying effect on imaging;
- Puncture to recanalization time > 90 min;
- Current participation in another clinical trial;
- Unlikely to be available for 90-day follow-up.

© 2025 Hou X et al. *JAMA Neurology*.

eMethods 3. Definition of Analyzed Population

Primary Analysis Set as Randomized

The primary analysis set as randomized includes all patients randomized into the trial except 1 patient who withdrew consent immediately after randomization. The analysis of the primary outcome was a complete case analysis of the primary analysis set as randomized which patients missing day 90 assessments were censored (deleted).

Definition of the Per-Protocol (PP) Population

The PP population is defined as the subset of the Primary Analysis Set as Randomized population excluding patients with major protocol violations.

The PP population includes patients who received the assigned treatment and do not have major protocol violations or deviations. Major protocol violations or deviations were identified in a blinded fashion prior to database lock. Patients with any of the following criteria were excluded from the PP population. These deviations were determined based on the medical monitors' records, using the following criteria at a minimum:

- Received but did not complete treatment with study drug, or dose of study drug administered outside recommended dose;

- Violated inclusion or exclusion criteria.

A list of patients to be excluded from the randomized patients to create the PP-Efficacy analysis was established and validated by the Steering Committee prior to unblinding.

Safety population

The Safety Population included all patients who received any study treatment. In case of violation of the randomization scheme, patients were be classified according to the

© 2025 Hou X et al. *JAMA Neurology*.

treatment they received. Patients were assigned to the different populations prior to unblinding of the database. Patients who withdraw informed consent immediately after randomization and did not receive any treatment should be excluded from Safety Populations.

© 2025 Hou X et al. *JAMA Neurology*.

eMethods 4. Characteristics of EQ-5D-3L Value Sets in China

Dimension	Mobility	Self-care	Usual activities	Pain/discomfort	Anxiety/depression
Grade					
None	0	0	0	0	0
Some/moderate	0.099	0.105	0.074	0.092	0.086
Confined to bed/unable to	0.246	0.208	0.193	0.236	0.205

© 2025 Hou X et al. *JAMA Neurology*.

eTables

eTable 1. Baseline Characteristics of the Phase 1b Population by Group

Characteristics	No. (%) 0.125 mg/kg of Tenecteplase (N=12)	0.0625 mg/kg of Tenecteplase (N=22)	0.03125 mg/kg of Tenecteplase (N=14)
Age, median (IQR), y	76 (68-81)	70 (60-79)	70 (60-73)
Sex [a]			
Female	8 (66.7)	11 (50.0)	5 (35.7)
Male	4 (33.3)	11 (50.0)	9 (64.3)
Medical history [b]			
Hypertension	7 (58.3)	12 (54.5)	10 (71.4)
Atrial fibrillation	7 (58.3)	9 (40.9)	8 (57.1)
Hyperlipidemia	5 (41.7)	6 (27.3)	2 (14.3)
Diabetes mellitus	3 (25.0)	4 (18.2)	4 (28.6)
Smoking [c]	4 (33.3)	7 (31.8)	6 (42.9)
Stroke	2 (16.7)	2 (9.1)	4 (28.6)
Prestroke modified Rankin Scale score [d]			
0	12 (100.0)	20 (90.9)	14 (100.0)
1	0 (0.0)	2 (9.1)	0 (0.0)
Baseline NIHSS score, median (IQR) [e]	17 (13-21)	18 (14-20)	16 (12-18)
Baseline ASPECTS, median (IQR) [f]	8 (7-10)	8 (7-9)	8 (7-9)
Systolic blood pressure at hospital arrival median (IQR), mm Hg	136 (126-163)	149 (128-158)	143 (132-155)
Blood glucose level at hospital arrival median (IQR), mmol/L [g]	6.8 (6.0-7.7) [N = 9]	7.4 (6.3-9.4) [N = 17]	6.7 (5.9-8.7) [N = 12]
Occlusion site			
Internal carotid artery	5 (41.7)	8 (36.4)	4 (28.6)
M1 segment	7 (58.3)	12 (54.5)	8 (57.1)
M2 segment	0 (0.0)	2 (9.1)	2 (14.3)
Angiographic eTICI scores before randomization [h]			
2b	3 (25.0)	4 (18.2)	6 (42.9)
2c	2 (16.7)	4 (18.2)	1 (7.1)
3	7 (58.3)	14 (63.6)	7 (50.0)
Time from last known well, median (IQR), min			
To puncture	294 (213-720)	314 (202-435)	227 (179-546)
To enrollment	372 (271-791)	346 (269-479)	268 (210-638)
To study treatment [i]	379 (275-795)	351 (271-485)	273 (220-642)
**Primary outcome**			
Symptomatic intracranial hemorrhage within 24h	3 (25.0)	2 (9.1)	1 (7.1)
**Secondary outcomes**			
mRS score of 0 to 1 at 90 days	3 (25.0)	9 (40.9)	6 (42.9)
mRS score of 0 to 2 at 90 days	5 (41.7)	11 (50.0)	8 (57.1)
mRS score at 90 days, median (IQR)	4 (2-6)	3 (1-5)	2 (1-3)
Improved angiographic eTICI score	2 (16.7)	1 (4.5)	1 (7.1)
Change of NIHSS score at 5-7 days or discharge if earlier, from baseline, median (IQR)	-3 (-9 to 1)	-6 (-10 to -2)	-11 (-17 to -8)
EQ-5D-3L score at 90 days, median (IQR)	0.3 (-0.1 to 1.0)	0.6 (-0.1 to 0.9)	0.9 (0.5 to 1.0)
Death within 90 days	4 (33.3)	5 (22.7)	2 (14.3)
Any radiologic intracranial hemorrhage within 24h	7 (58.3)	10 (45.5)	3 (21.4)

© 2025 Hou X et al. *JAMA Neurology*.

Abbreviation: ASPECTS, Alberta Stroke Program Early CT Score; NIHSS, National Institutes of Health Stroke Scale; eTICI, the expanded Thrombolysis in Cerebral Infarction.

SI conversion factor: To convert glucose to mg/dL, divide by 0.0555.

[a] Sex reported by the patient and verified by identification card.

[b] Comorbidities based on family or patient report.

[c] Current or within the prior 5 years.

[d] Scores on the mRS of functional disability range from 0 (no symptoms) to 6 (death).

[e] Scores on the National Institutes of Health Stroke Scale (NIHSS) range from 0 to 42, with higher scores indicating more severe neurological deficits.

[f] The Alberta Stroke Program Early Computed Tomography Score (ASPECTS) is an imaging measure of the extent of ischemic stroke. Scores range from 0 to 10, with higher scores indicating a smaller infarct core. Listed are values for the core laboratory assessment.

[g] Data on glucose at baseline were missing for 3 patients in the intra-arterial tenecteplase of 0.125 mg/kg group, 5 patients in the intra-arterial tenecteplase of 0.0625 mg/kg group, and 2 patients in the intra-arterial tenecteplase of 0.03125 mg/kg group.

[h] The expanded Thrombolysis in Cerebral Infarction (eTICI) scale is a reperfusion measure based on digital subtraction angiography, which ranges from 0 (no reperfusion) to 3 (complete reperfusion).

[i] Study treatment refers to the application of intra-arterial tenecteplase therapy.

© 2025 Hou X et al. *JAMA Neurology*.

eTable 2. Baseline Characteristics of the Pooled Analysis

Characteristics	No. (%) 0.0625 mg/kg of Tenecteplase (n=68)	0.03125 mg/kg of Tenecteplase (n=60)	Control (n=65)
Age, median (IQR), y	71 (61-77)	71 (60-75)	71 (56-78)
Sex [a]			
Female	28 (41.2)	21 (35.0)	35 (53.8)
Male	40 (58.8)	39 (65.0)	30 (46.2)
Medical history [b]			
Hypertension	38 (55.9)	41 (68.3)	40 (61.5)
Atrial fibrillation	27 (39.7)	28 (46.7)	36 (55.4)
Hyperlipidemia	22 (32.4)	16 (26.7)	19 (29.2)
Diabetes mellitus	19 (27.9)	15 (25.0)	15 (23.1)
Smoking [c]	22 (32.4)	22 (36.7)	14 (21.5)
Stroke	13 (19.1)	11 (18.3)	13 (20.0)
Prestroke modified Rankin Scale score [d]			
0	61 (89.7)	57 (95.0)	62 (95.4)
1	7 (10.3)	3 (5.0)	3 (4.6)
Baseline NIHSS score, median (IQR) [e]	17 (13-20)	17 (11-20)	17 (12-20)
Baseline ASPECTS, median (IQR) [f]	8 (6-9)	9 (8-9)	8 (7-9)
Systolic blood pressure at hospital arrival median (IQR), mm Hg	143 (127-160)	144 (128-165)	138 (127-155)
Blood glucose level at hospital arrival median (IQR), mmol/L [g]	7.1 (6.0-9.0) [N = 56]	7.1 (5.9-8.1) [N = 52]	7.1 (5.9-8.2) [N = 53]
Occlusion site			
Internal carotid artery	16 (23.5)	9 (15.0)	25 (38.5)
M1 segment	45 (66.2)	41 (68.3)	35 (53.8)
M2 segment	7 (10.3)	10 (16.7)	5 (7.7)
Angiographic eTICI scores before enrollment [h]			
2b	15 (22.1)	18 (30.0)	17 (26.2)
2c	19 (27.9)	19 (31.7)	21 (32.3)
3	34 (50.0)	23 (38.3)	27 (41.5)
Time from last known well, median (IQR), min			
To puncture	326 (194-494)	316 (200-608)	322 (232-635)
To enrollment	387 (264-635)	362 (249-669)	384 (288-677)
To study treatment [i]	394 (267-640)	365 (257-674)	-

Abbreviation: ASPECTS, Alberta Stroke Program Early CT Score; NIHSS, National Institutes of Health Stroke Scale; eTICI, the expanded Thrombolysis in Cerebral Infarction.

SI conversion factor: To convert glucose to mg/dL, divide by 0.0555.

[a] Sex reported by the patient and verified by identification card.

[b] Comorbidities based on family or patient report.

[c] Current or within the prior 5 years.

[d] Scores on the mRS of functional disability range from 0 (no symptoms) to 6 (death).

[e] Scores on the National Institutes of Health Stroke Scale (NIHSS) range from 0 to 42, with higher scores indicating more severe neurological deficits.

[f] The Alberta Stroke Program Early Computed Tomography Score (ASPECTS) is an imaging measure of the extent of ischemic stroke. Scores range from 0 to 10, with higher scores indicating a smaller infarct core. Listed are values for the core laboratory assessment.

[g] Data on glucose at baseline were missing for 12 patients in the intra-arterial tenecteplase of 0.0625 mg/kg group, 8 patients in the intra-arterial tenecteplase of 0.03125 mg/kg group, and 12 patients in the control group.

[h] The expanded Thrombolysis in Cerebral Infarction (eTICI) scale is a reperfusion measure based on digital subtraction

© 2025 Hou X et al. *JAMA Neurology*.

angiography, which ranges from 0 (no reperfusion) to 3 (complete reperfusion).

[i] Study treatment refers to the application of intra-arterial tenecteplase therapy.

© 2025 Hou X et al. *JAMA Neurology*.

eTable 3. Pooled Analysis of Study Outcomes

					**Unadjusted Value**				**Adjusted Value** [a]			
**Outcome**	**0.0625 mg/kg of Tenecteplase (N=68)**	**0.03125 mg/kg of Tenecteplase (N=60)**	**Control (N=65)**	**Treatment effect metric**	**0.0625 mg/kg vs Control (95% CI)**	***p* Value**	**0.03125 mg/kg vs Control (95% CI)** [a]	***p* Value**	**0.0625 mg/kg vs Control (95% CI)**	***p* Value**	**0.03125 mg/kg vs Control (95% CI)** [a]	***p* Value**
**Primary outcome** [b]												
mRS score of 0 to 1 at 90 days	29 (42.6)	23 (38.3)	22 (33.8)	**RR**	1.26 (0.81 to 1.95)	0.30	1.13 (0.71 to 1.81)	0.60	1.21 (0.80 to 1.83)	0.37	0.96 (0.62 to 1.48)	0.85
**Secondary outcomes**												
mRS score of 0 to 2 at 90 days [b]	37 (54.4)	31 (51.7)	33 (50.8)	**RR**	1.07 (0.78 to 1.48)	0.76	1.02 (0.72 to 1.43)	0.92	1.10 (0.81 to 1.50)	0.54	0.97 (0.69 to 1.34)	0.83
mRS score at 90 days, median (IQR) [c, d]	2 (1 to 5)	2 (1 to 4)	2 (1 to 5)	**GenOR**	1.10 (0.70 to 1.74)	0.69	1.18 (0.73 to 1.88)	0.50	1.14 (0.71 to 1.82)	0.60	1.01 (0.62 to 1.65)	0.98
Improved angiographic reperfusion [e]	4 (5.9)	5 (8.3)	-		-	-	-	-	-	-	-	-
Change of NIHSS score at 5-7 days or discharge if earlier, from baseline, median (IQR) [f, g]	-6 (-12 to 0)	-7 (-12 to -4)	-6 (-13 to 0)	**WR**	0.93 (0.62 to 1.39)	0.71	1.15 (0.76 to 1.75)	0.51	0.95 (0.63 to 1.44)	0.81	1.19 (0.77 to 1.85)	0.42
EQ-5D-3L score at 90 days, median (IQR) [h, g]	0.6 (-0.1 to 1.0)	0.7 (0.1 to 1.0)	0.6 (-0.1 to 1.0)	**WR**	0.99 (0.62 to 1.57)	0.96	1.28 (0.80 to 2.05)	0.30	1.01 (0.63 to 1.61)	0.97	1.11 (0.68 to 1.82)	0.67
**Primary safety outcomes**												
Death within 90 days	14 (20.6)	9 (15.0)	14 (21.5)	**RR**	0.96 (0.50 to 1.85)	0.89	0.70 (0.33 to 1.49)	0.35	0.87 (0.46 to 1.65)	0.67	0.78 (0.37 to 1.66)	0.52
Symptomatic intracranial hemorrhage within 24h [i]	5 (7.4)	3 (5.0)	2 (3.1)	**RR**	2.39 (0.48 to 11.89)	0.29	1.63 (0.28 to 9.39)	0.59	-	-	-	-
**Secondary safety outcomes**												
Any radiologic intracranial hemorrhage within 24h [i]	23 (33.8)	16 (26.7)	18 (27.7)	**RR**	1.22 (0.73 to 2.04)	0.45	0.96 (0.54 to 1.71)	0.90	1.22 (0.74 to 2.02)	0.44	1.10 (0.63 to 1.93)	0.73
Systemic Bleeding [j, k]						0.54		0.78				
Mild	9 (13.2)	11 (18.3)	12 (18.5)									
Moderate	1 (1.5)	0 (0.0)	1 (1.5)									
Severe	29 (42.6)	17 (28.3)	20 (30.8)									

© 2025 Hou X et al. *JAMA Neurology*.

Abbreviations: mRS, modified Rankin scale; NIHSS, National Institutes of Health Stroke Scale; EQ-5D-3L, European Quality of Life Five-Dimension Three-Level scale. RR, risk ratio; GenOR, generalized odds ratio; WR, win ratio.

[a] Adjusted values were adjusted for age, baseline NIHSS score, baseline ASPECTS score, occlusion site, and time from last known well to randomization. The GenOR and win ratio were adjusted using the inverse probability treatment weighting method.

[b] RR was calculated using the modified Poisson regression model

[c] The mRS of functional disability ranges from 0 (no symptoms) to 6 (death).

[d] GenOR was calculated by the number of wins in the dose group over the control group divided by the number of wins in the control group over the dose group among all possible pairs of mRS scores, taking 1 patient from the dose group and 1 patient from the control group. For the analysis of mRS at 90 days, the GenOR value >1 indicated a favorable shift in mRS score in the dose group relative to the control group.

[e] The improvement angiographic reperfusion was defined as change of pre-intra-arterial tenecteplase eTICI 2b to eTICI 2c or eTICI 3, or pre-eTICI 2c to eTICI 3 after intra-arterial tenecteplase therapy. Statistical analysis was not applicable due to insufficient number of patients with improved angiographic reperfusion.

[f] Scores on the NIHSS range from 0 to 42, with higher values reflecting more severe neurologic impairment.

[g] WR was calculated by the number of wins in the dose group over the control group in outcome divided by the number of wins in the control group over the dose group among all possible pairs, taking 1 patient from the dose group and 1 patient from the control group.

[h] EQ-5D-3L is a continuous scale measure of self-reported quality of life. Scores range from -0.149 to 1, with lower scores indicating a worse quality of life.

[i] Symptomatic intracranial hemorrhage was defined according to the ECASS III standard.

[j] Bleeding events were defined according to the Global Utilization of Streptokinase and Tissue Plasminogen Activator for Occluded Coronary Arteries criteria as follows: severe bleeding was defined as fatal or intracranial hemorrhage or other hemorrhage causing hemodynamic compromise that required blood or fluid replacement, inotropic support, or surgical intervention; moderate bleeding as bleeding that required transfusion of blood but did not lead to hemodynamic compromise requiring intervention; and mild bleeding as bleeding not requiring transfusion and not causing hemodynamic compromise (e.g., subcutaneous bleeding, mild hematomas, and oozing from puncture sites).

[k] Chi-square Test.

© 2025 Hou X et al. *JAMA Neurology*.

eTable 4. Summary of Serious Adverse Events Reported by Local Investigators of the Phase 1b (Safety Population)

Event Description	0.125 mg/kg of Tenecteplase (N=12)	0.0625 mg/kg of Tenecteplase (N=22)	0.03125 mg/kg of Tenecteplase (N=14)	Total (N=48)
Total Events	11	9	3	23
Cardiac disorders	1	1	1	3
Infections and infestations	2	1	0	3
Injury, poisoning and procedural complications	0	2	0	2
Nervous system disorders	8	4	2	14
Respiratory, thoracic and mediastinal disorders	0	1	0	1

© 2025 Hou X et al. *JAMA Neurology*.

eTable 5. Summary of Adverse Events Reported by Local Investigators of the Phase 1b (Safety Population)

Event Description	0.125 mg/kg of Tenecteplase (N=12)	0.0625 mg/kg of Tenecteplase (N=22)	0.03125 mg/kg of Tenecteplase (N=14)	Total (N=48)
Total Events	38	41	14	93
Blood and lymphatic system disorders	2	1	0	3
Cardiac disorders	1	1	1	3
Endocrine disorders	0	1	0	1
Gastrointestinal disorders	3	1	2	6
Infections and infestations	9	12	4	25
Injury, poisoning and procedural complications	1	2	1	4
Metabolism and nutrition disorders	1	2	1	4
Nervous system disorders	12	13	4	29
Renal and urinary disorders	2	4	0	6
Respiratory, thoracic and mediastinal disorders	0	1	0	1
Skin and subcutaneous tissue disorders	1	0	0	1
Vascular disorders	6	3	1	10

© 2025 Hou X et al. *JAMA Neurology*.

eTable 6. Summary of Serious Adverse Events Reported by Local Investigators of the Phase 2a (Safety Population)

Event Description	0.0625 mg/kg of Tenecteplase (N=46)	0.03125 mg/kg of Tenecteplase (N=46)	Control (N=65)	Total (N=157)
Total Events	20	15	28	63
Blood and lymphatic system disorders	0	1	0	1
Cardiac disorders	5	5	9	19
Eye disorders	0	1	0	1
General disorders and administration site conditions	1	1	0	2
Infections and infestations	2	1	2	5
Injury, poisoning and procedural complications	3	0	5	8
Nervous system disorders	3	3	8	14
Respiratory, thoracic and mediastinal disorders	6	3	3	12
Vascular disorders	0	0	1	1

© 2025 Hou X et al. *JAMA Neurology*.

eTable 7. Summary of Adverse Events Reported by Local Investigators of the Phase 2a (Safety Population)

Event Description	0.0625 mg/kg of Tenecteplase (N=46)	0.03125 mg/kg of Tenecteplase (N=46)	Control (N=65)	Total (N=157)
Total Events	103	91	126	320
Blood and lymphatic system disorders	6	4	5	15
Cardiac disorders	5	5	10	20
Eye disorders	0	1	0	1
Gastrointestinal disorders	8	10	9	27
General disorders and administration site conditions	1	1	0	2
Infections and infestations	26	25	34	85
Injury, poisoning and procedural complications	5	2	7	14
Metabolism and nutrition disorders	8	5	7	20
Musculoskeletal and connective tissue disorders	1	0	0	1
Nervous system disorders	17	18	26	61
Psychiatric disorders	4	2	2	8
Renal and urinary disorders	6	6	9	21
Respiratory, thoracic and mediastinal disorders	7	4	7	18
Reproductive system and breast disorders	1	0	0	1
Skin and subcutaneous tissue disorders	0	2	2	4
Vascular disorders	8	6	8	22

© 2025 Hou X et al. *JAMA Neurology*.

eFigures

eFigure 1. Distribution of 30 Enrollment Center in China

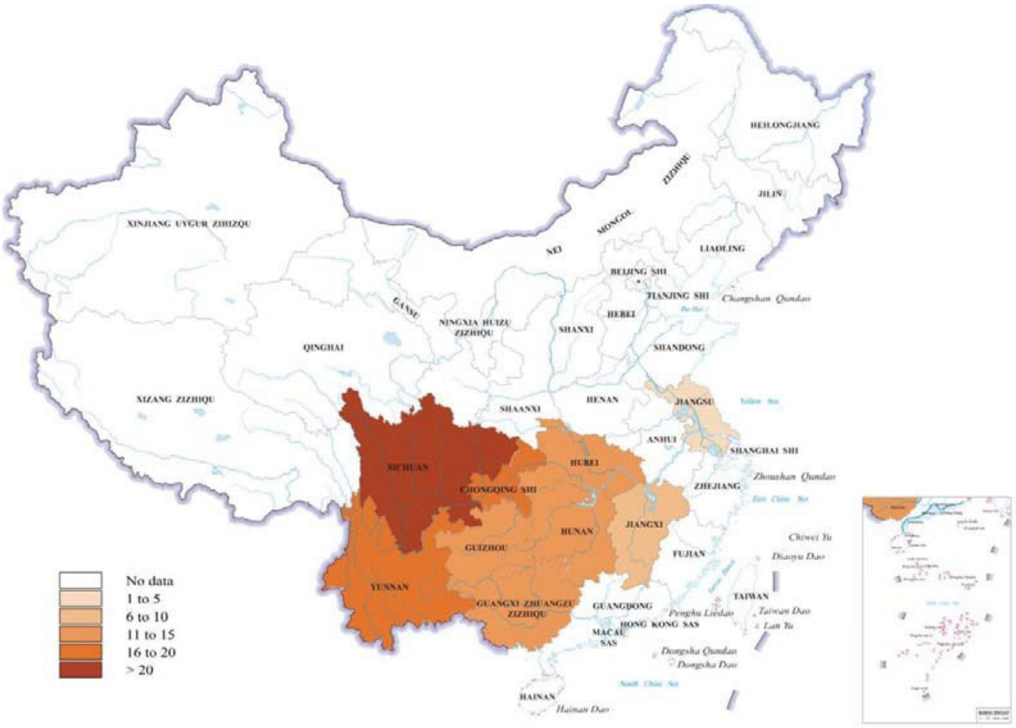

Shown is the distribution of the 30 enrollment centers in China. The colors indicate number of enrollees.

© 2025 Hou X et al. *JAMA Neurology*.

eFigure 2. Distribution of the Modified Rankin Scale score at 90 Days (Phase 1b).

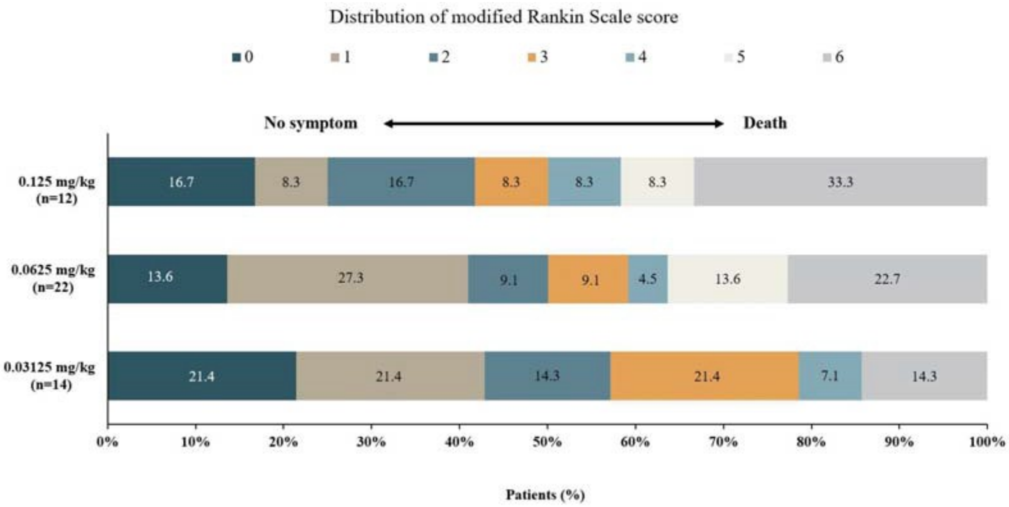

The distribution of the modified Rankin scale score among patients in the intra-arterial tenecteplase group and the control group. Scores range from 0 to 6, with 0 indicating no symptoms, 1, no clinically significant disability, 2, slight disability, 3, moderate disability, 4, moderately severe disability, 5, severe disability, and 6, death. Numbers indicate rounded proportions. Percentages may not total 100 because of rounding.

© 2025 Hou X et al. *JAMA Neurology*.

eFigure 3. Distribution of the Modified Rankin Scale score at 90 Days (Phase 1b+2a).

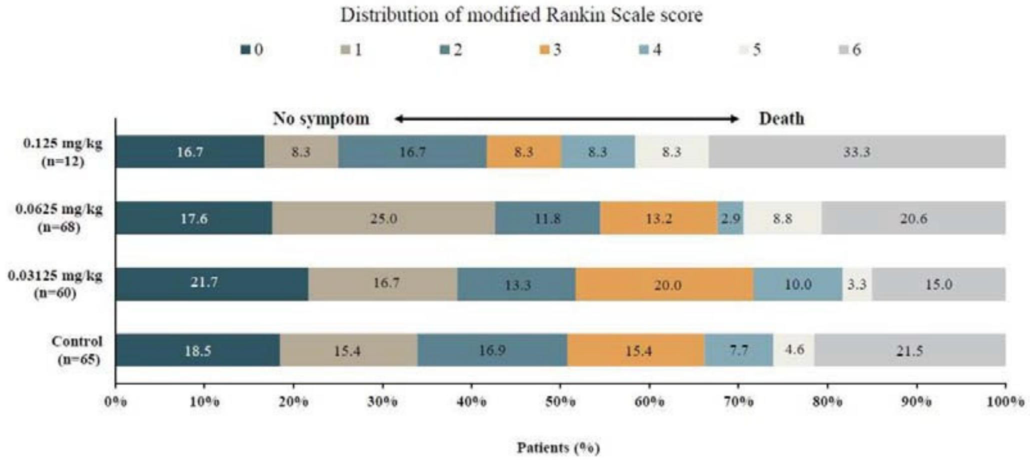

The distribution of the modified Rankin scale score among patients in the intra-arterial tenecteplase group and the control group. Scores range from 0 to 6, with 0 indicating no symptoms, 1, no clinically significant disability, 2, slight disability, 3, moderate disability, 4, moderately severe disability, 5, severe disability, and 6, death. Numbers indicate rounded proportions. Percentages may not total 100 because of rounding.

© 2025 Hou X et al. *JAMA Neurology*.
