# Supplementary material for: Intra-Arterial Tenecteplase After Successful Reperfusion in Large Vessel Occlusion Stroke: A Randomized Clinical Trial
Source: JAMA Neurol. 2025 Jul 5;82(9):895–904. doi: 10.1001/jamaneurol.2025.2036 (PMC12228979; doi:10.1001/jamaneurol.2025.2036)
Supplement: Supplement 4. — Data Sharing Statement. [file jamaneurol-e252036-s004.pdf]

Data Sharing Statement

Hou. Intra-Arterial Tenecteplase After Successful Reperfusion in Large Vessel Occlusion Stroke. *JAMA Neurol*. Published July 05, 2025. doi:10.1001/jamaneurol.2025.2036

Data
Additional Information: ChiCTR.org.cn Identifier: ChiCTR2300073787 and ChiCTR240008062.
Data available: No

Additional Information
Explanation for why data not available: Data from the DATE trial are currently not publicly available but are planned to be made available in the future. The timing of this availability and criteria for gaining access have not been determined. Requests regarding data access should be made to Professor Zhenhua Zhou(zhouzhenhua@tmmu.edu.cn).
